# scXpand: Pan-cancer detection of T cell clonal expansion from single-cell RNA sequencing without paired single-cell TCR sequencing

**DOI:** 10.1016/j.xgen.2026.101328

**Published:** 2026-08-07

**Authors:** Ofir Shorer, Ron Amit, Keren Yizhak

**Affiliations:** 1 Department of Cell Biology and Cancer Science, The Ruth and Bruce Rappaport Faculty of Medicine, Technion - Israel Institute of Technology, Haifa 3525422, Israel

**Keywords:** scRNA-seq, scTCR-seq, T cell clonality, machine learning

## Abstract

Advances in single-cell sequencing have enabled detailed characterization of T cell clonal dynamics in cancer. However, analyses aiming to link the transcriptional landscape to T cell clonality remain limited by confounding factors unequally controlled in different studies. To address this challenge, we developed scXpand, a machine-learning framework for pan-cancer detection of T cell clonal expansion directly from single-cell RNA sequencing (scRNA-seq), without paired T cell receptor (TCR) sequencing. Trained and tested using our in-house-constructed human pan-cancer database of paired scRNA/TCR-seq profiles from 2.6 million T cells, scXpand demonstrates robust and accurate detection of clonal expansion across tissues and T cell subtypes. Applied to datasets lacking TCR sequencing, scXpand predictions correspond with known characteristics of the tumor microenvironment. Overall, scXpand provides a framework for detecting T cell clonal expansion across cancers directly from scRNA-seq, enabling broad use on datasets lacking scTCR-seq, while supporting scalable, memory-efficient processing, including pre-trained models with user-friendly documentation for flexible applications.

## Highlights

• scXpand detects T cell clonal expansion from scRNA-seq without paired scTCR-seq
• scXpand demonstrates robust performance at the pan-cancer level
• scXpand predictions correspond with known characteristics of the TME
• scXpand goes beyond known T cell differentiation states

## Graphical abstract

**Figure:**
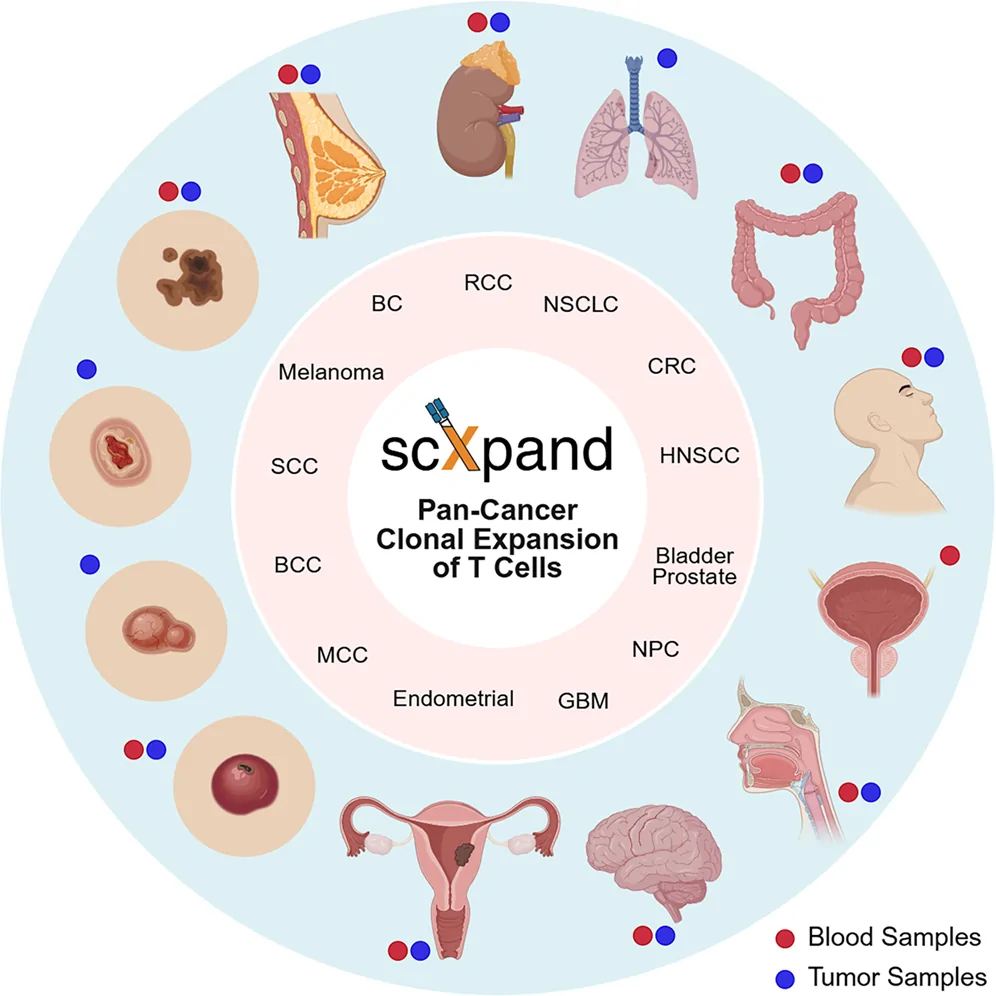

## Teaser

Shorer et al. introduce scXpand, a machine-learning framework for pan-cancer detection of T cell clonal expansion from single-cell RNA sequencing, without paired TCR sequencing. Trained and tested using a human pan-cancer database of 2.6 million T cells, scXpand demonstrates robust and accurate performance across tissues, T cell subtypes, and differentiation states.

## Introduction

Recent advances in single-cell sequencing have enabled detailed characterization of diverse tissues across various pathological conditions, such as cancer. More specifically, single-cell RNA sequencing (scRNA-seq) and single-cell T cell receptor (TCR) sequencing (scTCR-seq) provided a better understanding of T cell immune responses and T cell clonal dynamics in the context of cancer therapy and clinical outcomes.^1^^,^^2^^,^^3^^,^^4^ In turn, identification of expanded T cell clones and their phenotypic landscape, as well as the source of the intra-tumoral TCR repertoire, were previously linked to immunotherapy outcomes across multiple cancer types.^5^^,^^6^^,^^7^^,^^8^ However, despite their unprecedented potential to link transcriptional landscape to T cell clonality, such analyses of paired scRNA/TCR-seq data are still highly limited by confounding and technical factors unequally controlled in different studies.^1^ These include different scopes of TCR capture due to the varying size and location of each tumor sample, as well as sample quality and limited number of productive TCRs following sequencing. As a result, when observing a single T cell having a unique TCR sequence not shared with any other T cell (termed “singleton”), it is uncertain whether such a singleton represents a larger T cell clone masked by these confounding factors. Such a discrepancy can potentially be addressed using the transcriptional phenotype of T cells, enabling the detection of those that are members of expanded clones, even without the availability of TCR sequencing data.

Multiple studies developed computational tools utilizing paired scRNA/TCR-seq datasets, addressing specific aspects of the data to provide additional information for downstream analysis. For example, Zhu et al. developed a deep-learning method (scNAT) to integrate paired scRNA/TCR-seq data and their representations using a latent space for further analysis.^9^ Schattgen et al. introduced clonotype neighbor graph analysis (CoNGA), which identifies correlations between gene expression data and TCR sequencing to elucidate complex relationships between TCR sequence and T cell phenotype.^10^ Zhang et al. developed “tessa,” a tool for integration of scTCR-seq and gene expression, resulting in TCR networks that reflect T cell functional status.^11^ More recently, Tan et al. introduced “predicTCR,” a machine-learning classifier trained on scRNA/TCR-seq data, which identifies individual tumor-reactive T cells based on scRNA-seq data alone.^12^ However, there is currently no method utilizing single-cell data of human patients with cancer for the prediction of T cell clonal expansion at the pan-cancer level, spanning multiple tissues and subtypes of T cells.

To address this challenge, we developed scXpand, a machine-learning framework trained and tested using our in-house-constructed pan-cancer database of paired scRNA/TCR-seq profiles from 2.6 million T cells. Our framework is trained on data from more than 1.5 million T cells, obtained from human patients with cancer across 963 tumor and blood samples, representing 14 different cancer types. Tested on additional single-cell data of more than 1 million T cells from 482 samples external to the model, we showed that scXpand provides robust and accurate pan-cancer detection of T cell clonal expansion from scRNA-seq data without paired TCR sequencing. It demonstrates high performance across tumor and blood samples separately, as well as across different T cell subtypes. In addition, we created user-friendly documentation for our framework, enabling users to directly apply it to any given dataset and modify it for other, more complex classification tasks using our built-in optimization procedure. This setting enables a flexible foundation for broader applications according to user preferences. Moreover, our framework provides scalable processing for handling millions of single cells with memory-efficient data streaming from disk during training, with access to already trained and optimized models for direct application of the platform. Our platform has also been incorporated into the “scverse” ecosystem^13^ and is registered as an ecosystem package.

## Results

### Constructing a comprehensive pan-cancer scRNA/TCR-seq database of T cells

**Figure 1:**
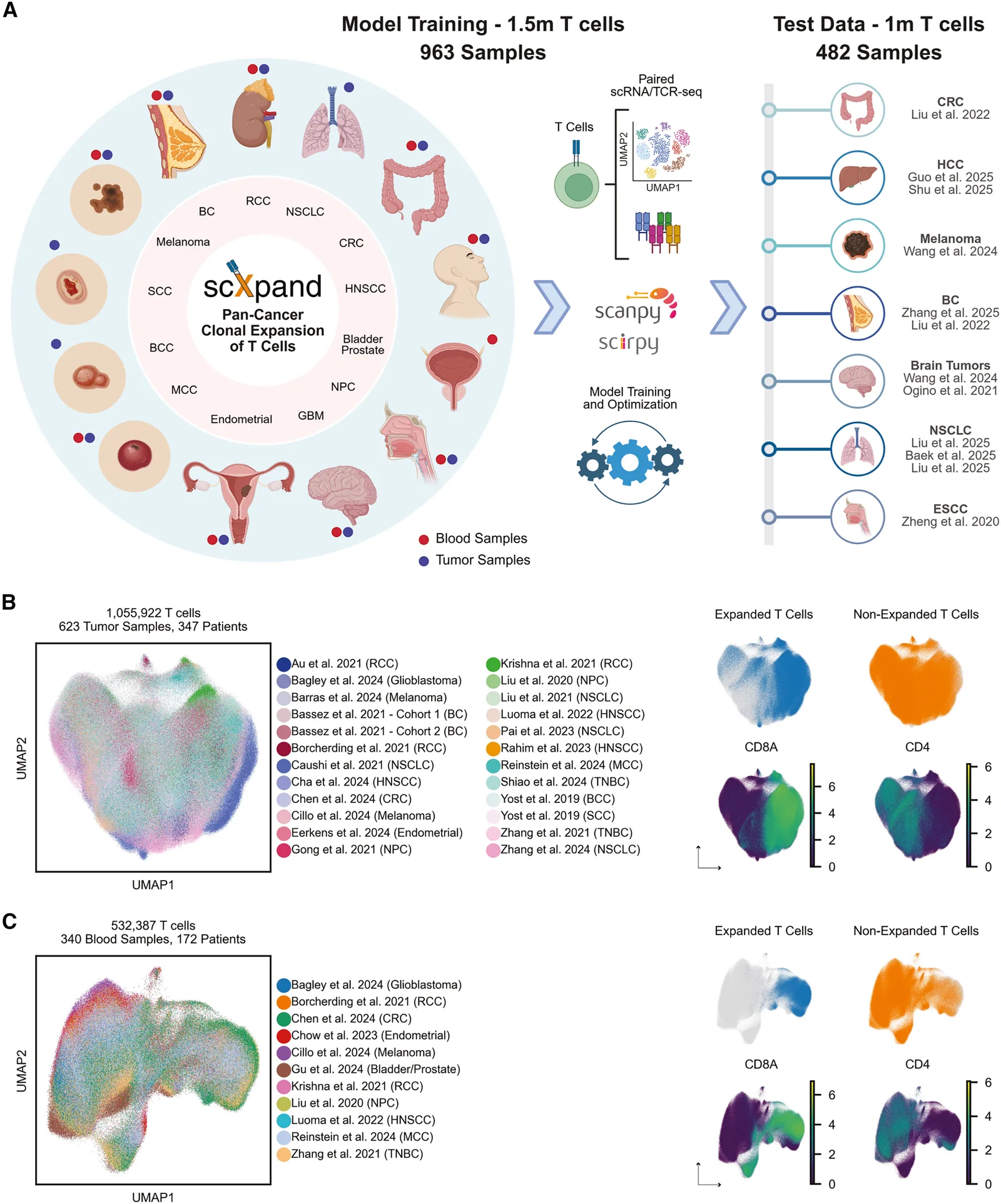
Constructing a comprehensive pan-cancer scRNA/TCR-seq database of T cells (A) A schematic workflow of the study depicting T cell data collection across tumor and blood samples of human patients with cancer, strict quality control, model training, and application to additional test datasets. (B) Uniform manifold approximation and projection (UMAP) plot of 1,055,922 T cells from tumor samples with paired scRNA/TCR-seq used for model training (left) and annotations for clonal expansion as well as expression of *CD8A* and *CD4* (right). (C) UMAP plot of 532,387 T cells from blood samples with paired scRNA/TCR-seq used for model training (left) and annotations for clonal expansion as well as expression of *CD8A* and *CD4* (right). See also Figure S1 and Table S1.

To train a model to predict T cell clonal expansion at the pan-cancer level, we first collected publicly available data of tumor and blood samples obtained from patients with cancer across 14 cancer types, consisting of 38 datasets with more than 2.6 million T cells passing a strict quality control done separately on both scRNA-seq and scTCR-seq data^5^^,^^6^^,^^14^^,^^15^^,^^16^^,^^17^^,^^18^^,^^19^^,^^20^^,^^21^^,^^22^^,^^23^^,^^24^^,^^25^^,^^26^^,^^27^^,^^28^^,^^29^^,^^30^^,^^31^^,^^32^^,^^33^^,^^34^^,^^35^^,^^36^^,^^37^^,^^38^^,^^39^^,^^40^^,^^41^^,^^42^^,^^43^^,^^44^^,^^45^^,^^46^^,^^47^ (Figure 1A; Table S1; STAR Methods). Overall, 26 datasets containing 963 samples with more than 1.5 million T cells were used to train the model, with an additional 1 million T cells across 482 samples from 12 other datasets used to test model performance on each dataset independently (Figure 1A; Table S1). Across the training set, 11,950 genes shared between all datasets passed the quality control process and were used for further integration of all datasets (Figures 1B and 1C; STAR Methods). Markov affinity-based graph imputation of cells (MAGIC)^48^ was first applied in order to detect possible dropouts of *CD8A/B*, *CD4*, or *FOXP3* (Figures S1A–S1D; STAR Methods). Following this, single cells were labeled according to their membership in expanded or non-expanded T cell clones, using CDR3 sequence identity, and their clone size per sample was quantified accordingly (Figures 1B and 1C; STAR Methods). Expanded T cell clones were defined using sample-wise thresholding (STAR Methods), thus normalizing for differences in sampling depth and repertoire complexity between samples while reducing biases introduced by fixed-clone-size thresholds. This approach enabled the identification of expanded clones with potential functional impact. This database was then prepared as an input to the model, such that the input features include a gene expression matrix containing unique molecular identifier (UMI) counts for each T cell, with gene representation using Ensembl IDs^49^ for generalization purposes and a simpler user interface. The target binary variable of each T cell is its membership in either expanded or non-expanded T cell clones. In addition, the tissue of origin for each T cell (i.e., tumor or blood), as well as the T cell subtype following cell imputation (i.e., CD8^+^, CD4^+^, regulatory T cell [Treg], double negative, or double positive), were also included as part of the model optimization process (STAR Methods). Importantly, training incorporates gene expression data together with expansion status derived from scTCR-seq data, whereas inference requires scRNA-seq data alone. Open-source tutorials demonstrating how to properly prepare the model input for both training and inference are provided as part of the scXpand package.

### Prediction of T cell clonal expansion using scXpand

**Figure 2:**
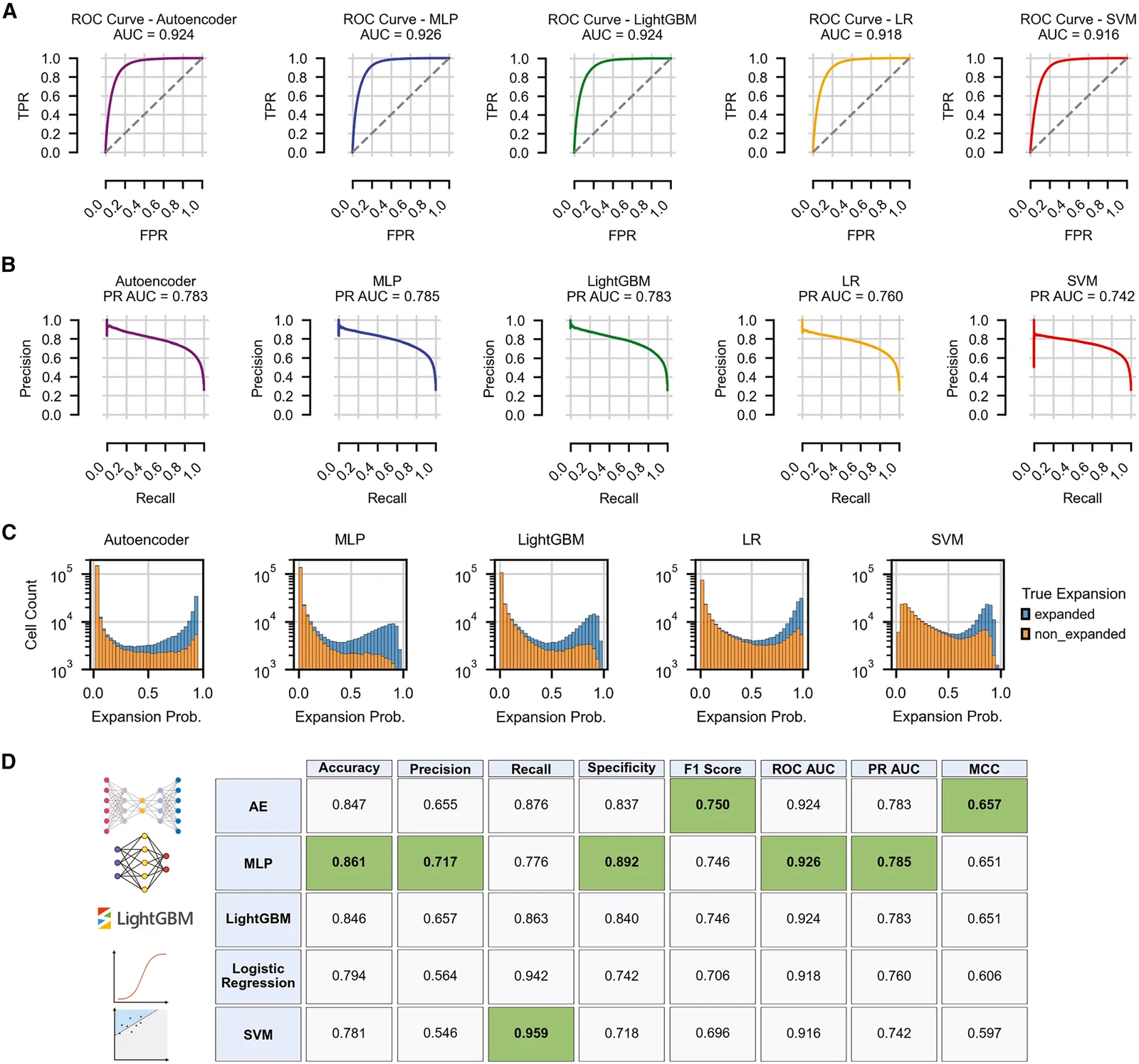
Prediction of T cell clonal expansion using scXpand (A) Receiver operating characteristic (ROC) curves, with calculation of the corresponding area under the curves (AUCs), for each one of the five trained and optimized models, using the validation set. (B) Precision-recall (PR) curves, with calculation of the corresponding AUCs. (C) Histograms depicting the number of T cells from the validation set, across the predicted expansion probabilities obtained for each T cell by each model. Bars are colored and stacked based on the true expansion labels of each T cell. (D) Model performance metrics measured using the validation set, across all five trained models. FPR, false positive rate; TPR, true positive rate; MLP, multilayer perceptron; LR, logistic regression; SVM, support vector machine; MCC, Matthews correlation coefficient. See also Figures S2–S7 and Table S2.

The scXpand framework for prediction of T cell clonal expansion supports multiple modeling strategies, including linear (support vector machine [SVM] and logistic regression), as well as tree-based (LightGBM^50^) and deep-learning (multilayer perceptron [MLP]) models (STAR Methods). Alongside these standard off-the-shelf methods, we developed a new masked multi-task, forked count autoencoder specifically tailored to the properties of scRNA-seq data. This autoencoder denoises the input by modeling the count distribution and sparsity using a zero-inflated negative-binomial noise model, similar to Eraslan et al.^51^ On top of this autoencoder core, we added two additional branches stemming out of the latent vector to form a multi-task model. The first branch includes an auxiliary classification task of identifying the T cell tissue of origin (i.e., tumor or blood), with the second branch consisting of an integrated classification head for the main task of predicting T cell clonal expansion (Figure S2). This multi-task approach learns tasks in parallel while using a shared representation, potentially improving the task-specific performance.^52^ Importantly, the autoencoder architecture, hyperparameter configuration, and inclusion of the auxiliary classification task, including configurations of the other four models, were identified through an automated, data-driven tuning and optimization process, independent of human input (Figures S3A–S3F; Table S2; STAR Methods). Our framework is designed to offer multiple complementary models rather than a single prescribed choice, providing flexibility to users while demonstrating that the predictive signal is robust and not driven by a single modeling approach. This design allows users to directly compare or combine predictions across methods, depending on their analytical goals, rather than enforcing a single “best” model. Following this, we provide a thorough comparison of these optimized models, as detailed below (Figure 2; Table S2).

From the training set of 1.5 million T cells, we allocated 80% of the data for training and 20% for validation, used for hyperparameter tuning and model optimization (STAR Methods). Optimized model performance was then evaluated on the validation set (Figure 2). Of note, we employed a stratified data allocation strategy to ensure that cell populations were proportionally represented in both the training and validation sets while enforcing that all cells from a given patient are assigned exclusively to either the training or validation set but not both (STAR Methods). For each optimized model, we constructed the receiver operating characteristic (ROC) curve and the precision-recall (PR) curve and calculated the area under these curves (AUCs). Interestingly, the performance was similar across the different models, with no substantial differences observed in the area under the ROC (AUROC; 0.916–0.926; Figure 2A; Table S2) or the PR (AUPRC; 0.742–0.785; Figure 2B; Table S2) curves. However, the AUROC and AUPRC only capture ranking performance across thresholds and can therefore obscure meaningful differences in classification accuracy at a fixed threshold. To overcome this limitation, we used the predicted expansion probability distribution of each model (Figure 2C) to assess accuracy, precision, recall, specificity, and F1 score, with a naive expansion probability threshold of 0.5 (Figure 2D). These additional measures revealed clear differences: some models achieved higher precision but at the cost of lower recall, whereas others showed the opposite trend. Specifically, while the autoencoder, MLP, and LightGBM models showed lower false positive rates (0.108–0.163; Table S2), both the logistic regression and the SVM models demonstrated higher false positive rates (0.258–0.282; Table S2). These rates were mirrored by higher and lower false negative rates, respectively (Table S2). These results highlight that despite similar overall discrimination, the models varied in their ability to correctly identify either expanded or non-expanded T cells.

To further evaluate model performance, and since there is a general imbalance of expanded and non-expanded T cells in the data, we calculated an additional metric, the Matthews correlation coefficient (MCC), which takes these imbalanced populations into account^53^^,^^54^ (Figure 2D). In order to address optimal expansion probability thresholds in each model, we calculated the MCC value distribution in each model for each T cell subtype in both tumor and blood samples (Figures S4A–S4E). This analysis resulted in different optimal thresholds per model, though MCC values remained comparable across models (Figures S4A–S4E). These results emphasize the robustness of the different models in capturing clonal expansion despite data imbalance and show that while the optimal thresholds may vary across models, their relative performance remains consistent.

We next evaluated model performance after modifying our expansion labeling strategy from a sample-level to a patient-level approach. Labeling expanded clones per patient groups T cells sharing identical CDR3 sequences across all the samples obtained from the same patient (Figure S5A). The predictions obtained using a model re-trained with the patient-wise labels were comparable to those obtained using the original sample-wise training and significantly correlated with them (Spearman r = 0.985, *p* << 0.05; Figures S5B and S5C; STAR Methods). This result indicates that the different labeling approaches do not introduce systematic bias in the model’s detection of clonal expansion. While the results remained unchanged, we believe that the sample-wise approach for detecting clonal expansion is the correct one. In different contexts and environments, T cells from the same clone can receive different signals, affecting their functionality and, consequently, their clonal expansion. These include signals received only upon infiltration into the tumor, as well as signals affected by treatment regimens or by different environmental compositions, as previously suggested.^8^ As such, distinct environmental contexts can result in different transcriptional states of T cells, including clonal expansion, despite sharing the same clonal identity.

To examine model performance for different epitopes, we further annotated the epitope of each single cell based on the CDR3 amino acid sequence using VDJdb^55^ (Figure S6A; STAR Methods). By applying our models separately to virus-specific T cells and to T cells that do not target any known non-cancerous antigens, we demonstrated that clonal expansion can be reliably detected by our models in both populations (Figures S6B and S6C). This result highlights the broad applicability of our models to both cancer- and virus-specific T cells, regardless of their antigenic context. Specifically, it points to potential future applications of our framework in the context of virus-specific memory T cells that are repurposed for cancer immunotherapy.^56^ Importantly, clonal expansion was detected within each group despite transcriptional differences between expanded T cells from the two groups, most notably the significant expression of *CXCL13* in the non-virus-specific group (Figure S7A), consistent with its previously reported association with tumor-reactive T cells.^57^^,^^58^ However, when considering virus-specific T cells alone, expanded T cells demonstrate significant expression of *CXCL13* compared to non-expanded T cells from the same group, together with additional markers associated with clonal expansion (Figures S7B and S7C), as described in the following sections. Thus, the model does not explicitly distinguish virus-specific expanded T cells from other expanded T cell populations but rather reliably detects clonal expansion within and across these groups, despite their relative transcriptional differences.

Taken together, scXpand enables robust detection of T cell clonal expansion and lays the foundation for broader applications in immuno-oncology and cancer research. Furthermore, the optimized complex and simpler models achieve comparable performance using their optimal thresholds, suggesting that the predictive signal is largely linear and that increased model complexity, such as that of the autoencoder, offers limited added value. However, the inclusion of multiple model architectures reflects the exploratory nature of this previously untested prediction task, providing a generalizable framework for predicting T cell clonal expansion from scRNA-seq data.

### Application of the model to external test datasets

**Figure 3:**
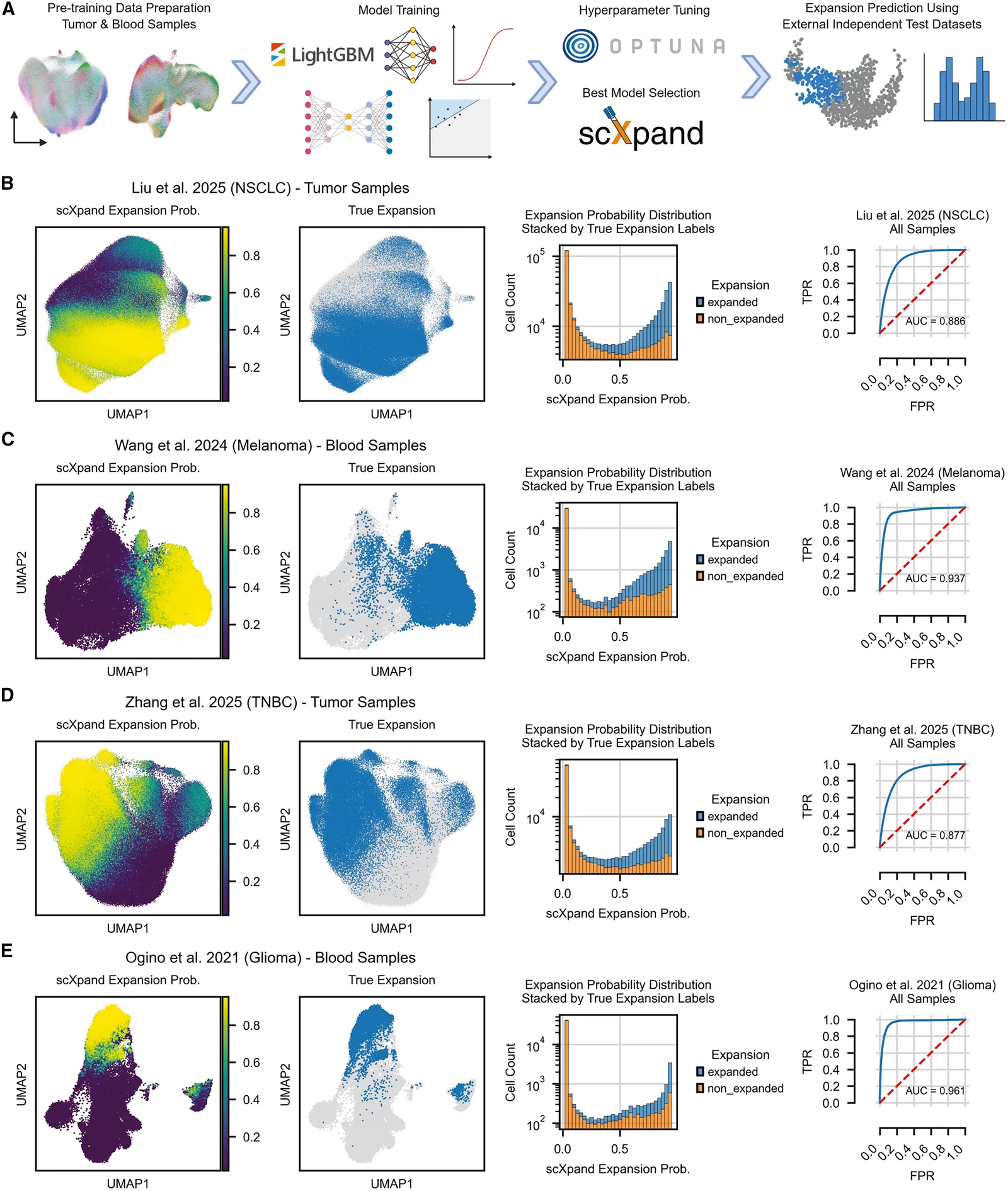
Application of the model to external test datasets (A) A schematic workflow demonstrating the model training and hyperparameter tuning process, with application of the optimized trained models using an additional collection of test datasets that were external to the model and were not used for training. (B–E) Application of the trained autoencoder model to four different test datasets,^39^^,^^40^^,^^43^^,^^44^ consisting of different cancer types across tumor and blood samples. UMAP plots demonstrating the predicted expansion probability for each T cell, as well as the true expansion labels (left), including a histogram of the probability distribution stacked by the true expansion labels, with the corresponding ROC curves (right). FPR, false positive rate; TPR, true positive rate. See also Figures S8–S15 and Tables S1 and S3.

To test model performance, we applied our optimized models to an additional 12 test datasets having paired scRNA/TCR-seq, consisting of 482 samples with more than 1 million T cells (Figures 1A, 3A, and S8–S10; Tables S1 and S3). Importantly, all of these datasets were external to the model and were not used as part of the training and hyperparameter tuning procedures. Applying the autoencoder model to datasets having either tumor or blood samples, as well as to datasets having both sample types, demonstrated robustly accurate results (Figures 3B–3E and S8–S10; Table S3). Specifically, for T cells obtained from tumor samples of patients with non-small cell lung cancer (NSCLC),^44^ our model achieved an AUC of 0.886 (Figure 3B). When tested on blood samples from patients with melanoma,^39^ it achieved an AUC of 0.937 (Figure 3C). In two additional datasets of tumor samples from patients with triple-negative breast cancer (TNBC)^40^ and blood samples from patients with glioma,^43^ our model achieved an AUC of 0.877 and 0.961, respectively (Figures 3D and 3E). High AUCs (0.847–0.963) were obtained for an additional eight datasets of brain tumors,^36^ colorectal cancer,^37^ lung cancer,^45^^,^^46^ esophageal cancer,^41^ and breast cancer,^42^ as well as two datasets of patients with hepatocellular carcinoma (HCC),^38^^,^^47^ despite not having been trained on the latter cancer type (Figures S8–S10). As expected, and consistent with the training set, all other models included in our framework exhibited comparable overall performance across datasets with a similar trade-off between precision and recall (Table S3). Moreover, a subsampling experiment considering different sizes of the training set showed significantly improved performance over the test datasets when using 100% of the training set, demonstrating the added value of the large-scale database used for training (Figures S11A and S11B; STAR Methods).

To test the robustness of scXpand to different expansion thresholds, we performed two analyses: first, we used our models that were pre-trained on a single threshold and evaluated their predictive ability in the validation set where clones were defined using alternative expansion thresholds. Interestingly, although clonal expansion labels are sensitive to the selected expansion threshold (Figures S12A and S12B), all models maintained robust validation AUROCs (Figure S12C; STAR Methods). Consistent with this, across all five trained models, the mean predicted expansion probability per clone was significantly correlated with clone size (Spearman r = 0.319–0.341, *p* << 0.05; Figure S12D). These findings indicate that the models capture clonal expansion as a continuous biological signal rather than a strictly binary state, thereby explaining their robust performance across different expansion thresholds. Second, we re-trained all models using alternative expansion thresholds (Figures S11C–S11I and S12E) and found that all models achieve high and robust validation AUROCs, further supporting the conclusion that the models learn a continuous expansion-associated signal that is consistently captured across models.

Next, as clonal expansion is mostly attributed to expansion of CD8^+^ T cells (Figure S13A), we evaluated model performance across the test data for different T cell subtypes, including CD8^+^, CD4^+^, Treg, double positive, and double negative. This was done in two ways, considering both tumor and blood samples: first, we utilized models trained on all data but examined their performance on each T cell subtype separately. This analysis showed that our models can well discriminate clonal expansion within individual T cell subtypes across the test data (Table S3). Second, we trained subtype-specific models for CD8^+^ or CD4^+^ T cells alone (Figures S13B–S13E; STAR Methods). We found that while prediction probability distributions vary between the model trained on all data and the two subtype-specific models, their prediction probabilities are significantly correlated in each T cell subtype separately (Spearman r = 0.950 and 0.919 for CD8^+^ and CD4^+^ T cell clones, respectively; Figure S14). A similar analysis was performed to compare model performance for tumor and blood samples separately, regardless of any T cell subtype (Figures S15A–S15C). This analysis also demonstrated a significant correlation in model probabilities (Spearman r = 0.917 and 0.629 for tumor and blood samples, respectively; Figure S15D). Overall, our five-model framework demonstrates robust and accurate performance across different datasets and cancer types, including both tumor and blood samples. These results highlight its strong generalizability and imply its applicability in diverse contexts at the pan-cancer level.

### Model explainability and biological interpretation

In order to further understand the model output and obtain biological interpretation of its predictions, we employed the Shapley additive explanations (SHAP) values,^59^ which quantify the contribution of individual genes to the predicted clonal expansion status of each T cell, measuring both the magnitude and directionality of their effect on the model predictions. Furthermore, this approach reveals more intricate context-dependent gene behavior, capturing effects of gene-gene interactions and their influence on the model predictions, allowing us to identify key features highly associated with expanded or non-expanded T cell clones.

**Figure 4:**
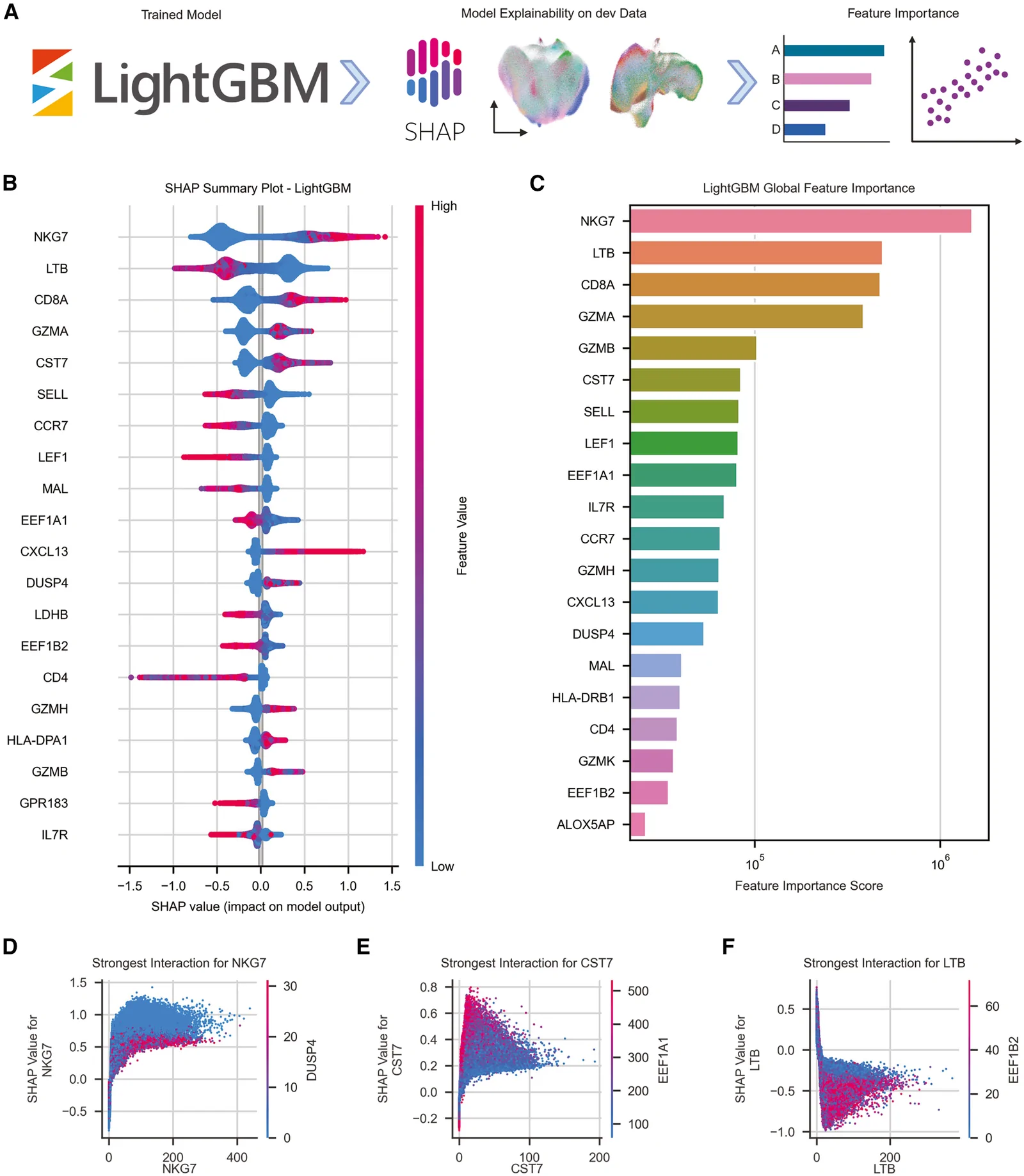
Model explainability and biological interpretation (A) A schematic workflow demonstrating the application of SHAP^59^ to the LightGBM model, using the validation set. (B) SHAP summary plot depicting the top 20 important genes driving the LightGBM model predictions. (C) Global feature importance of the LightGBM model, reflecting the overall contribution of the top 20 important genes to the predictive performance of the model. (D–F) SHAP dependency plots showing the most interactive partners of selected genes, including *NKG7*, *CST7*, and *LTB*. Expression values represent row-normalized UMI counts, without log-transformation or *Z* score normalization. See also Figures S16–S18 and Table S2.

Because SHAP value computation has been directly integrated into the LightGBM codebase,^60^ we performed this analysis using that model (Figure 4A). As expected, the SHAP analysis showed that high expression of genes such as *NKG7*, *CD8A*, *GZMA*, and *CST7* was driving the model predictions toward expanded status, while high expression of genes such as *LTB*, *SELL*, *CCR7*, *LEF1*, *MAL*, and *EEF1A1* was driving the predictions toward the non-expanded status (Figure 4B). This list of genes corresponded well with the ranking achieved by the global feature importance derived from the LightGBM “gain” metric, reflecting the overall contribution of each gene to the predictive performance of the model (Figure 4C). Importantly, however, these genes alone cannot be used for prediction, as ∼12% of the expanded T cells do not express *NKG7*, while ∼30% of them express *LTB*. In addition, ∼23% of the non-expanded T cells do not express *LTB*, while ∼28% of them express *NKG7*, as measured for the validation set. In fact, in the next section we show that an activation-exhaustion vs. naive-memory signature is inferior to the complex signature captured by scXpand, including a streamlined gene signature composed of the top predictive markers from our SHAP analysis, indicating that the model captures higher-order transcriptional patterns that extend beyond simple marker-based definitions. Finally, applying a separate SHAP value computation for CD8^+^ and CD4^+^ T cells from tumor samples resulted in a modified yet similar list of genes driving the model predictions (Figures S16A and S16B), thus showing genes driving expansion status across T cell populations, as well as more subtype-specific genes.

Going beyond the impact of single genes and their effect on the model predictions, we next examined SHAP values for gene-gene interactions. To this end, we focused on the top genes having the highest absolute Shapley scores and examined their strongest interactive partners (Figures 4D–4F). This process resulted in notable gene pairs demonstrating context-dependent effects: the SHAP value for a gene can differ between two cells with the same expression level of that gene, resulting from the contextual expression of other genes in these cells. More specifically, while high expression of both *NKG7* and *DUSP4* resulted in higher SHAP values separately (Figure 4B), the interaction between both genes demonstrates that high expression of *DUSP4* in the context of *NKG7* expression reduces SHAP values (Figure 4D). A potential explanation is that *NKG7*, a cytotoxic granule-associated gene linked to effector activity, is attenuated by *DUSP4*, a dual-specificity phosphatase implicated in negative regulation of *MAPK* signaling and T cell activation. Thus, when *DUSP4* is highly expressed in a cell already exhibiting high *NKG7*, it may act as a counter-regulatory brake, diminishing the predicted likelihood of clonal expansion. In contrast, our analysis showed that while high expression of *EEF1A1* resulted in low SHAP values (Figure 4B), high expression of *EEF1A1* in the context of *CST7* expression can result in increased SHAP values (Figure 4E). This interaction between *EEF1A1* and *CST7* could indicate that high translational activity induced by *EEF1A1* becomes favorable for expansion specifically in cytotoxic, *CST7*^*high*^ T cells, whereas the same *EEF1A1* signature outside this cytotoxic context does not support clonal expansion. Finally, while high expression of both *LTB* and *EEF1B2* resulted in lower SHAP values (Figure 4B), their contextual expression demonstrates a synergistic negative effect on predicted clonal expansion (Figure 4F).

To further explore higher-level biological mechanisms related to clonal expansion, we performed a pathway enrichment analysis using the GSEApy “enrichr” module,^61^ applied to the top explainable genes obtained using SHAP for expanded and non-expanded T cells from the validation set (Table S2; STAR Methods). In expanded T cells, we found enrichment of pathways such as TCR signaling (*p* = 2.32 × 10^−8^), downstream TCR signaling (*p* = 4.83 × 10^−6^), and CD28 co-stimulation (*p* = 8.81 × 10^−6^), as well as pathways related to T cell mitosis, including M phase, mitotic anaphase, separation of sister chromatids, and mitotic metaphase (Figure S17A; Table S2). In contrast, non-expanded T cells were enriched with pathways such as cellular senescence (*p* = 0.002) and development of Tregs (*p* = 0.001), as well as pathways related to DNA repair (Figure S17B; Table S2). This observation corresponds to the distinct functional states of the two populations, where expanded T cells express genes associated with activation and proliferation, while non-expanded T cells show enrichment of pathways linked to immune regulation and cellular maintenance. Overall, these results highlight a more interpretable perspective on the top explainable genes obtained by the model. However, it is important to emphasize that these two functional states appear across multiple T cell differentiation states, as described in the following section. Finally, as expected, SHAP value computation using a simple model such as logistic regression resulted in the capture of simple linear trends between gene expression and SHAP values (Figures S18A and S18B). These results emphasize the trade-off between model complexity, interpretability, and the ability to capture more complex context-dependent interactions between features. Overall, by using SHAP for model explainability, we demonstrate the capture of previously known biological effects of gene expression and T cell clonality, as well as more intricate, non-trivial gene-gene interactions affecting model predictions.

### scXpand surpasses T cell differentiation-based classification and existing tumor-reactivity signatures

**Figure 5:**
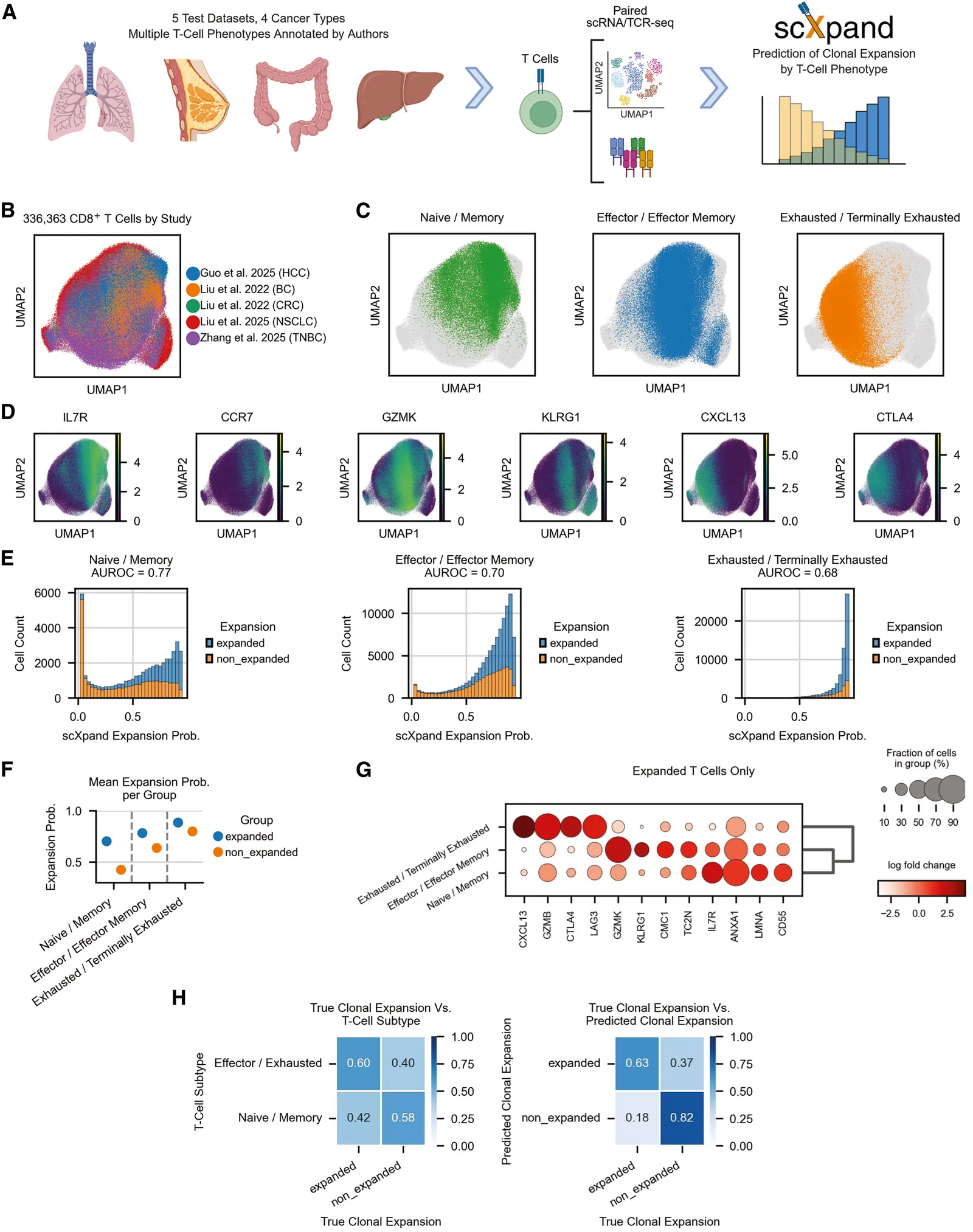
scXpand surpasses T cell differentiation-based classification and existing tumor-reactivity signatures (A) A schematic workflow demonstrating the selection of the five annotated test datasets^37^^,^^38^^,^^40^^,^^42^^,^^44^ and application of scXpand across different T cell differentiation states. (B) UMAP plot of 336,363 CD8^+^ T cells across 359 tumor samples from the five test datasets with paired scRNA/TCR-seq. (C) UMAP plots showing the three selected T cell differentiation states as originally annotated by the authors. (D) UMAP plots demonstrating the gene expression of multiple selected markers. (E) Histograms depicting the number of T cells in each differentiation state, across the predicted expansion probabilities obtained for each T cell by the autoencoder. Bars are colored and stacked based on the true expansion labels of each T cell. (F) Mean expansion probability predicted by the autoencoder per T cell differentiation state, separated by true labels for expanded and non-expanded T cells. (G) Dotplot showing the top 4 differentially expressed genes per T cell differentiation state, focusing only on expanded T cells. (H) Confusion matrices showing the fraction of the true clonal expansion status by T cell differentiation state (left), as well as according to the predicted clonal expansion status using the autoencoder with a naive threshold of 0.5 (right). See also Figures S19–S25 and Tables S1 and S3.

As some key genes among the top drivers of the model predictions are classic markers of T cell differentiation states (*IL7R*, *CCR7*, *SELL*, and *NKG7*; Figures 4B and 4C), we examined whether our framework can detect clonal expansion within different T cell states, thus showing that scXpand functions as a true predictor of clonal expansion rather than a simple T cell differentiation classifier. We therefore focused on 5 test datasets (out of the 12 evaluated test datasets) in which the authors provided publicly available annotations for T cell differentiation states^37^^,^^38^^,^^40^^,^^42^^,^^44^ (Figures 5A and 5B; Tables S1 and S3; STAR Methods). Overall, these datasets contained 336,363 CD8^+^ T cells across 359 tumor samples, of which we focused on three differentiation states: (1) naive/memory, (2) effector/effector-memory, and (3) exhausted/terminally exhausted T cells (Figures 5C and 5D; Table S3). It is important to note that a single expanded clone can be composed of all T cell states altogether in varying fractions (Figures S19A–S19C). Consequently, whenever a clone was labeled as expanded, we referred to the different T cells originating from this clone as expanded as well, regardless of their differentiation state. We then used our optimized autoencoder model and measured its predictions across these three T cell differentiation states separately (Figure 5E). Indeed, the autoencoder managed to separate expanded from non-expanded T cells, with the best performance demonstrated for the naive/memory group (AUROC = 0.77; Figure 5E). Moreover, this differentiation state demonstrated the largest difference in the mean predicted expansion probability between expanded and non-expanded T cells (Figure 5F). Differential gene expression calculated when focusing solely on expanded T cells in each group showed that, indeed, T cell clonal expansion goes beyond classic T cell differentiation markers, as expanded T cells can express both markers of naive/memory T cells, as well as T cell exhaustion markers (Figure 5G). Most importantly, we showed that classification of clonal expansion based on T cell differentiation states is inferior to the classification achieved by scXpand with a naive probability threshold of 0.5 (Figure 5H).

Next, we performed a separate SHAP analysis within each T cell differentiation state. We found that the majority of the top explanatory genes were broadly shared across states (Figures S20A–S20C; Table S3; STAR Methods). While many genes overlapped, their relative rankings varied between differentiation states, and each state also exhibited a smaller set of uniquely prioritized genes. Importantly, though, most of these unique genes were not among the top 50 explainable genes per differentiation state (Table S3). Moreover, while a similar set of genes was found to drive clonal expansion across different differentiation states, the expression levels of these genes varied substantially between states (Figure S20D). This suggests that the expansion-associated signal captured by scXpand is interpreted relative to the transcriptional baseline of each differentiation state, consistent with its ability to model clonal expansion as a continuous signal.

Notably, the inferior performance observed for the exhausted/terminally exhausted T cell group likely reflects limitations of scTCR-seq sampling rather than model misclassification. Specifically, some T cells are often underrepresented in scTCR-seq due to lower transcript abundance and incomplete capture of clonally related cells. As a result, truly expanded exhausted clones may be annotated as non-expanded simply because other members of the clone were not sequenced. In such cases, scXpand may classify these cells as expanded based on their full transcriptional program, which includes features characteristic of proliferation history or prior activation. Thus, what appears to be a misclassification relative to the incomplete ground truth may in fact represent a biologically accurate prediction of their true expansion status. To examine this point in our data, we evaluated the proportion of singletons and non-singleton T cells in each differentiation state, across our pre-defined expanded/non-expanded criteria. For this analysis, singletons were defined as clone size = 1 and non-singletons as clone size > 1. This is in contrast to the definition of expanded and non-expanded clones that represent a per-sample normalization (STAR Methods). We found that the exhausted/terminally exhausted population has the highest proportion of non-singletons among non-expanded T cells, compared to all other T cell phenotypes (Figure S21A), demonstrating that exhausted T cells tend to exhibit higher levels of baseline clonality. Following this, when explicitly accounting for the separation between singletons and non-singletons within each T cell state, we observed high model performance (Figure S21B).

To further demonstrate this point, we compared the transcriptional signature between expanded and non-expanded T cells in the different differentiation states. While a similar signature was found between both groups within the exhausted state, naive/memory T cells demonstrated a clear distinction between the two expansion groups (Figures S22A–S22C). However, stratification of cells by the model-predicted expansion status within the exhausted T cell population revealed clear transcriptional differences (Figure S22D). This finding suggests that model-derived predictions provide higher-resolution discrimination of clonal expansion within this T cell state, highlighting the potential limitations of TCR-based expansion annotations. Consistent with this finding, we found that higher expansion probabilities are assigned to T cells from larger clones across all groups (Figure S22E).

We next compared the performance of scXpand with a publicly available signature of neoantigen-reactive tumor-infiltrating CD8^+^ T cells^58^ and used the 243-gene version of the “NeoTCR8” signature provided by the authors, scoring each CD8^+^ T cell per differentiation state (STAR Methods). Across all states, this signature did not manage to properly differentiate between expanded and non-expanded CD8^+^ T cells, demonstrating lower AUROCs compared to scXpand (Figure S23A). We made similar comparisons with three additional signatures of tumor reactivity (Figures S23B–S23H), including the “MANAscore” devised by Zeng et al.^57^ (Figure S23B), the “TR30” signature by Meng et al.^62^ (Figure S23C), and the “CD8 Transcriptome Signature” by Hanada et al.^63^ (Figure S23D; STAR Methods). Similar to the NeoTCR8 signature, all three additional signatures showed lower performance compared to scXpand. Importantly, the MANAscore was inferior to scXpand in predicting T cell clonal expansion for both viral- and non-viral-specific T cells (Figures S24A and S24B; STAR Methods).

Finally, we constructed two streamlined gene signatures composed of the top 5 genes obtained by the SHAP analysis of scXpand (Figure 4B), resulting in a signature associated with clonal expansion (*NKG7*, *CD8A*, *GZMA*, *CST7*, and *CXCL13*) and a signature associated with non-expansion (*LTB*, *SELL*, *CCR7*, *LEF1*, and *MAL*). Similar to the other evaluated signatures, both constructed signatures demonstrated inferior performance compared to scXpand (Figures S25A–S25C; STAR Methods). Overall, these results reveal that scXpand can resolve clonal expansion patterns that are not recoverable using standard immunological state annotations or existing tumor reactivity signatures, including streamlined gene signatures captured by our model, underscoring the non-trivial nature of this prediction task and the added value of our framework.

### Applicable examples of using the scXpand framework

**Figure 6:**
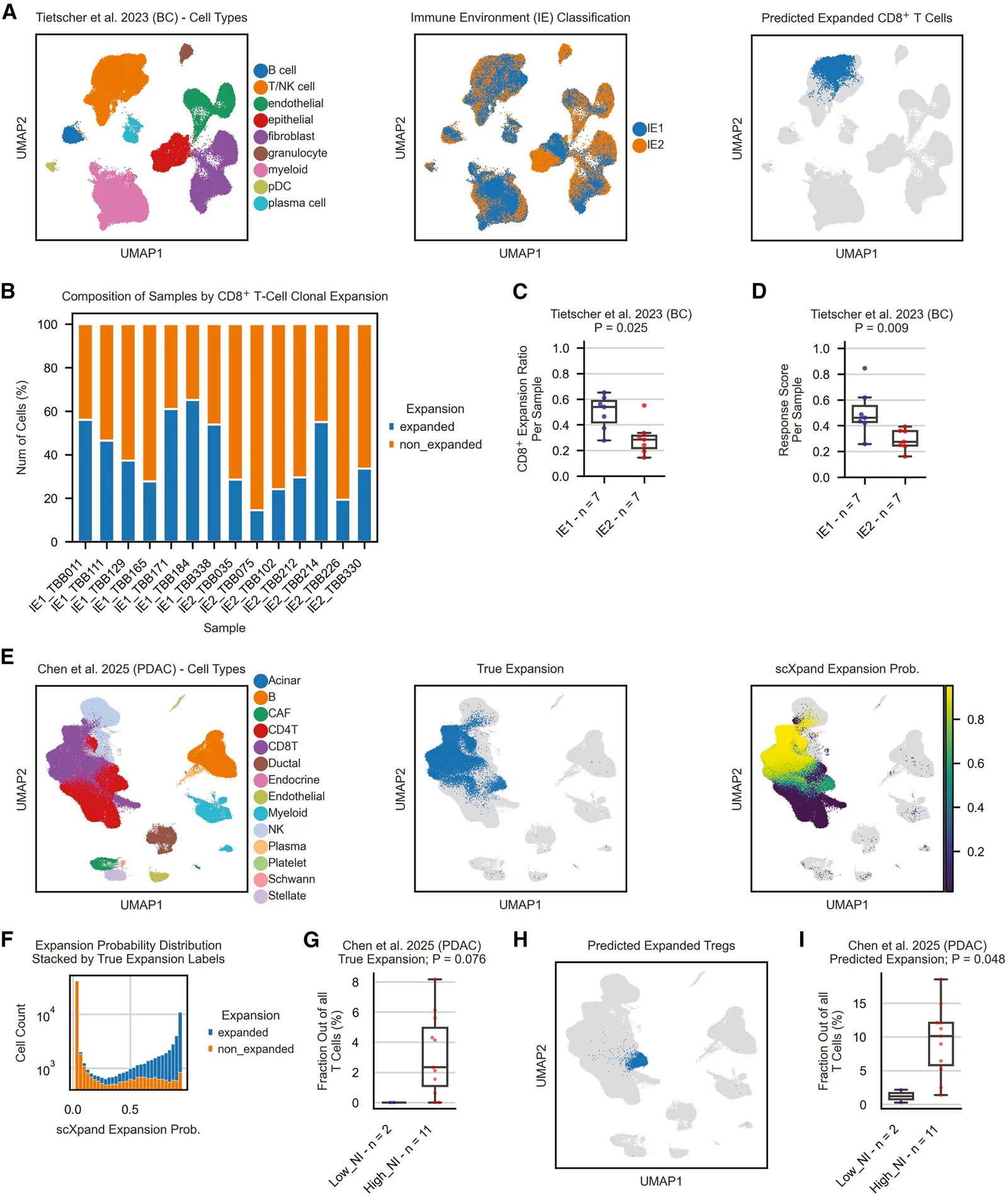
Applicable examples of using the scXpand framework (A) UMAP plots of single cells from a publicly available dataset of patients with breast cancer,^64^ colored by attribution of single cells to cell types and immune environment (IE) classification, as originally provided by the authors,^64^ as well as labeling of CD8^+^ T cells predicted as expanded by the autoencoder model. (B) Stacked barplot showing the fraction of predicted expanded CD8^+^ T cells out of all the CD8^+^ T cells per sample. (C) Boxplot showing the comparison of expanded CD8^+^ T cell fraction per sample, between the different immune environments (*p* = 0.025, *n* = 14). (D) Comparison of a response signature devised by Shorer et al.,^8^ using expanded CD8^+^ T cells, between the different immune environments (*p* = 0.009, *n* = 14). (E) UMAP plots of single cells from a publicly available dataset of patients with PDAC,^65^ colored by attribution of single cells to cell types as originally provided by the authors,^65^ as well as labeling of truly expanded T cells based on scTCR-seq data and the predicted expansion probability of the autoencoder model. (F) A histogram of the predicted expansion probability distribution, stacked by true expansion labels. (G) Boxplot showing the comparison of truly expanded Treg fraction based on scTCR-seq data, out of all T cells per sample, between tumor samples with different cancer-neural invasion status (*p* = 0.076, *n* = 13). (H) UMAP plot of single cells colored by the labeling of intra-tumoral Tregs predicted as expanded by the autoencoder model. (I) Boxplot showing the comparison of predicted expanded Treg fraction by the autoencoder model, out of all T cells per sample, between tumor samples with different cancer-neural invasion status (*p* = 0.048, *n* = 13). BC, breast cancer; PDAC, pancreatic ductal adenocarcinoma; NI, cancer-neural invasion. *p* values were calculated using a two-sided Wilcoxon rank-sum test. See also Figures S26 and S27 and Table S1.

In order to further demonstrate the applicability of our platform, we applied the autoencoder model to a publicly available dataset of patients with breast cancer with scRNA-seq data alone, without paired scTCR-seq data^64^ (Figures 6A and S26A; Table S1; STAR Methods). This dataset consists of 14 patients, with gene expression data of 9 cell types, including T cells (Figure 6A). In addition, patients were originally divided by the authors according to two types of immune environments (IEs), based on the cellular state of T cells: (1) an exhausted environment, IE1, with the presence of PD-1^high^/CTLA-4^high^/CD38^high^ T cells, and (2) non-exhausted environment, IE2, containing T cells that did not express exhaustion markers^64^ (Figure 6A). By applying our model to T cells alone, we identified those with a high probability of being expanded (Figure S26A) and labeled the corresponding CD8^+^ T cells as expanded (Figure 6A; STAR Methods). We then quantified the abundance of expanded CD8^+^ T cells out of all CD8^+^ T cells per sample (Figure 6B) and compared this abundance between the two IEs. Although the overall T cell frequency was previously shown to be similar in both groups,^64^ we found that expanded CD8^+^ T cells are significantly more abundant in IE1 than in IE2 (*p* = 0.025, *n* = 14; Figure 6C), suggesting the potential connection between the IE and T cell clonal expansion. We then used our previously devised 12-gene signature of T cell clonal expansion in the context of patient response to checkpoint immunotherapy^8^ and applied it to expanded CD8^+^ T cells per sample (STAR Methods). We previously used this signature to link clonal expansion to either improved or poor clinical outcomes across multiple cancer types.^8^ Applying this signature to the breast cancer dataset resulted in a score per sample between 0 and 1, quantifying the amount of expanded CD8^+^ T cells associated with good prognosis, out of all the expanded CD8^+^ T cells per sample (Figure 6D). Comparing this score between both IEs showed a significant difference between IE1 and IE2 (*p* = 0.009, *n* = 14; Figure 6D), showcasing that the cellular state of expanded CD8^+^ T cells is also different between the two environments, in addition to the difference in the general abundance of expanded CD8^+^ T cells. Overall, this analysis demonstrates the applicability of our model to a single-cell dataset lacking TCR sequencing and its ability to provide additional valuable information for the downstream data analysis.

To further demonstrate the generalizability of our framework, we applied the autoencoder model to an additional publicly available dataset of patients with pancreatic ductal adenocarcinoma (PDAC) with paired scRNA/TCR-seq^65^ (Figures 6E and S26B; Table S1; STAR Methods). This dataset includes 29 samples from patients with PDAC that passed our quality control, labeled according to their cancer-neural invasion (NI) status as provided by the authors,^65^ including adjacent normal, tumor, and blood samples (Figure S26C). Using its available scTCR-seq data, we measured the performance of the model in predicting T cell clonal expansion (Figures 6E and 6F) and calculated the AUROC values for each sample type (Figure S26D). Our model achieved AUROC values of 0.868, 0.969, and 0.935 across tumor, blood, and adjacent normal samples, respectively (Figure S26D). These results emphasize the generalizability of our framework, as both pancreatic cancer and adjacent normal samples were not considered during the training process. Interestingly, Tregs were observed by the authors to be in higher proportions within tumor samples of patients with a high NI status.^65^ Using the available scTCR-seq data, we managed to show that this finding corresponds to a higher proportion of expanded Tregs out of all the T cells per sample in the high NI group (*p* = 0.076, *n* = 13; Figure 6G). Importantly, by predicting expanded Tregs in tumor samples using our model (Figure 6H; STAR Methods), we managed to reproduce this finding (*p* = 0.048, *n* = 13; Figure 6I). Lastly, we applied our model to an additional scRNA/TCR-seq dataset of non-cancerous colon biopsies obtained from patients across three different groups: (1) patients with checkpoint-induced colitis, (2) patients with ulcerative colitis, and (3) control subjects undergoing elective procedures such as colorectal cancer screening who were found to be healthy^66^ (Figures S27A and S27B; Table S1; STAR Methods). In addition to achieving high overall performance for predicting clonal expansion, scXpand accurately captured the fraction of expanded T cells per sample across all three patient groups (Figure S27C), further supporting the generalizability of our framework and its ability to identify conserved transcriptional signatures of clonal expansion beyond the context of cancer alone. Taken together, these results demonstrate the generalizability of our model across multiple tissue types and T cell subtypes at the pan-cancer level and its applicability to datasets with scRNA-seq data alone, without paired scTCR-seq, while maintaining reliable predictive performance across diverse biological contexts.

## Discussion

The anti-tumor activity of T cell clones has been widely studied in the context of cancer patient response to therapy, using paired scRNA/TCR-seq data. However, to date, there is no existing model for prediction of T cell clonal expansion from scRNA-seq data without paired scTCR-seq data. Our platform bridges this gap by providing a set of five optimized models trained and validated using our in-house-constructed human pan-cancer database of T cells, across both tumor and blood samples. Our platform provides a user-friendly interface that can be easily used for application, training, and optimization of machine-learning models, enabling us to handle millions of single cells in a memory-efficient manner. Validating the performance of our platform using a set of 12 external test datasets, we showed the robustness, accuracy, and generalizability of our results across different datasets and cancer types, regardless of antigenic context or T cell differentiation state. In addition, we showed the most important genes and non-trivial gene-gene interactions affecting model predictions and provided a biological interpretation of these results. We also demonstrated the applicability of our platform to a breast cancer dataset with scRNA-seq data alone, as well as to a PDAC dataset with paired scRNA/TCR-seq data. In addition, we showed the applicability of scXpand beyond the context of cancer by applying our platform to an additional dataset of non-cancerous colon biopsies.

As part of scXpand, we also introduced a masked multi-task, forked count autoencoder that takes advantage of the specific and unique properties of scRNA-seq data, such as their sparsity and count distribution, and used its integrated classification head to detect T cell clonal expansion. Importantly, all five trained and optimized models are publicly available and are ready for direct application. In summary, this study serves as a foundation for future frameworks and additional scalable single-cell classification tasks for the scientific community worldwide, with a special dedication to the cancer research community. Its novelty lies mostly in the prediction task itself, which was not previously tested, as well as the large scale of the curated training and test datasets.

### Limitations of the study

Although our platform was trained on data at the pan-cancer level using 14 cancer types, it excluded other cancers such as pancreatic, ovarian, gastric, cervical, and hematologic malignancies. Indeed, more cancer types can be addressed in future studies as additional scRNA/TCR-seq data become available. However, we did show high performance on unseen cancer types, including HCC^38^^,^^47^ and pancreatic cancer.^65^ In addition, the model was trained using only tumor and blood samples, without other tissue types such as lymph node samples and adjacent normal tissue. Yet, applying our model to the PDAC data by Chen et al.,^65^ we showed that high performance can be achieved on adjacent normal samples as well. Importantly, corresponding to previous studies analyzing different single-cell models in various tasks,^67^^,^^68^ all five models trained within our platform showed relatively comparable overall performance despite varying levels of complexity. However, they differed in their ability to correctly identify either expanded or non-expanded T cells, emphasizing their inherent trade-offs and highlighting the importance of selecting models based on the specific biological question or application. Moreover, the more complex models, such as LightGBM, capture intricate gene-gene interactions, providing non-linear trends between gene expression and model predictions. The choice of model therefore depends on the user’s objective. Users prioritizing maximal predictive performance may prefer the autoencoder. This will also be the case for users who want to obtain a reconstructed and denoised gene expression matrix, as well as a smoother two-dimensional representation of their data, using the latent space embedding of the autoencoder. In contrast, users interested in biological interpretation, hypothesis generation, or additional insights may prefer the LightGBM model. In addition, users who prioritize memory-light-weight models might prefer to use the provided linear models: SVM and logistic regression. Finally, it is important to note that our platform does not address the actual clonal identity of T cells or their TCR sequence identity; thus, it is limited to detecting only the cellular state of T cell clonal expansion based on gene expression.

Importantly, scXpand outperforms classification based on T cell differentiation states and existing tumor-reactivity signatures.^57^^,^^58^^,^^62^^,^^63^ However, limitations remain in specific cell populations, such as exhausted and terminally exhausted T cells, where predictive performance is comparatively reduced, potentially due to sampling limitations that obscure the full clonal landscape in these populations. Nonetheless, our platform successfully links scRNA-seq data to clonal expansion, providing a scalable framework for this task. It further has the potential to be applied in contexts where scTCR-seq is not yet commercialized or broadly applicable, such as spatial transcriptomics data, thus linking T cell clonal expansion to the spatial location of cells in the tissue. This will be particularly valuable once the current technical limitations of these data are addressed.

## Resource availability

### Lead contact

Further information and requests for resources should be directed to and will be fulfilled by the lead contact, Keren Yizhak (kyizhak@technion.ac.il).

### Materials availability

This study did not generate new unique reagents.

### Data and code availability

• This paper analyzes existing, publicly available scRNA/TCR-seq data. The accession numbers for these datasets are summarized in Table S1.
• All original code has been deposited at https://github.com/yizhak-lab-ccg/scXpand and is publicly available as of the date of publication. The complete set of trained models is publicly accessible via Figshare^69^ (https://doi.org/10.6084/m9.figshare.30067666). The full documentation of our platform, including tutorials and guidelines, is available on Read the Docs (https://scxpand.readthedocs.io). An official Python package is provided via PyPi (https://pypi.org/project/scxpand), with an additional GPU-supported version (https://pypi.org/project/scxpand-cuda).
• Any additional information required to reanalyze the data reported in this paper is available from the lead contact upon request.

## Acknowledgments

We would like to thank Asaf Pinhasi for fruitful discussions and his helpful comments. This work was supported by the 10.13039/501100003977Israel Science Foundation (1124/25), the Israel Cancer Research Fund (23-204-RCDA), and the Career Advancement Chairs in Artificial Intelligence – Schmidt Futures. This work received additional support from the Ruth and Bruce Rappaport Technion Integrated Cancer Center (RTICC). Figures 1A, 2D, 3A, 4A, 5A, and S2, as well as the graphical abstract, were created with BioRender.com using a paid license.

## Author contributions

O.S., R.A., and K.Y. conceived the idea. O.S., R.A., and K.Y. designed the study. O.S. and R.A. performed the analysis. O.S., R.A., and K.Y. wrote the manuscript.

## Declaration of interests

The authors declare no competing interests.

## STAR★Methods

### Key resources table

**Table:** 

REAGENT or RESOURCE	SOURCE	IDENTIFIER
**Deposited data**		

scRNA/TCR-seq data – RCC	Au et al.^14^	https://doi.org/10.5522/04/16573640.v1
scRNA/TCR-seq data – NSCLC	Caushi et al.^15^	GEO: GSE176021
scRNA/TCR-seq data – RCC	Krishna et al.^16^	https://trace.ncbi.nlm.nih.gov/Traces/sra/sra.cgi?analysis=SRZ190804
scRNA/TCR-seq data – NSCLC	Liu et al.^6^	GEO: GSE179994
scRNA/TCR-seq data – HNSCC	Luoma et al.^17^	GEO: GSE200996
scRNA/TCR-seq data – NSCLC	Pai et al.^18^	GEO: GSE185206
scRNA/TCR-seq data – TNBC	Shiao et al.^19^	GEO: GSE246613
scRNA/TCR-seq data – BCC	Yost et al.^5^	GEO: GSE123813
scRNA/TCR-seq data – SCC	Yost et al.^5^	GEO: GSE123813
scRNA/TCR-seq data – TNBC	Zhang et al.^20^	GEO: GSE169246
scRNA/TCR-seq data – BC – Two cohorts	Bassez et al.^21^	https://lambrechtslab.sites.vib.be/en/single-cell
scRNA/TCR-seq data – Melanoma	Barras et al.^22^	GEO: GSE222448
scRNA/TCR-seq data – Glioblastoma	Bagley et al.^23^	GEO: GSE242790
scRNA/TCR-seq data – MCC	Reinstein et al.^24^	GEO: GSE235093
scRNA/TCR-seq data – Melanoma	Cillo et al.^25^	GEO: GSE225063
scRNA/TCR-seq data – NSCLC	Zhang et al.^26^	GEO: GSE241934
scRNA/TCR-seq data – Endometrial	Eerkens et al.^27^	GEO: GSE268903
scRNA/TCR-seq data – HNSCC	Cha et al.^28^	GEO: GSE226620
scRNA/TCR-seq data – RCC	Borcherding et al.^29^	GEO: GSE121638
scRNA/TCR-seq data – NPC	Gong et al.^30^	GEO: GSE150825
scRNA/TCR-seq data – CRC	Chen et al.^31^	GEO: GSE236581
scRNA/TCR-seq data – NPC	Liu et al.^32^	GEO: GSE162025
scRNA/TCR-seq data – HNSCC	Rahim et al.^33^	GEO: GSE212797
scRNA/TCR-seq data – Endometrial	Chow et al.^34^	GEO: GSE212217
scRNA/TCR-seq data – Bladder/Prostate	Gu et al.^35^	GEO: GSE266079
scRNA/TCR-seq data – Brain	Wang et al.^36^	https://zenodo.org/records/10672442
scRNA/TCR-seq data – CRC	Liu et al.^37^	GEO: GSE164522
scRNA/TCR-seq data – HCC	Guo et al.^38^	GEO: GSE235863
scRNA/TCR-seq data – Melanoma	Wang et al.^39^	GEO: GSE272734
scRNA/TCR-seq data – TNBC	Zhang et al.^40^	GEO: GSE266919
scRNA/TCR-seq data – ESCC	Zheng et al.^41^	GEO: GSE145370
scRNA/TCR-seq data – BC	Liu et al.^42^	GEO: GSE167036
scRNA/TCR-seq data – Glioma	Ogino et al.^43^	GEO: GSE188620
scRNA/TCR-seq data – NSCLC	Liu et al.^44^	GEO: GSE243013
scRNA/TCR-seq data – NSCLC	Baek et al.^45^	GEO: GSE274934
scRNA/TCR-seq data – NSCLC	Liu et al.^46^	GEO: GSE190905
scRNA/TCR-seq data – HCC	Shu et al.^47^	GEO: GSE272347
scRNA/TCR-seq data – PDAC	Chen et al.^65^	GEO: GSE278694
scRNA/TCR-seq data – Colon biopsies	He et al.^66^	GEO: GSE253720
scRNA-seq data – BC	Tietscher et al.^64^	ArrayExpress: E-MTAB-10607

**Software and algorithms**		

scXpand	This paper	https://github.com/yizhak-lab-ccg/scXpand
Scanpy	Wolf et al.^70^	https://github.com/scverse/scanpy
Scirpy	Sturm et al.^71^	https://github.com/scverse/scirpy
Scrublet	Wolock et al.^72^	https://github.com/swolock/scrublet
Markov Affinity-based Graph Imputation of Cells (MAGIC)	Van Dijk et al.^48^	https://github.com/KrishnaswamyLab/MAGIC
Batch Balanced K-Nearest Neighbors (BBKNN)	Polański et al.^73^	https://github.com/Teichlab/bbknn
Umap-learn	McInnes et al.^74^	https://github.com/lmcinnes/umap
LightGBM	Ke et al.^50^	https://github.com/lightgbm-org/LightGBM
Optuna	Akiba et al.^75^	https://github.com/optuna/optuna
SHAP	Lundberg et al.^59^	https://github.com/shap/shap
Pytorch	Paszke et al.^76^	https://github.com/pytorch/pytorch
Scikit-learn	Pedregosa et al.^77^	https://github.com/scikit-learn/scikit-learn
Pooch	Uieda et al.^78^	https://github.com/fatiando/pooch
GSEApy	Fang et al.^61^	https://github.com/zqfang/GSEApy
Adobe Illustrator	Adobe	https://www.adobe.com/products/illustrator.html
Cursor	Anysphere, Inc.	https://cursor.com/
BioRender	BioRender	https://www.biorender.com/

### Experimental model and study participant details

This study did not involve any new experimental models, human participants, or animal subjects. All data used in this study are publicly available, with samples and single cells obtained from each dataset described in Table S1 following our quality control. Details regarding data acquisition and processing are provided in the method details.

### Method details

#### Acquisition of paired scRNA/TCR-seq datasets and preprocessing

26 single-cell datasets of cancer patients having paired scRNA/TCR-seq data were collected together with their annotations for clinical outcomes and treatment time-point, where available. 15 datasets contained tumor biopsies, 9 datasets contained both tumor and blood samples, and 2 datasets contained blood samples alone (Table S1).

All scRNA-seq datasets were droplet-based and contained unique molecular identifier (UMI) counts. For each dataset, we followed similar preprocessing steps to those we previously conducted^8^ using Scanpy^70^: We removed cells expressing less than 200 genes and removed genes that were expressed in less than 3 cells. We also removed cells having more than 10% of mitochondrial gene-count. In the dataset of Krishna et al.,^16^ scRNA-seq was provided following the authors’ original quality-control (QC) while excluding mitochondrial genes from the provided gene expression matrix. In that case only, we used the expression matrix as provided by the authors following their QC, retaining cells with less than 20% of mitochondrial gene-count. We also applied Scrublet^72^ to remove potential doublets and filtered-out cells having *‘doublet_score’* larger than 0.3 and/or those predicted as doublets with score below 0.3.

For the scTCR-seq datasets, we rearranged each dataset to be compatible with the Adaptive Immune Receptor Repertoire (AIRR) schema^79^^,^^80^ that was further preprocessed by Scirpy^71^ and could be directly uploaded using the *‘scirpy.io.read_10x_vdj’* function. For each scTCR-seq dataset, we applied quality control based on the standard protocol suggested by the authors. In short, we removed cells having multi-chains, orphan VJ or orphan VDJ chains.

#### Integration of scRNA/TCR-seq datasets

Following the quality control process described above, we removed samples having less than 3 cells overall, and integrated all processed datasets resulting with a total of 1,055,922 single cells from tumor samples and 532,387 single cells from blood samples, all with paired scRNA/TCR-seq. Overall, 11,950 genes existed across all datasets and were used for further analysis, such that tumor and blood samples were visualized separately.

For visualization purposes only (Figures 1B, 1C, S1A–S1D, and S6A), we normalized the expression level in the integrated scRNA-seq datasets to a standard target sum of 10,000 counts per cell and then applied log-transformation. Highly variable genes were calculated using *‘scanpy.pp.highly_variable_genes’* with a *‘batch_key’* of *‘cancer type’* in order to avoid selection of cancer-type-specific genes, ensuring better downstream batch correction. We then calculated a 40-component PCA and applied Batch Balanced K-Nearest Neighbors (BBKNN)^73^ with a ‘*batch_key’* of *‘sample’* in order to remove batch effects between samples. UMAP^74^ was used for dimensionality reduction and data visualization.

#### Markov affinity-based graph imputation of cells (MAGIC) for detection of dropouts and labeling of expanded clones

In order to identify possible dropouts in our integrated datasets, we applied MAGIC^48^ to the log-normalized count matrices of tumor and blood samples separately, using all the genes and single cells passing our QC, while using the default arguments of the algorithm implementation in Scanpy.^70^ We first fitted a density curve to the log-transformed imputed gene expression of *CD8A/B*, *CD4*, and *FOXP3*, and set the expression threshold for each gene as the trough of the bimodal density curve (Figures S1A and S1B). For the imputed gene expression of *FOXP3*, the threshold was set as 1. For the non-imputed expression of these genes, log-transformed expression threshold was also set as 1. Overall, all the T cells that passed our QC process were further divided into five subtypes (Figures S1C and S1D): we considered a single cell to be CD8^+^, when the imputed or non-imputed gene expression of either *CD8A* or *CD8B* was above the threshold. Similarly, a single cell was considered to be CD4^+^, when the imputed or non-imputed gene expression of *CD4* was above the threshold. We also defined double-positive cells as those that were considered to be both CD8^+^ and CD4^+^. Double-negative cells were those that were both CD8^−^ and CD4^−^. Treg cells were defined as those that are CD4^+^ having imputed or non-imputed gene expression of *FOXP3* above the threshold. In cases where the imputed expression resulted with loss of signal from the non-imputed gene expression, we remained consistent with the non-imputed gene expression. Importantly, the imputed gene expression was used solely for cell type classification, while the model input itself included non-imputed gene expression to retain the original data structure and prevent potential artifacts introduced by imputation.

We then defined clonotypes based on the identity of the CDR3 nucleic acid sequence of each cell using *‘scirpy.tl.define_clonotypes’*, such that both the VJ and VDJ CDR3 sequences had to match and the T cell subtype of all the cells in each clone is the same (i.e., CD8^+^, CD4^+^, double-positive, double-negative or Treg). In cases where more than one pair of VJ and VDJ sequences was detected per cell, we considered the most abundant pair of VJ/VDJ chains where applicable. In the dataset of Krishna et al.,^16^ scTCR-seq was provided only with the amino acid sequence of the CDR3 region. In that case only, clonotypes were defined based on the amino acid identity of the CDR3 sequence. Of note, an in-house evaluation using a more conservative clonotype definition, requiring both identical CDR3 sequences and identical V genes (using the argument ‘*same_v_gene = True*’), resulted with 99.78% concordance of clonotype identity compared to using CDR3 sequences alone. The same concordance was achieved by requiring identical J genes (using the argument ‘*same_j_gene = True*’), in addition to identical CDR3 sequences and identical V genes. To ensure our framework is robust and compatible with studies reporting only CDR3 sequences, we based clonotype definitions solely on CDR3 identity.

We defined a clonotype as ‘expanded’ per sample according to Shiao et al.,^19^ and as we did previously,^8^ such that ‘expanded clones’ were those that contained more than 1.5x the median number of cells found in each clonotype in that sample, while excluding singletons. This approach normalizes for differences in sampling depth and repertoire complexity between samples and avoids the biases introduced by fixed-clone-size thresholds, enabling consistent identification of clonal expansion across datasets. This relatively conservative threshold reduces the impact of noise while capturing clones with potential functional impact. Importantly, our framework enables flexible modification of this threshold during training according to the user preferences.

In order to conduct further analysis at the level of each sample, we assigned each clone with a unique ID such that the same clone appearing in multiple samples from the same patient received a different ID, indicating the clone sample of origin. We then used VDJdb^55^ as a reference database for annotating epitopes based on amino acid sequence identity according to the standard protocol suggested by the authors^71^ (Figure S6A).

#### Creating the scXpand framework

We created a framework supporting multiple model architectures, consisting of five different models. These include two linear models (SVM and logistic regression), one tree-based model (LightGBM), and two deep-learning models that include a multilayer perceptron (MLP) and a multi-task autoencoder. Both linear models of SVM and logistic regression were trained using the Scikit-learn implementation of stochastic gradient descent classifier (‘*sklearn.linear_model.SGDClassifier’*),^77^^,^^81^ enabling large-scale learning with efficiency and ease of implementation. The tree-based LightGBM gradient boosting model,^50^ supports fast training and high efficiency, with relatively low memory usage, enabling it to handle large-scale data. It was trained using its built-in implementation in Python (‘*lightgbm.LGBMClassifier’*). As for the two deep-learning models, we used the PyTorch library^76^ in order to define the architecture of each model. In short, the MLP model includes fully connected layers, incorporating dropout regularization to mitigate overfitting, with an optimization handled using the AdamW optimizer,^82^ while a scheduler selected from multiple possible options dynamically adjusts the learning rate (Table S2). The autoencoder-based model is an extension of the deep count autoencoder (DCA) model^51^ to the supervised learning setting. The original DCA model is composed of a decoder and encoder and is trained with only gene counts in an unsupervised manner, in order to learn a meaningful lower dimensional embedding of the data. The decoder results with the parameters of the zero inflated negative binomial model (ZINB): mean, dispersion, and dropout probability parameter. We extended this model by adding another classification head module that predicts the expansion state based on the latent vector (Figure S2). We also implemented two possible configurations of standard and forked architecture. The forked autoencoder extends the standard design by using separate decoder branches for the mean, dispersion, and dropout probability parameters, enabling more flexible modeling of complex data distributions. In addition to the integrated classification head for the main task of predicting T cell clonal expansion, this model supports additional possible auxiliary classification tasks, such as identification of the tissue type of origin for each T cell (i.e., tumor or blood), identification of T cell subtype (i.e., CD8^+^, CD4^+^, Treg, double-negative or double-positive), or both. Importantly, this model enables multiple different reconstruction loss functions, including negative binomial, zero-inflated negative binomial, or MSE. All of the modules are jointly trained end-to-end with a loss function that adds the classification losses and the reconstruction loss.

The final architecture of each model, including the number of hidden layers, layer dimensions, the existence and structure of the auxiliary classification heads, as well as the hyperparameter tuning for all of the five different models, were determined following an optimization process using Optuna,^75^ with 100 trials for each model (Figure S3). The full lists of hyperparameters for each model appear in Table S2, with the readily trained and optimized models that are publicly accessible.

#### Model training

Each one of the different five models was trained on a set of more than 1.5 million T cells, following a structured workflow, beginning with data preprocessing and a stratified splitting into training (80%) and validation (20%) sets. Data stratification ensured similar distribution of tissue types (blood or tumor) and cancer types between the train and validation sets. We also restricted all of the samples obtained from a single patient to be used in either train or validation, but not both, ensuring that data from the same patient cannot appear in both sets. Preprocessing included count normalization to a target sum of 10,000 counts per cell, including an optional log-transformation (if specified), and followed by *Z* score normalization (if specified), where the empirical mean and standard deviation per gene were calculated using only the training set. During training, we used the following two data augmentation methods: to improve robustness to sequencing dropouts, we added random masking to the gene expression matrix, randomly zeroing small subset of the gene counts and added random white Gaussian noise to increase robustness for measurement errors. Hyperparameter tuning for each model was conducted using Optuna,^75^ which explores parameter spaces across multiple trials, enabling efficient trial pruning during model optimization. The pipeline integrates automated logging, checkpointing, and systematic model evaluation to ensure reproducibility. Model inference is executed with batched evaluation on the validation data, and performance is assessed using comprehensive metrics evaluated for different pairs of tissue-type (blood or tumor) and T cell subtypes (CD8^+^, CD4^+^, double-negative, double-positive, and Treg). Optimal configuration of hyperparameters was selected for each model based on the harmonic average of the AUCs across all possible ‘tissue type-T-cell subtype’ combinations. This was done in order to ensure the model performs well across all possible T cell subtypes from the different tissue types, as well as giving higher weights to low AUC values using the harmonic average, compared to the standard arithmetic mean. Once trained, the model is saved for further downstream analyses, ensuring its applicability to external scRNA-seq datasets. Importantly, the model is compatible with Scanpy’s standard data structure^70^ and was optionally trained on soft labels for T cell expansion in the range of 0–1, defined by the ratio between the clone size and 1.5x the median number of cells found in each clonotype per sample. The optimized models were made publicly available with support for automatic model download using Pooch.^78^ Hardware used for the training of both deep-learning models and the LightGBM model, included a workstation equipped with an Intel core Ultra 9 285K CPU 256 GB RAM and an NVIDIA RTX 6000 Ada Generation GPU 48 GB VRAM. Both linear models (logistic regression and SVM) were trained using a workstation equipped with an Intel core i9-14900KF CPU 128 GB RAM and an NVIDIA GeForce RTX 4080 SUPER GPU 16 GB VRAM.

#### Comparison between the different trained models

Across all 100 trials per model, we selected the best trial based on the harmonic average of the AUCs across all possible ‘tissue type-T-cell subtype’ pairs, as described above (Figure S3). We then compared the performance of each model on the validation set using a set of evaluation metrics, such that a T cell was predicted as ‘expanded’ using a naive threshold of 0.5 for the expansion probability obtained by the model. These metrics include accuracy, precision, recall, specificity, F1 score, area under the ROC curve, area under the precision-recall curve, as well as the Matthews correlation coefficient (MCC),^53^^,^^54^ which considers the imbalance of labels in our data (Figure 2; Table S2). The MCC distribution by the predicted expansion probability was then calculated for each model using all possible ‘tissue type-T-cell subtype’ combinations (Figure S4), including identification of optimal probability threshold for each combination in each model. We also evaluated model performance separately for T cells targeting known viral antigens and for T cells that do not target any known non-cancerous antigens (potentially cancer-specific T cells) from the validation set, based on their epitope identity (Figure S6). This included a differential gene expression analysis performed between expanded T cells targeting viral and non-viral antigens across the entire training dataset (Figure S7A), as well as between expanded and non-expanded T cells targeting viral antigens alone (Figures S7B and S7C). Of note, a permutation test where we retrained the models from scratch using randomly permuted expansion labels, resulted in near-random performance, indicating that the observed predictive performance cannot be explained by exploitation of global data distribution alone.

We also re-labeled clonal expansion using patient-level labeling, compared to our original sample-level labeling, by grouping T cells sharing identical CDR3 sequences across all the samples obtained from the same patient when computing clone size (Figure S5A). We then re-trained the autoencoder model using the patient-level labeling of clonal expansion and evaluated its predictions compared to those obtained from the model trained using the sample-level labeling. Following, we used the validation set to evaluate the model trained using patient-level labeling, across expansion labels obtained using the sample-level approach, and also evaluated our original model trained using sample-level labeling, across expansion labels obtained using the patient-level approach. We then evaluated the area under the corresponding ROC curves for tumor and blood samples separately, and calculated the Spearman correlation between the prediction probabilities obtained by both models across T cells from the validation set, using Spearman’s rank correlation test (Figures S5B and S5C).

#### scXpand implementation on test datasets

In order to test the performance of each model in an unbiased manner, we used additional 12 datasets containing more than 1 million T cells from 482 samples (Table S1). All of these datasets were entirely external to the model and were not used during the training and optimization procedures. For each dataset, we applied a separate quality control on each of the two data modalities, scRNA-seq and scTCR-seq, as described above, and labeled the expansion status of each T cell accordingly. We then applied each model using the raw UMI count matrix of each dataset, obtaining an expansion probability for every single T cell (Figures 3 and S8–S10; Table S3). In cases where genes that were used during training were missing in the test dataset (Table S3), they were automatically added to the relevant dataset with a gene expression of zero, mimicking potential sequencing dropouts, and were passed with the formatted gene expression matrix as an input to the model. For each model we calculated the area under the ROC curve across all datasets and visualized the distribution of the expansion probability, stacked by the true expansion status. We used UMAP for visualization purposes, as described above.

Model performance was also evaluated across the test datasets, while stratified by different T cell subtypes in both tumor and blood samples (Table S3). The performance achieved for CD8^+^ and CD4^+^ T cells was then compared between the already trained autoencoder and two specific autoencoder models trained separately using either CD8^+^ T cells or CD4^+^ T cells alone (Figure S13). The Spearman correlation of the mean expansion probability per clone was then compared within each T cell subtype between the already trained non-specific autoencoder (i.e., the model trained using all of the different T cell subtypes together) and the specific models trained using a specific T cell subtype alone (Figure S14, *p*-values were calculated using Spearman’s rank correlation test). Similar process was performed to evaluate model performance while stratified by tumor and blood samples, regardless of any T cell subtype, and compare its performance to that achieved using two specific autoencoder models trained separately using either tumor or blood samples alone (Figure S15).

#### Training subsampling test and expansion threshold sensitivity test

To assess the benefit of leveraging a scalable, large-scale database for training models to detect clonal expansion, we selected different fractions of the training data, including 5%, 10%, 25%, 50%, and 75%, and retrained the autoencoder model from scratch for each fraction, using the subsampled training dataset. Model performance was then assessed on the validation set and across the twelve test datasets (Figures S11A and S11B). We used a two-sided Wilcoxon signed-rank test to compare the AUROCs achieved for the test datasets, between a model trained on the full training set and a model trained using 75% of the data (Figure S11B).

To further justify the selection of our expansion threshold, as indicated above, we performed a sensitivity test showing how it changes by different thresholds of clonal expansion, including the fraction of T cells labeled as expanded for each threshold, out of all the 1.5m T cells found in the training set (Figure S11C). Moreover, we also trained the autoencoder from scratch using additional different thresholds for clonal expansion (focusing on 2x and 1x thresholds, in addition to the 1.5x threshold used throughout our analysis), followed by calculation of the area under the ROC curve and visualization of the distribution of the expansion probability, stacked by the true expansion status determined by each threshold (Figures S11D and S11G). We then used confusion matrices to demonstrate the fraction of T cells labeled as ‘expanded’ or ‘non-expanded’ across the validation set, using different expansion thresholds (Figures S11E and S11H). Lastly, we used scatterplots to demonstrate the differences in expansion probability for each T cell in the validation set, as predicted by models trained using different expansion thresholds (Figures S11F and S11I).

We performed an additional threshold comparative analysis using the validation set, further quantifying the amount of T cells and T cell clones labeled as ‘expanded’ across different expansion thresholds (Figures S12A and S12B). We applied the different models that were trained using the 1.5x threshold, and measured their AUROCs when applied to different expansion thresholds ranging from 0.5x to 3x (Figure S12C). We also calculated the correlation of the mean expansion probability per clone obtained using these models, with clone size, using Spearman’s rank correlation test (Figure S12D). Finally, we re-trained all of the five different models using an expansion threshold of 1x and 2x, and evaluated their AUROCs across the validation set (Figure S12E).

#### Model explainability using SHAP

In order to provide a biological interpretation of the model predictions, we used the SHapley Additive exPlanations (SHAP) values^59^^,^^60^ for the validation set. We used the implementation of the SHAP Python library with default parameters, and applied it to the LightGBM model. We used the ‘*shap.summary_plot*’ function in order to visualize the 20 most important features driving the model predictions, together with their directionality and magnitude of their impact (Figure 4B and Table S2). We then used the ‘*shap.dependence_plot*’ function to show the effect of single genes across the entire validation set, accounting for possible interaction effects with other genes (Figures 4D–4F). We did this process using all the cells in the validation set, as well as for CD8^+^ and CD4^+^ T cells from tumor samples separately (Figures S16A and S16B). It is important to note that the same exact LightGBM model was used for SHAP value computation for the validation set, as well as for CD4^+^ and CD8^+^ T cells from tumor samples. We repeated this process for the logistic regression model using all the cells from the validation set to demonstrate differences of gene-gene interactions between the simple logistic regression model and the more complex tree-based LightGBM model (Figures S18A and S18B). Specifically for the LightGBM model, we also used its built-in ‘*feature_importance*’ function with ‘*importance_type = gain*’ to reflect the overall contribution of each gene to the predictive performance of the model (Figure 4C).

Pathway enrichment analysis was performed using the GSEApy ‘enrichr’ module,^61^ applied to the top explainable genes obtained using the SHAP analysis for expanded and non-expanded T cells from the validation set. More explicitly, for both T cell groups, we ranked a list of the top explainable genes based on the mean absolute SHAP values of each gene, and evaluated the Spearman correlation between gene expression and the SHAP values for each gene using Spearman’s rank correlation test. We then retained genes having adjusted Spearman *p*-values <0.05 following correction for multiple hypothesis, using the Benjamini-Hochberg false discovery rate^83^ (Table S2). We then used the ‘*Reactome_Pathways_2024*’ gene sets defined by the Reactome Pathway Knowledgebase,^84^ providing manually curated molecular details of a broad range of human biological processes. We applied ‘enrichr’ for expanded and non-expanded T cells separately and considered a significance cutoff of 0.05 for the adjusted *p*-values (Figure S17 and Table S2).

#### Evaluating scXpand across different T cell states and comparison with published gene signatures

In order to demonstrate the added value of scXpand over classic markers of T cell differentiation states, and to show that our platform successfully functions as a true clonal expansion predictor over a basic T cell differentiation classifier, we focused on 5 test datasets (out of the 12 evaluated test datasets), where the authors provided publicly available annotations for T cell differentiation states^37^^,^^38^^,^^40^^,^^42^^,^^44^ (Figure 5). Focusing on CD8^+^ T cells, we resulted with 336,363 cells overall across all 5 datasets. Using the optimized autoencoder model, we measured its prediction performance across three T cell states separately, including naive/memory, effector/effector-memory, and exhausted/terminally exhausted T cells. We calculated the area under the ROC curve for each group and visualized the distribution of the expansion probability, stacked by the true expansion status (Figure 5E). We then focused only on expanded T cells per group, and calculated differentially expressed genes (DEGs) per group using the ‘*scanpy.tl.rank_genes_groups*’ function with the Wilcoxon rank-sum test (Figure 5G). We made an additional classification of T cells, labeling them as singletons if their clone size is one, and non-singletons otherwise. We calculated the fraction of both groups in each T cell state (Figure S21A), and measured the performance of the model for separating singletons and non-singletons in each group (Figure S21B). We further calculated DEGs for expanded and non-expanded T cells, focusing separately on naive/memory and exhausted T cell states (Figures S22B and S22C). Differentially expressed genes presented for exhausted T cells, were those having an adjusted *p*-value lower than 0.05, log-fold change higher than 0.5, and non-zero expression in at least 15% of either expanded or non-expanded T cells. For the naive/memory state, the same criteria were applied, while considering log-fold change higher than 1. We additionally performed differential gene expression within the exhausted T cell state, between T cells predicted as expanded and non-expanded by the model (Figure S22D), and visualized the mean predicted expansion probability of T cells per clone for each T cell state, according to the clone size of each clone (Figure S22E). For each T cell state individually, we also performed a separate SHAP analysis as described above, and ranked the top 100 explainable genes for each differentiation state, while calculating the Spearman correlation between the gene expression and the SHAP values across these genes, using Spearman’s rank correlation test (Figure S20 and Table S3).

The performance of our model was then compared to that of a published gene signature for neoantigen-reactive tumor-infiltrating CD8^+^ T cells.^58^ We used the 243-gene version of the ‘NeoTCR8’ signature provided by the authors, and scored each CD8^+^ T cell per group using the ‘UCell’ package implementation in Python (pyUCell).^85^ We calculated the area under the ROC curve for each T cell differentiation state and visualized the distribution of the score, stacked by the true expansion status (Figures S23A and S23H). Similar scoring process was done for two additional tumor-reactivity signatures, including the ‘CD8 Transcriptome Signature’ by Hanada et al.^63^ (Figures S23D and S23F) and the ‘TR30’ signature by Meng et al.^62^ (Figures S23C and S23G). Specifically for the signature by Hanada et al.,^63^ the overall score of the signature per cell was calculated as the score of the genes that positively contributed to the signature, subtracted by the score of the genes that negatively contributed to the signature. We also calculated the ‘MANAscore’ of each CD8^+^ T cell by running the original pipeline provided by the authors (https://github.com/BKI-immuno-KNS/MANAscore), and used their pre-trained models to calculate the ‘MANAscore’ for both imputed (‘*MANAscore_i*’) and non-imputed (‘*MANAscore_ni*’) gene expression^57^ (Figures S23B and S23E). Similar to the authors, we calculated the overall ‘MANAscore’ combining both ‘*MANAscore_i*’ and ‘*MANAscore_ni*’ into one score ($\sqrt{{MANAscore\_i}^{2}+{MANAscore\_ni}^{2}\;}$), and calculated the area under the ROC curve for each group. Specifically, the ‘MANAscore’ was also tested separately for both viral- and non-viral-specific T cells (Figures S24A and S24B). In addition, we constructed two streamlined gene signatures composed of the top 5 genes obtained by our SHAP analysis (Figure 4B), resulting with one signature associated with clonal expansion (*NKG7*, *CD8A*, *GZMA*, *CST7* and *CXCL13*) and another signature associated with non-expansion (*LTB*, *SELL*, *CCR7*, *LEF1* and *MAL*). We used these signatures to score each CD8^+^ T cell per group using the ‘*scanpy.tl.score_genes*’ function and calculated the area under the ROC curve for each group (Figures S25A–S25C). Lastly, using confusion matrices, we showed that our model predictions for clonal expansion better separate for true clonal expansion status, compared to the separation of clonal expansion achieved by T cell differentiation states (Figure 5H). That is, a calculation of the fraction of truly expanded and non-expanded T cells across T cell states, and also across the predicted expansion status, separately.

#### Application of the autoencoder to scRNA-seq dataset of breast cancer patients without paired scTCR-seq

We utilized a publicly available scRNA-seq dataset of 14 breast cancer patients, including cell-type classification and patient stratification by immune environments, as originally provided by the authors^64^ (Figures 6A and S26A; Table S1). We applied a strict quality control using the gene expression matrix as described above, including filtration of cells, genes, doublet removal, followed by extraction of highly variable genes and visualization using UMAP. MAGIC^48^ was applied to detect sequencing dropouts as described above, followed by classification of T cell subtypes. We applied our autoencoder model using only cells classified as T cells by the authors, and detected expanded CD8^+^ T cells using the relevant optimal autoencoder threshold for CD8^+^ T cells in tumor samples (Figure S4A). We then calculated the fraction of expanded CD8^+^ T cells out of all the CD8^+^ T cells per sample and used a two-sided Wilcoxon rank-sum test to compare these ratios between patients based on their immune environment classification (Figures 6B and 6C). Focusing on expanded CD8^+^ T cells alone, we applied our previously devised response score of expanded CD8^+^ T cells in the context of patient response to checkpoint immunotherapy,^8^ and calculated a response score per patient. In short, we used a twelve gene signature, including *CXCL13*, *DUSP4*, *RBPJ*, *LYST*, *GZMK* and *HLA-DQA1*, accounting for good prognosis, as well as *FOS*, *ZNF683*, *CTSC*, *GZMH*, *ANXA1* and *XCL2*, accounting for poor clinical outcomes. We then used these two lists of genes and scored each expanded CD8^+^ T cell based on the number of expressed genes (*log*(*normalized*_*counts*+1)>1) out of the 6 markers in each list, yielding two scores for each expanded CD8^+^ T cell. Then, each CD8^+^ T cell was classified as ‘favorable’ or ‘unfavorable’ based on the dominant score. In cases where both scores were tied in a single cell, it was classified as ‘favorable’. Finally, we computed a score per patient by taking the ratio between the number of cells classified as ‘favorable’ out of the total number of cells classified as ‘favorable’ or ‘unfavorable’, yielding a score per patient between 0 and 1. CD8^+^ T cells demonstrating an expression of zero markers from both lists of markers were excluded from this analysis. We then used a two-sided Wilcoxon rank-sum test to compare these scores between patients based on their immune environment classification (Figure 6D).

#### Application of the autoencoder to scRNA/TCR-seq dataset of PDAC patients

We utilized a publicly available scRNA/TCR-seq dataset of PDAC patients, including cell-type classification and patient stratification by cancer-neural invasion (NI) status, as originally provided by the authors^65^ (Figures 6E and S26B; Table S1). We applied a strict quality control using both the gene expression matrix and the scTCR-seq data as described above, including labeling of expanded T cells with a specific focus on expanded Tregs. We further applied our autoencoder model to cells having TCR sequence passing our quality control, and obtained their expansion probability. The area under the ROC curve was then calculated separately by the different sample types: adjacent normal, blood, and tumor samples (Figures S26C and S26D). Using optimal Treg expansion threshold in tumor samples for the autoencoder model (Figure S4A), we labeled Tregs predicted as expanded by the model and calculated the fraction of both truly expanded and predicted expanded Tregs out of all the T cells per sample (Figures 6G–6I). We used a two-sided Wilcoxon rank-sum test to compare these fractions between patients based on their cancer-neural invasion status.

#### Application of the autoencoder to dataset of non-cancerous colon biopsies

We utilized a publicly available dataset of non-cancerous colon biopsies, composed of three groups of patients: 1. patients with checkpoint-induced colitis, 2. ulcerative colitis, and 3. controls undergoing elective procedures (e.g., colorectal cancer screening) who were found to be healthy^66^ (Table S1). We used the provided gene expression matrix following the authors’ quality control, including the provided annotations for T cell clonal expansion. We further applied the autoencoder model for cells having TCR data, and obtained their expansion probability (Figure S27A). We then calculated the area under the ROC curve and visualized the distribution of the expansion probability, stacked by the true expansion status provided by the authors (Figure S27B). Lastly, we calculated the fraction of truly expanded T cells out of all the cells having TCR data per sample, and compared it with the fraction of the predicted expanded T cells per sample, using a naive 0.5 threshold (Figure S27C).

### Quantification and statistical analysis

The quantitative and statistical analyses are described thoroughly in the relevant sections of the method details. Explicit *p*-values and sample sizes are embedded in the figures and/or figure legends, and are described throughout the text.

## Supplemental information

Document S1. Figures S1–S27

Table S1. Summary of utilized paired scRNA/TCR-seq datasets, related to Figures 1, 3, 5, 6, S1, S8–S11, S26, and S27

Table S2. Model hyperparameters and performance metrics, related to Figures 2, 4, S2–S4, and S17

Table S3. Model performance across external independent test datasets, related to Figures 3, 5, S8–S10, and S20

Document S2. Article plus supplemental information
