# Supplementary material for: scXpand: Pan-cancer detection of T cell clonal expansion from single-cell RNA sequencing without paired single-cell TCR sequencing
Source: Cell Genom. 2026 Aug 7;6(9):101328. doi: 10.1016/j.xgen.2026.101328 (PMC13576724; doi:10.1016/j.xgen.2026.101328)
Supplement: Supplementary file 1 [file mmc1.pdf]

Cell Genomics, Volume *6*

Supplemental information

scXpand: Pan-cancer detection of T cell clonal expansion from single-cell RNA sequencing without paired single-cell TCR sequencing

Ofir Shorer, Ron Amit, and Keren Yizhak

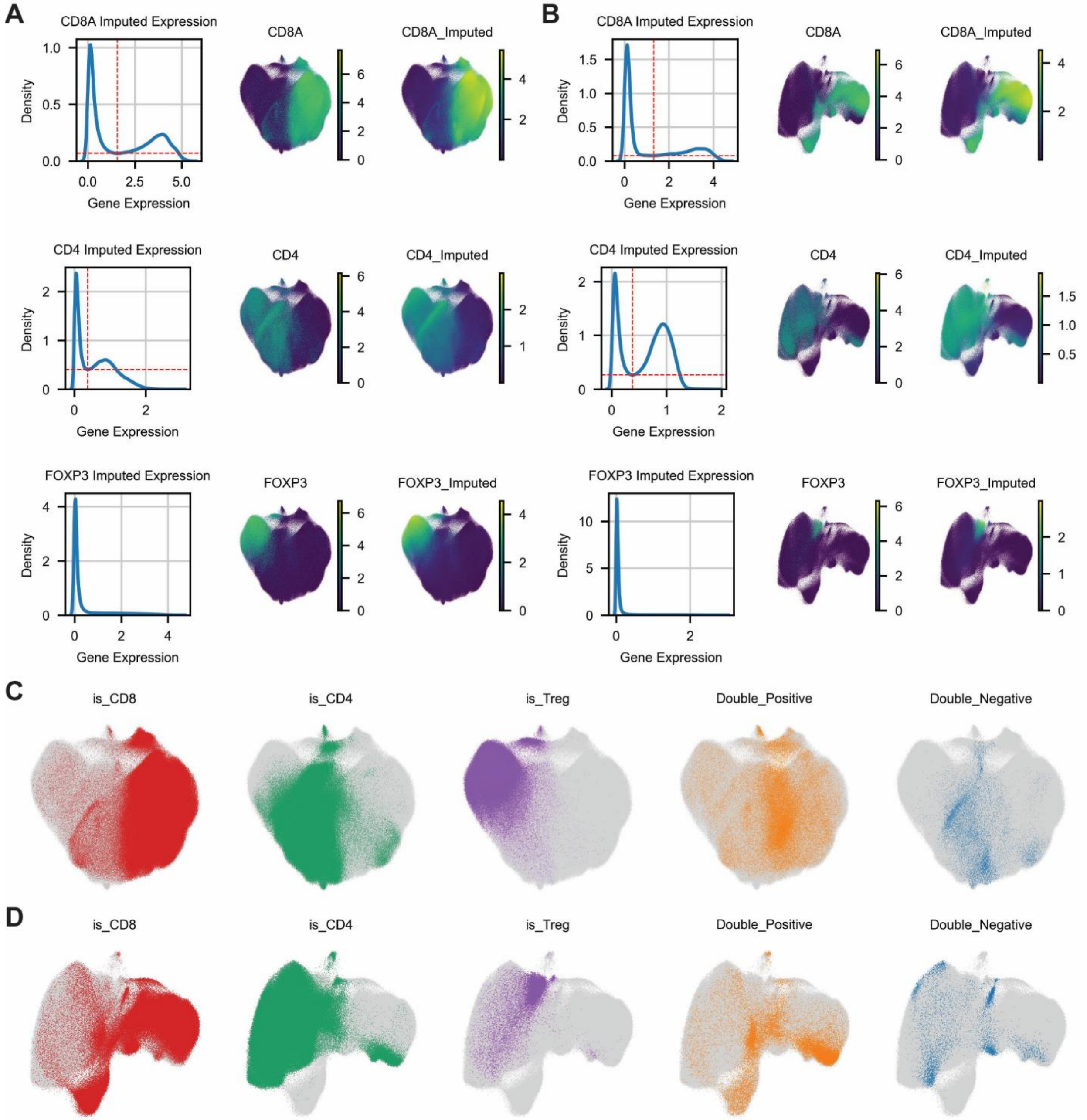

Figure S1. Markov Affinity-based Graph Imputation of Cells (MAGIC)[1] for detection of sequencing drop-outs, Related to Figure 1. A-B. Bimodal density curves for MAGIC-imputed gene expression of *CD8A*, *CD4* and *FOXP3* (left), and their corresponding uniform manifold approximation and projection (UMAP)[2] plots (right), showing imputed and non-imputed gene expression for single cells from tumor samples (A) and blood samples (B). Vertical red dashed lines represent expression thresholds. C-D. Labeling of single cells by five subtypes according to imputed and non-imputed gene expression in tumor samples (C) and blood samples (D).

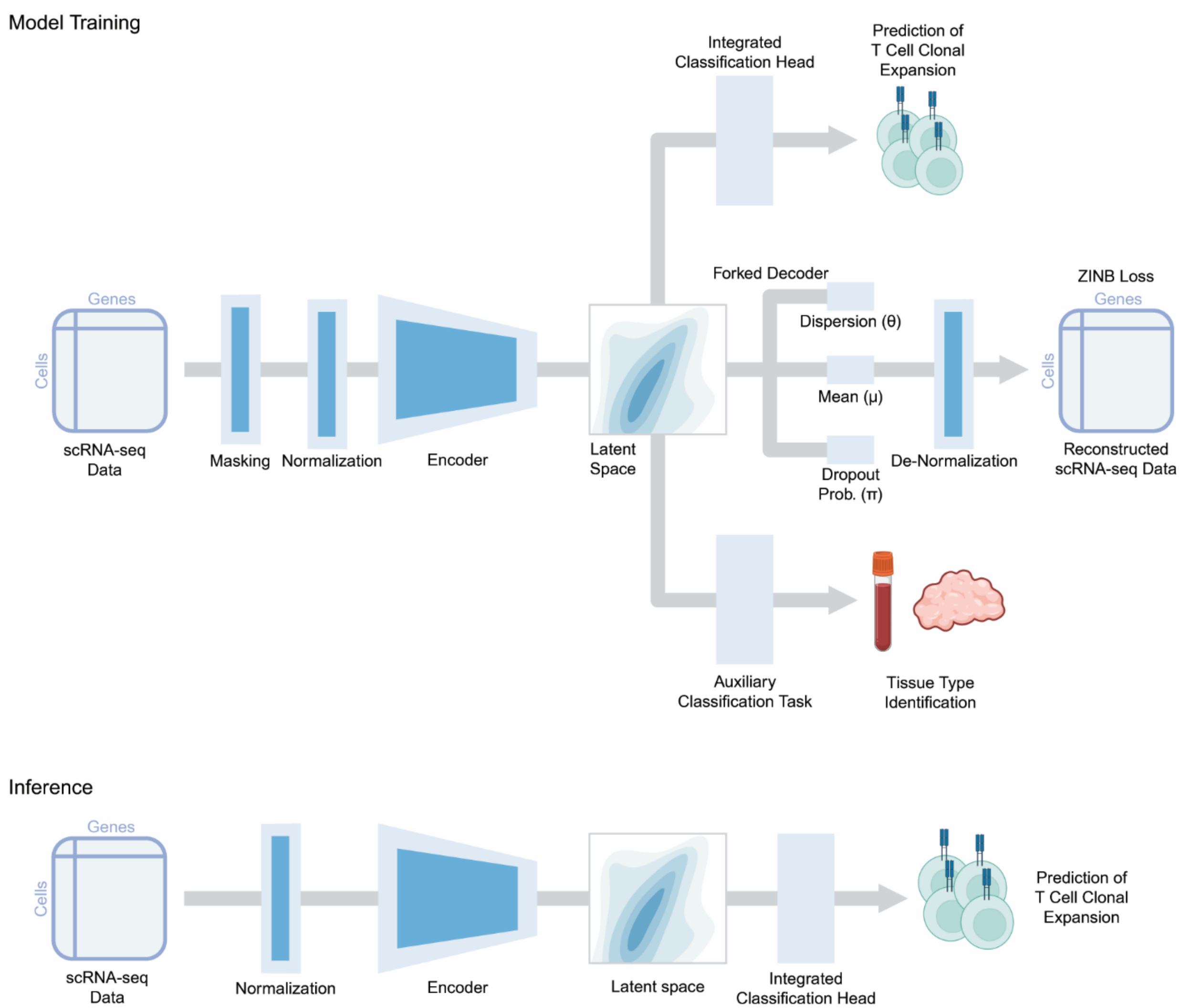

Figure S2. The multi-task autoencoder model architecture, Related to Figure 2. A schematic description of the autoencoder architecture, demonstrating both training (top) and inference (bottom) configurations. scRNA-seq data undergo masking and normalization, with a subsequent forked autoencoder core having a zero-inflated negative binomial reconstruction loss function. Two integrated classification heads are stemming out of the latent vector, with one auxiliary classification task, and the second with the main classification task of predicting T-cell clonal expansion. Inference is being done using the main classification task alone. Importantly, training incorporates clonal expansion status obtained using the paired scTCR-seq data, while inference is being done using scRNA-seq data alone.

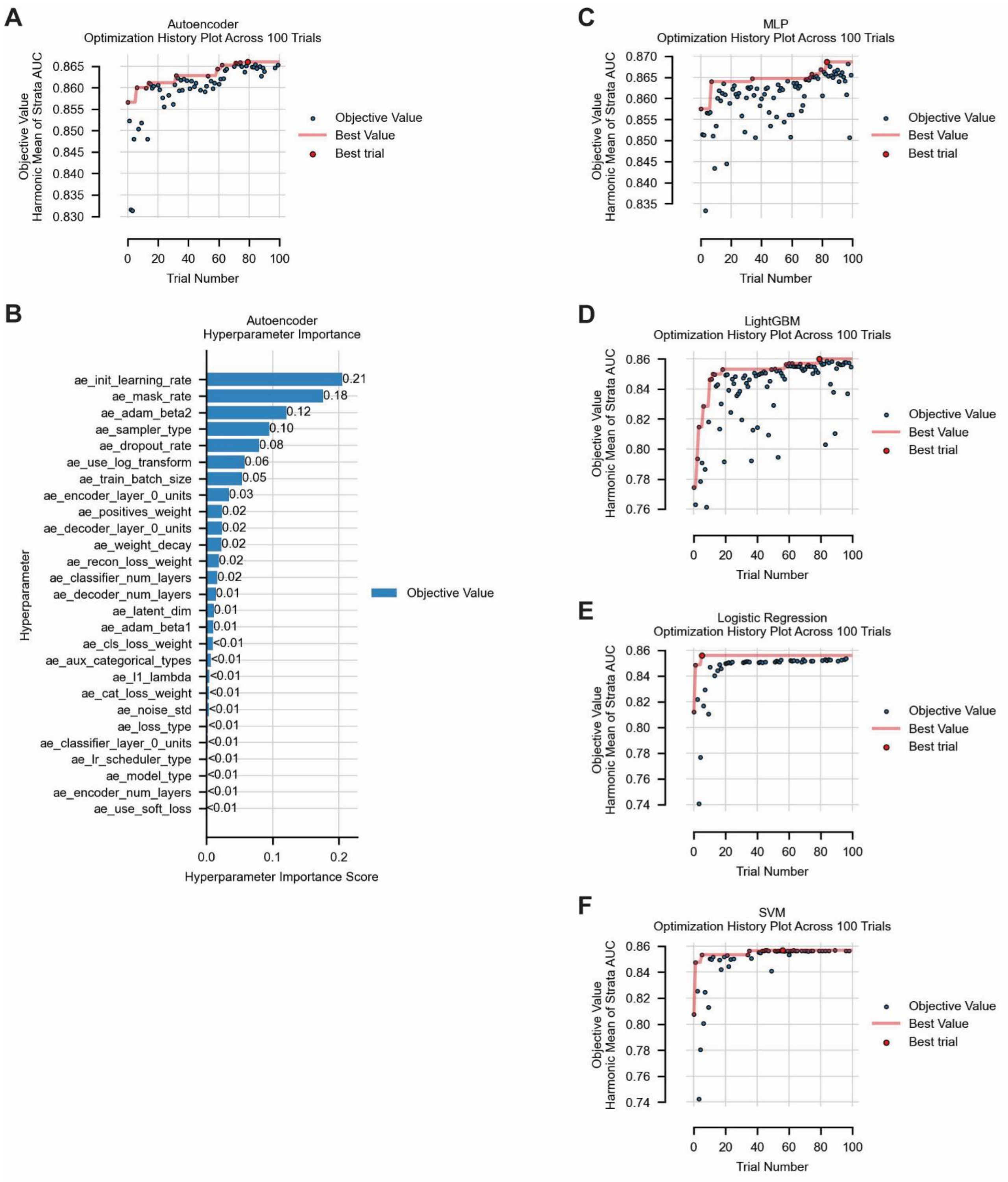

Figure S3. Model optimization procedure using Optuna[3], Related to Figure 2. A-B. Optimization history plot across 100 training trials performed for the autoencoder, depicting changes in model performance across trials (A) and Optuna's hyperparameter importance plot of the autoencoder (B). C-F. Optimization history plots across 100 training trials per model, performed for the MLP, LightGBM, logistic regression, and SVM. Best trial per model is marked with red. Pruned trials are not shown in the history plots. Abbreviations: MLP, multilayer perceptron; SVM, support vector machine.

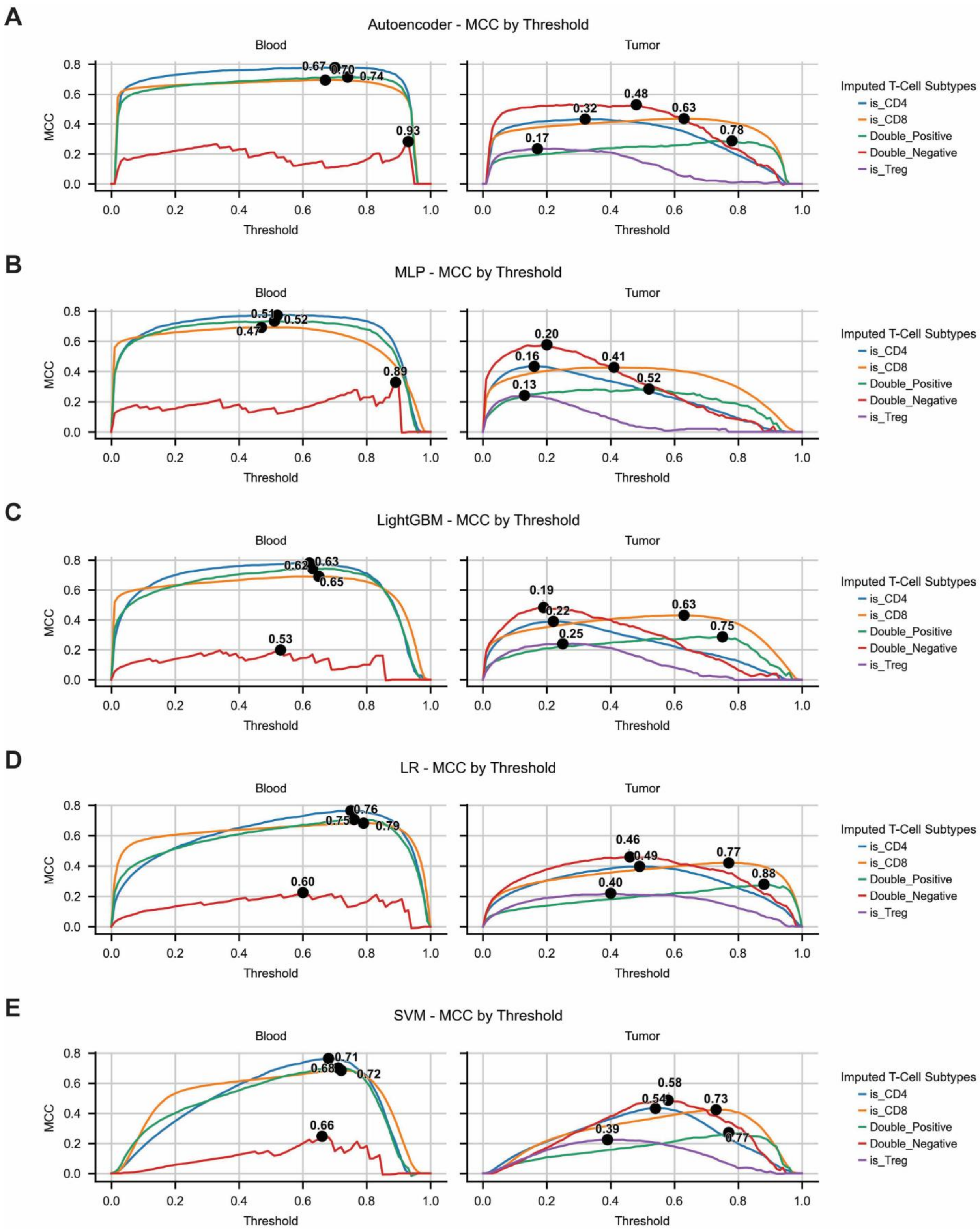

Figure S4. Optimal expansion probability thresholds per model, Related to Figure 2. A-E. Matthews correlation coefficient (MCC)[4,5] distribution by expansion probability threshold per combination of tissue-type and T-cell subtype in each model. Distribution was calculated using the validation set with optimal threshold in each combination marked with black circle. Please note that expanded Tregs were not present

in blood samples of the validation set, with just 8 expanded Tregs in blood samples of the train set. Therefore their MCC distribution in blood samples was removed from this visualization. Abbreviations: MLP, multilayer perceptron; LR, logistic regression; SVM, support vector machine.

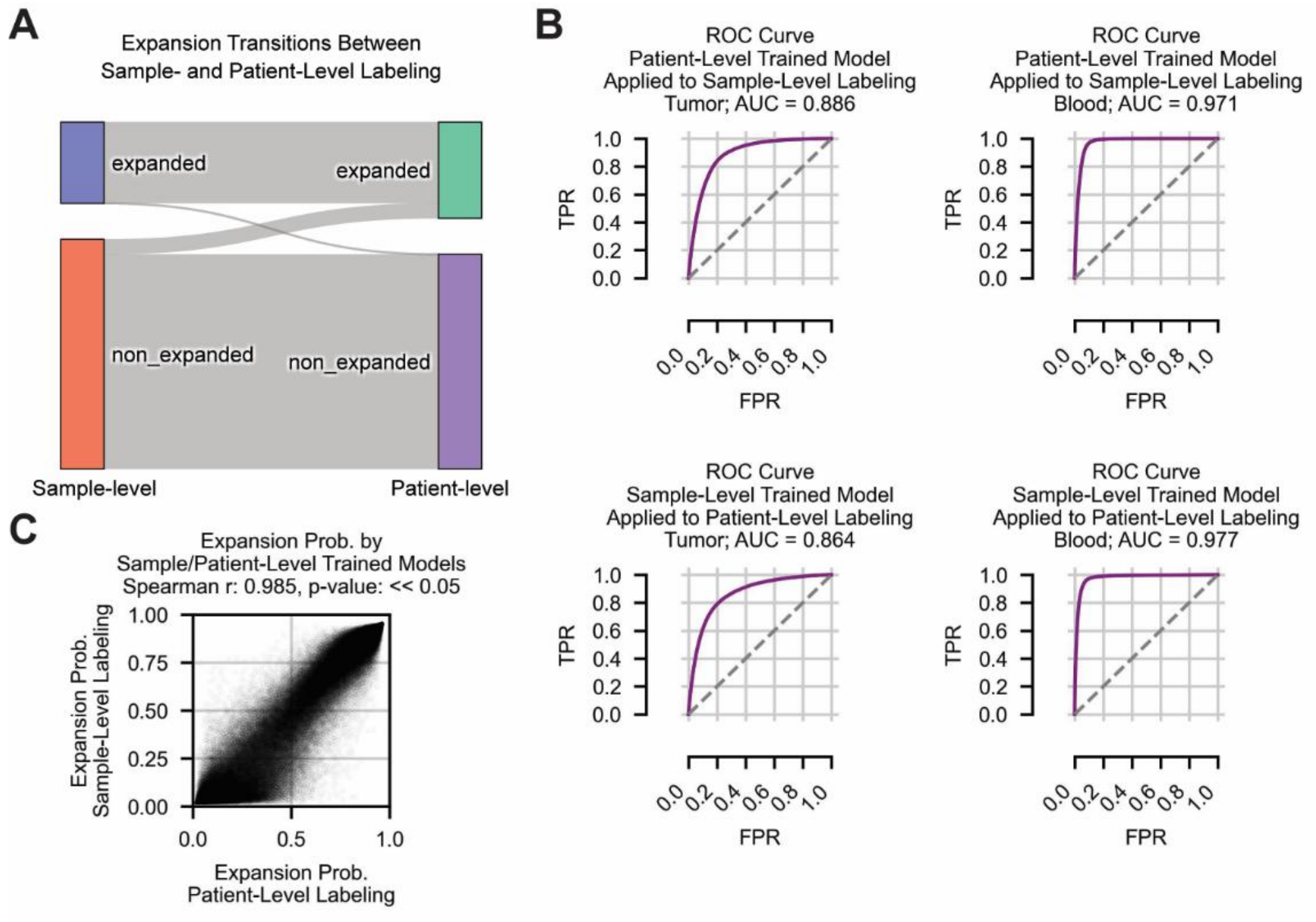

Figure S5. Comparison of sample-level- and patient-level labeling of T-cell clones, Related to Figure 2. A. Sankey plot depicting changes of clonal expansion labeling across T cells from the validation set, when labeled using a sample-wise approach (left), or patient-level approach (right). B. Receiver operating characteristic (ROC) curves, with calculation of the corresponding area under the curves (AUCs), stratified by tumor and blood samples from the validation set, obtained using an autoencoder model trained using patient-level labeling applied to T cells having sample-level labeling (top), as well as an autoencoder model trained using sample-level labeling applied to T cells having patient-level labeling (bottom). C. Scatter plot demonstrating the Spearman correlation between the expansion probabilities obtained using the patient-level- and the sample-level-trained autoencoders, across all T cells from the validation set. P-value was calculated using Spearman’s rank correlation test.

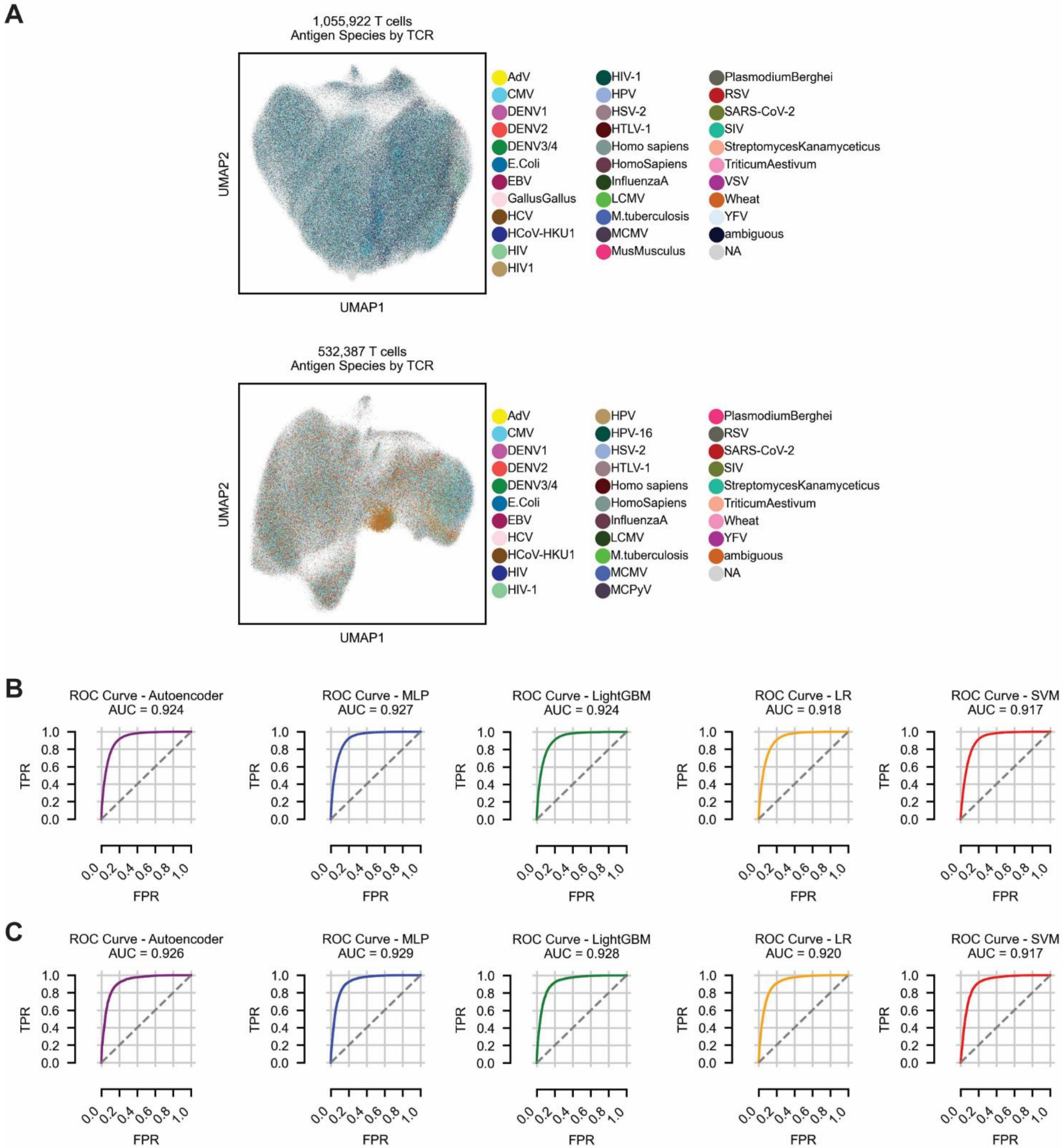

Figure S6. Evaluation of scXpand by epitope identity, Related to Figure 2. A. UMAP plot of antigen species attributed to each single cell following epitope annotations using VDJdb[6], showing tumor (top) and blood samples (bottom). B-C. Receiver operating characteristic (ROC) curves, with calculation of the corresponding area under the curves (AUCs), for each one of the five trained and optimized models, using the validation set. AUCs were calculated separately for T cells that potentially target cancer neoantigens (B) and viral-specific antigens (C). Abbreviations: MLP, multilayer perceptron; LR, logistic regression; SVM, support vector machine.

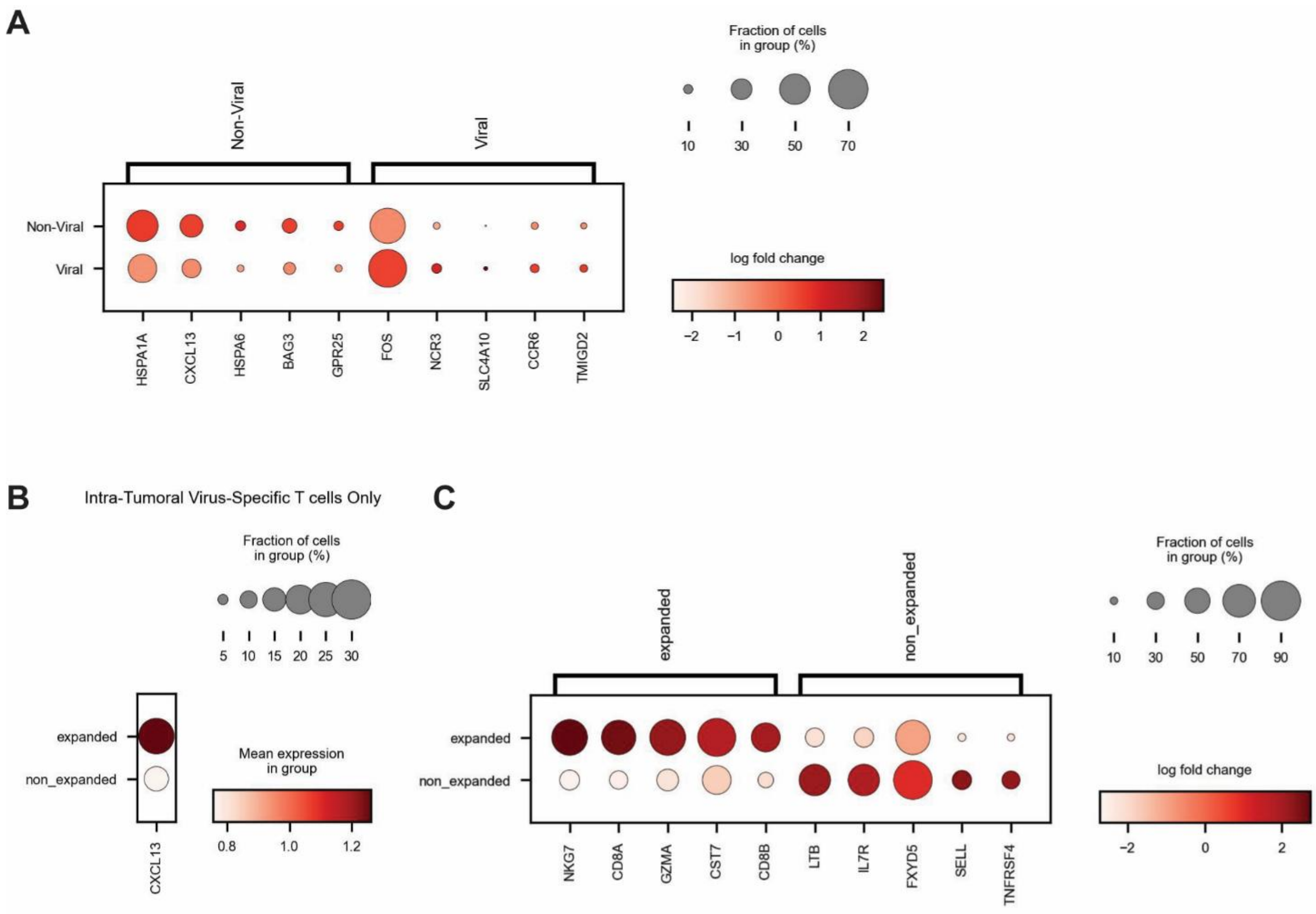

Figure S7. Expansion of virus-specific and non-virus-specific T cells across tumor samples from the entire training dataset, Related to Figure 2. A. Dotplot showing the top 5 differentially expressed genes between expanded virus-specific and non-virus-specific T cells. B. Dotplot showing the expression of *CXCL13* within expanded and non-expanded virus-specific T cells. C. Dotplot showing the top 5 differentially expressed genes between expanded and non-expanded virus-specific T cells.

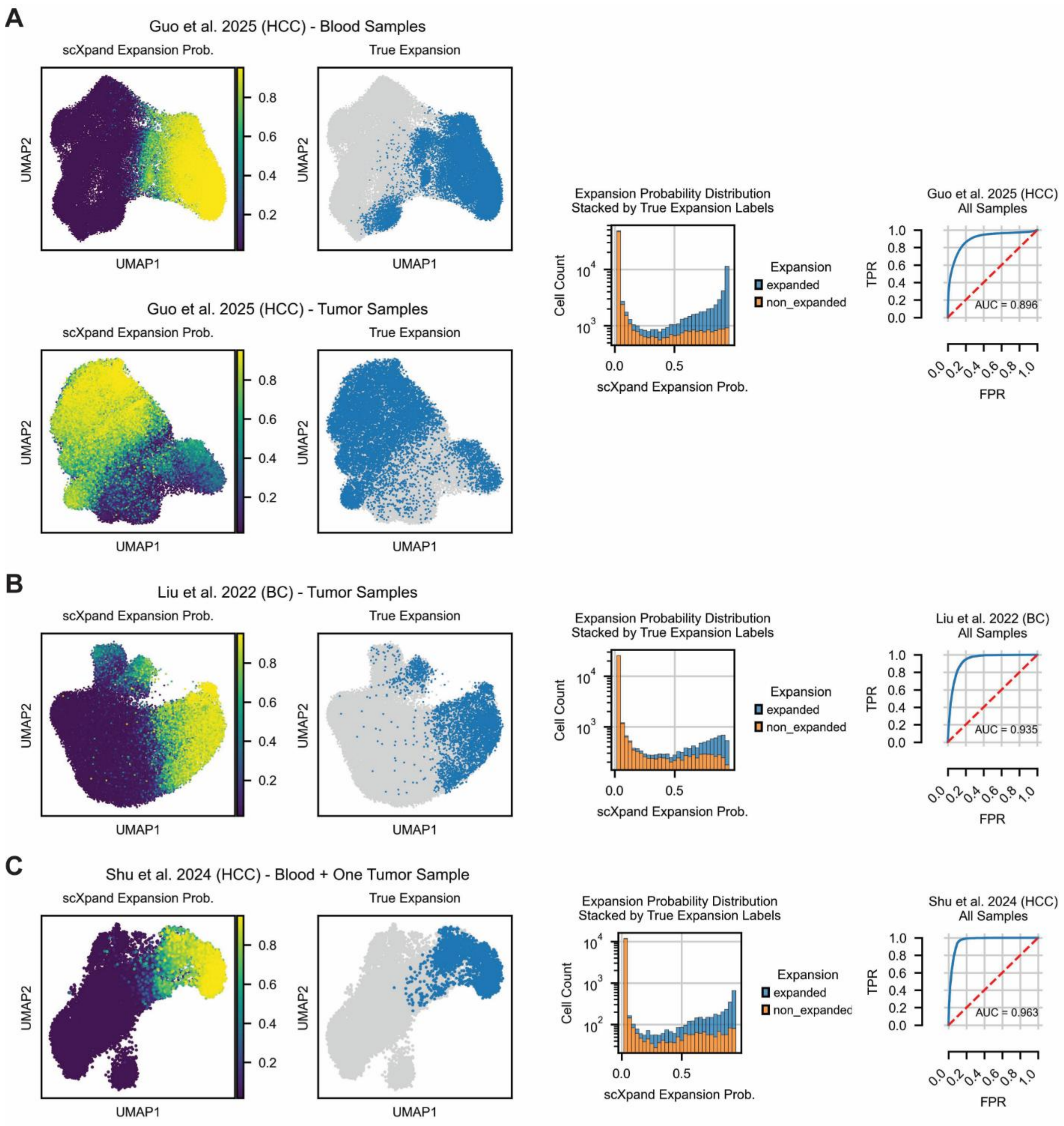

Figure S8. Application of the model to additional external test datasets, Related to Figure 3. A-C. Application of the trained autoencoder model to three different test datasets[7–9], consisting of different cancer types across tumor and blood samples. UMAP plots demonstrating the predicted expansion probability for each T cell, as well as the true expansion labels (left), including a histogram of the probability distribution stacked by the true expansion labels, with the corresponding ROC curves (right). Abbreviations: FPR, false positive rate; TPR, true positive rate.

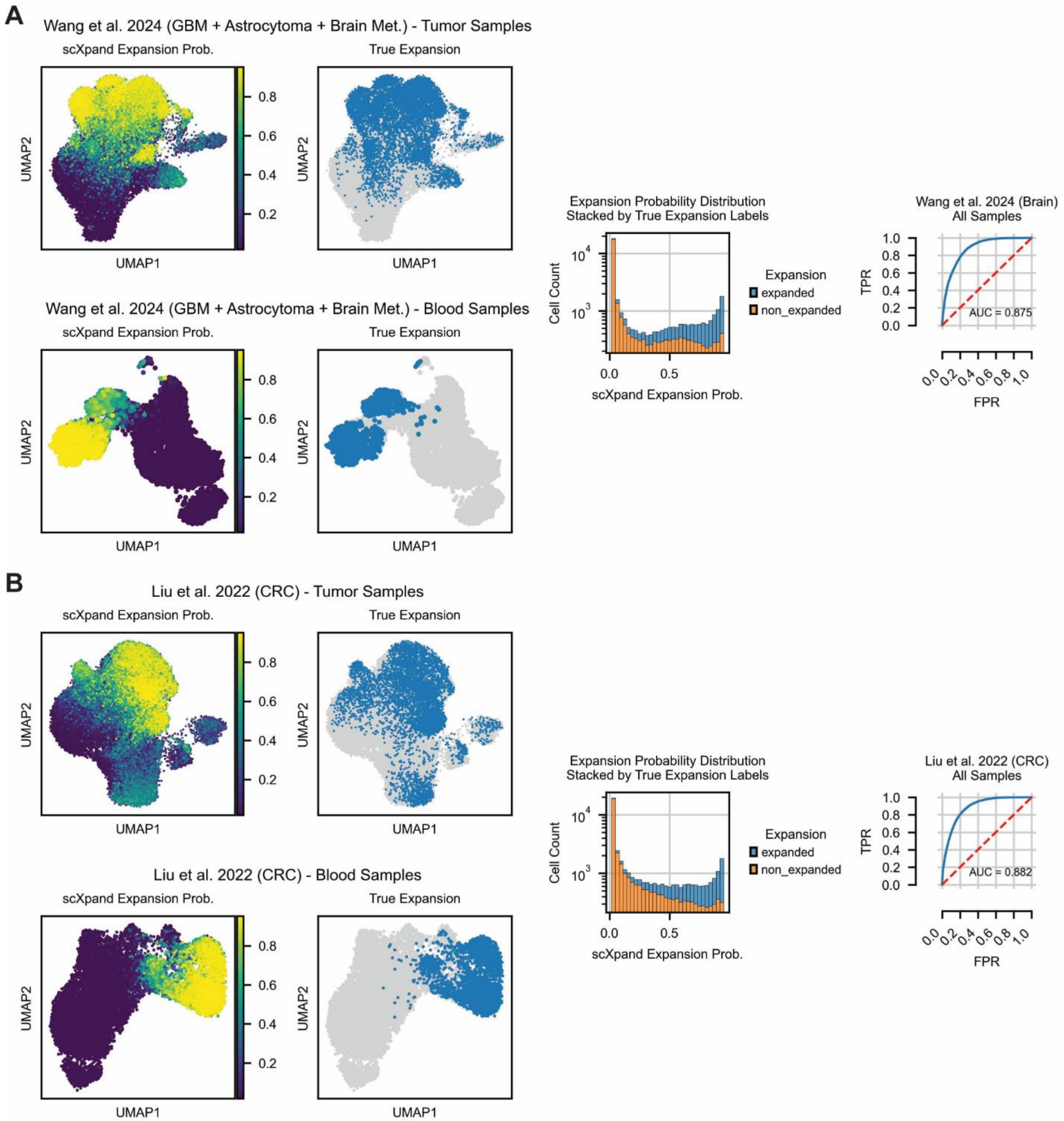

Figure S9. Application of the model to additional external test datasets, Related to Figure 3. A-B. Application of the trained autoencoder model to two different test datasets[10,11], consisting of different cancer types across tumor and blood samples. UMAP plots demonstrating the predicted expansion probability for each T cell, as well as the true expansion labels (left), including a histogram of the probability distribution stacked by the true expansion labels, with the corresponding ROC curves (right). Abbreviations: FPR, false positive rate; TPR, true positive rate.

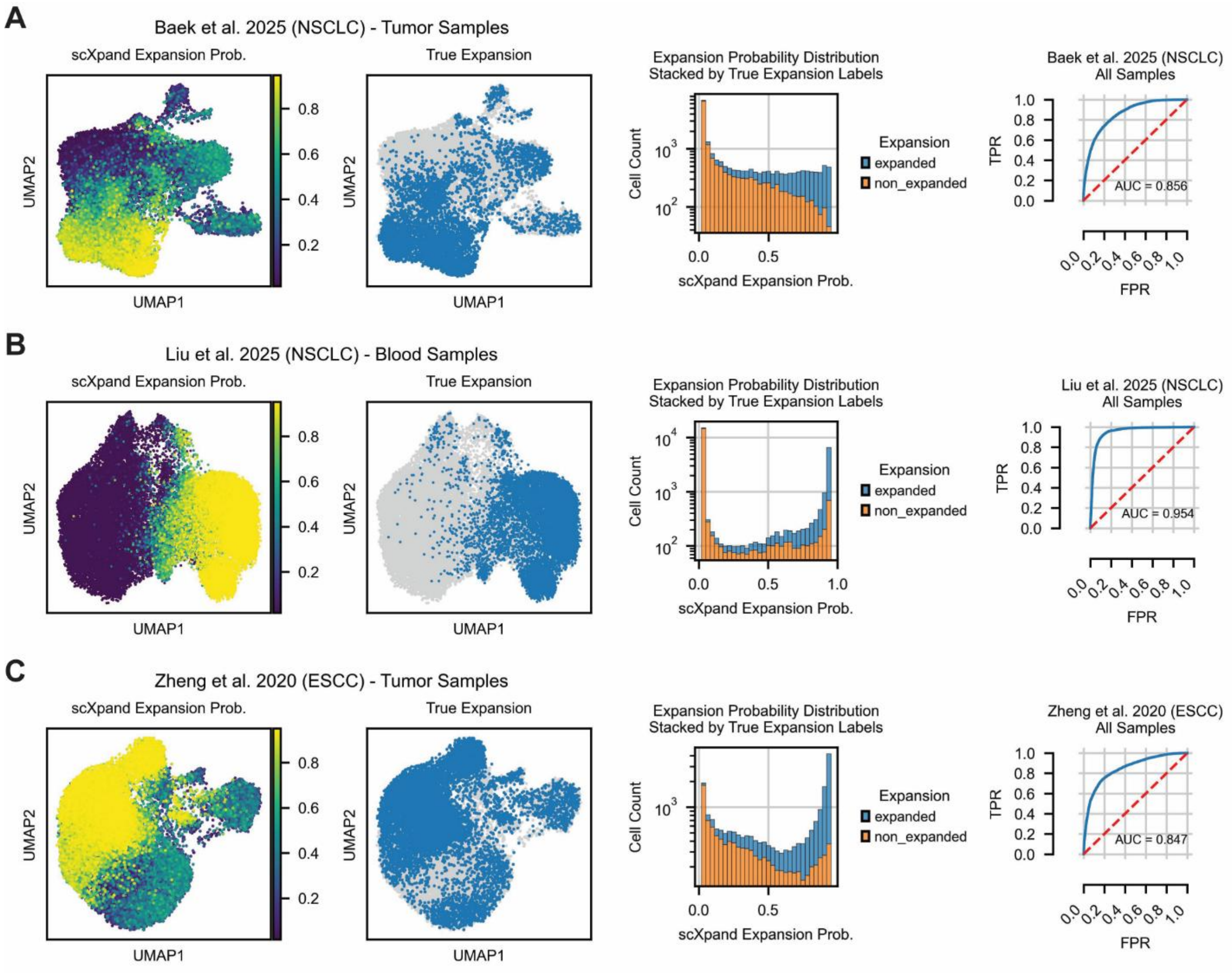

Figure S10. Application of the model to additional external test datasets, Related to Figure 3. A-C. Application of the trained autoencoder model to three different test datasets[12–14], consisting of different cancer types across tumor and blood samples. UMAP plots demonstrating the predicted expansion probability for each T cell, as well as the true expansion labels (left), including a histogram of the probability distribution stacked by the true expansion labels, with the corresponding ROC curves (right). Abbreviations: FPR, false positive rate; TPR, true positive rate.

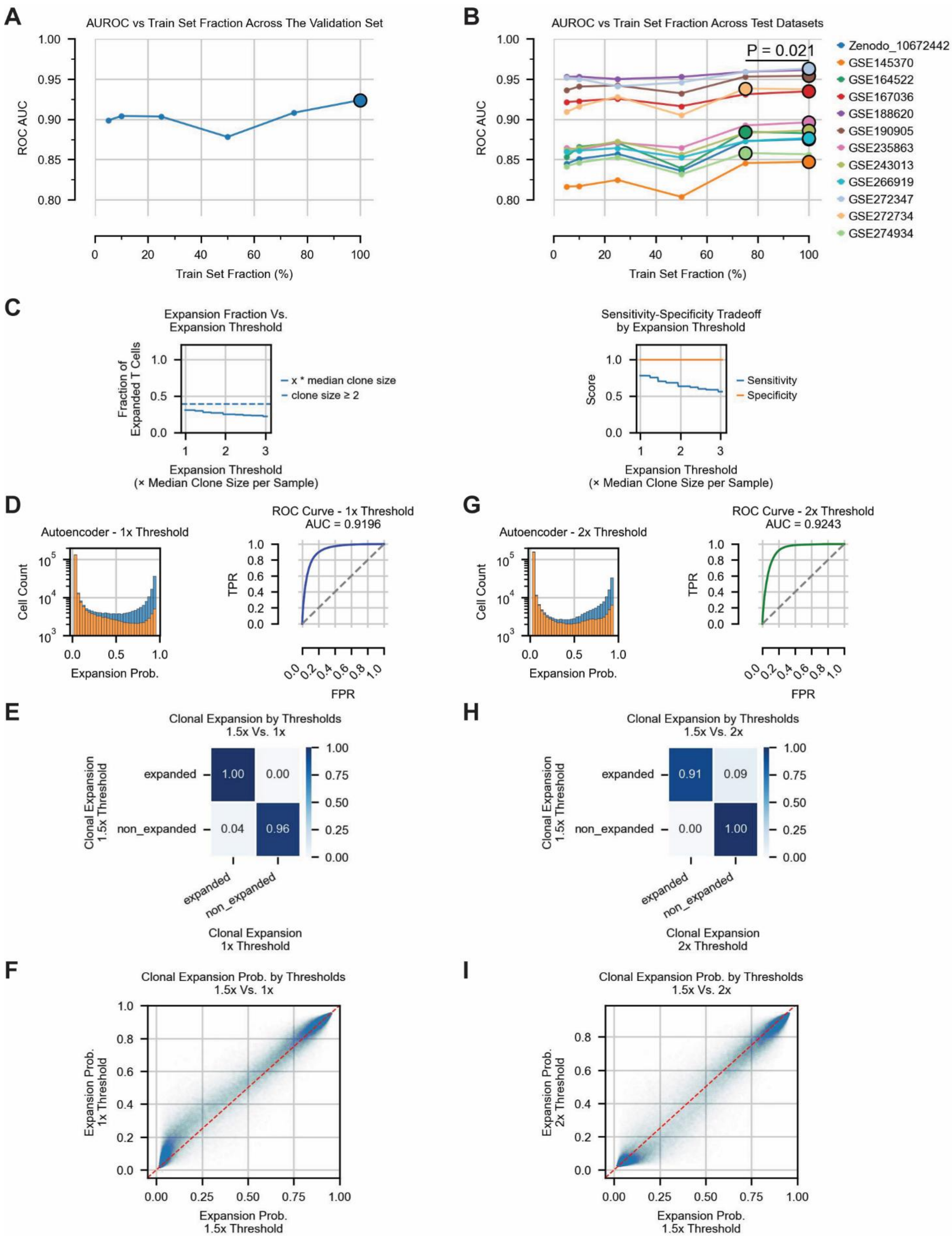
A
AUROC vs Train Set Fraction Across The Validation Set
ROC AUC
Train Set Fraction (%)
B
AUROC vs Train Set Fraction Across Test Datasets
P = 0.021
Zenodo_10672442
GSE145370
GSE164522
GSE167036
GSE188620
GSE190905
GSE235863
GSE243013
GSE266919
GSE272347
GSE272734
GSE274934
C
Expansion Fraction Vs. Expansion Threshold
Fraction of Expanded T Cells
x * median clone size
clone size ≥ 2
Expansion Threshold (× Median Clone Size per Sample)
Sensitivity-Specificity Tradeoff by Expansion Threshold
Score
Sensitivity
Specificity
D
Autoencoder - 1x Threshold
Cell Count
Expansion Prob.
ROC Curve - 1x Threshold AUC = 0.9196
TPR
FPR
G
Autoencoder - 2x Threshold
ROC Curve - 2x Threshold AUC = 0.9243
E
Clonal Expansion by Thresholds 1.5x Vs. 1x
Clonal Expansion 1.5x Threshold
expanded
non_expanded
Clonal Expansion 1x Threshold
H
Clonal Expansion by Thresholds 1.5x Vs. 2x
Clonal Expansion 2x Threshold
F
Clonal Expansion Prob. by Thresholds 1.5x Vs. 1x
Expansion Prob. 1x Threshold
Expansion Prob. 1.5x Threshold
I
Clonal Expansion Prob. by Thresholds 1.5x Vs. 2x
Expansion Prob. 2x Threshold

Figure S11. Training subsampling test and expansion threshold sensitivity test, Related to Figure 3. A-B. Training subsampling test, showing model performance by different fractions of training-set size. Model performance was evaluated separately for the validation set of the sub-sampled data (A), as well as for all the twelve test datasets independently (B). A two-sided Wilcoxon signed-rank test was used to compare the difference between the AUROCs obtained by using the entire training-set size and a 0.75 fraction of the training set (p-value = 0.021). C. Expansion threshold sensitivity test showing the fraction of T cells labeled as 'expanded' per expansion threshold across the entire training data, including the fraction of non-singletons indicated by dashed line (left), as well as changes of sensitivity score across the different expansion thresholds (right). D. Histogram depicting the number of T cells from the validation set, across the predicted expansion probabilities obtained for each T cell using a model trained with an expansion threshold of 1x the median number of cells found in each clonotype per sample, while excluding singletons. Bars are colored and stacked based on the true expansion labels of each T cell (left), with the corresponding ROC curve (right). E. Confusion matrix showing the fraction of T cells labeled as 'expanded' or 'non-expanded', based on different expansion thresholds across the validation set. F. Scatter plot demonstrating differences in expansion probability for each T cell, as predicted for the validation set, by models trained using different expansion thresholds. G-I. Similar plots to those in D-F, corresponding to expansion threshold of 2x the median number of cells found in each clonotype per sample, while excluding singletons. Results are presented using an autoencoder-based model.

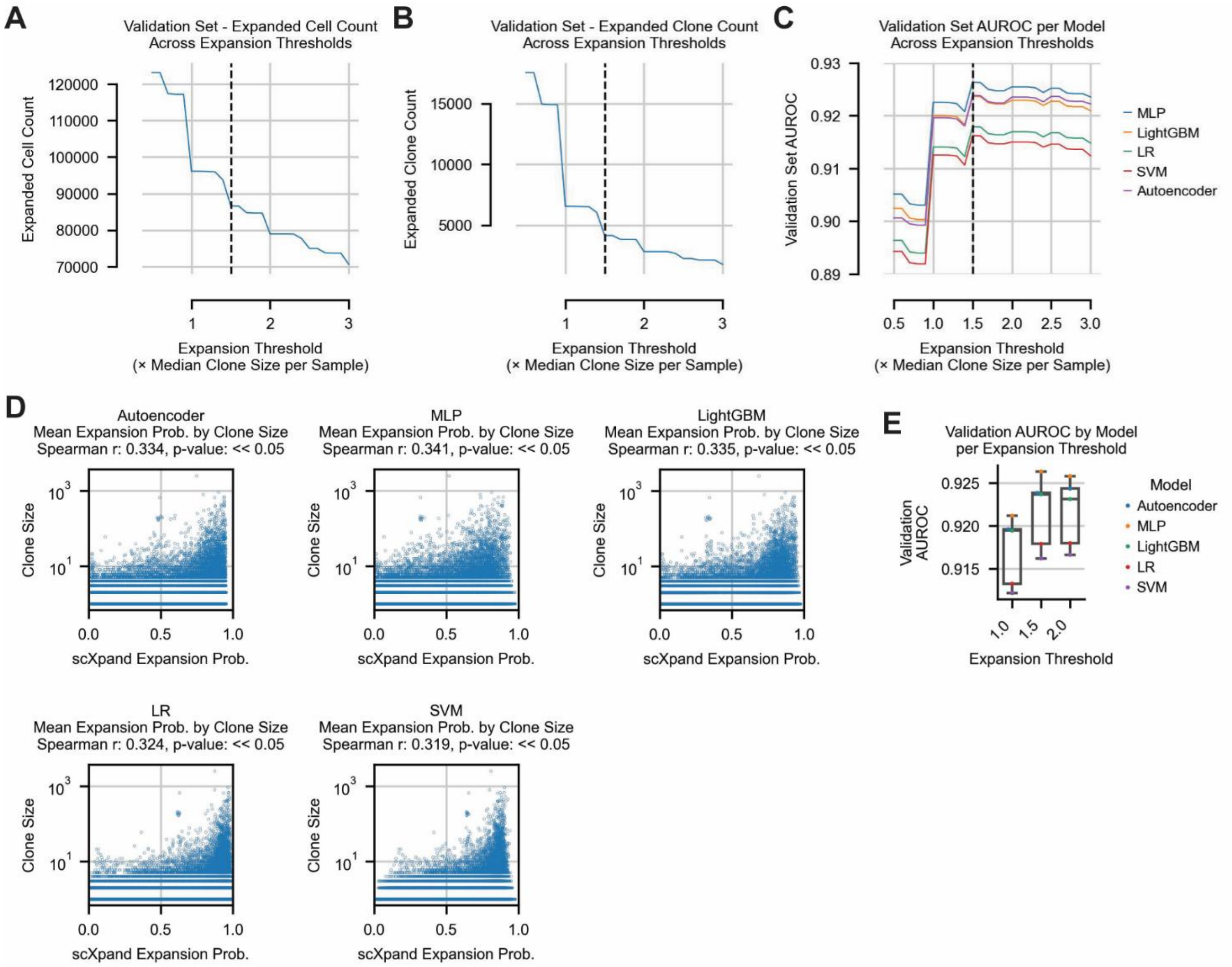

Figure S12. Comparative threshold analysis using the validation set, Related to Figure 3. A-B. Quantification of T cells (A) and T-cell clones (B) labeled as ‘expanded’ across different expansion thresholds. C. Comparison of validation AUROCs obtained when applying models trained using an expansion threshold of 1.5x the median number of cells found in each clonotype per sample, while excluding singletons, across expansion labels obtained using varying expansion thresholds. D. Scatter plots demonstrating the correlation of the mean predicted expansion probability per clone, with clone size, across different models. P-values were calculated using Spearman’s rank correlation test. E. Validation AUROCs evaluated across the different models, following re-training with additional expansion thresholds of 1x and 2x the median number of cells found in each clonotype per sample, while excluding singletons.

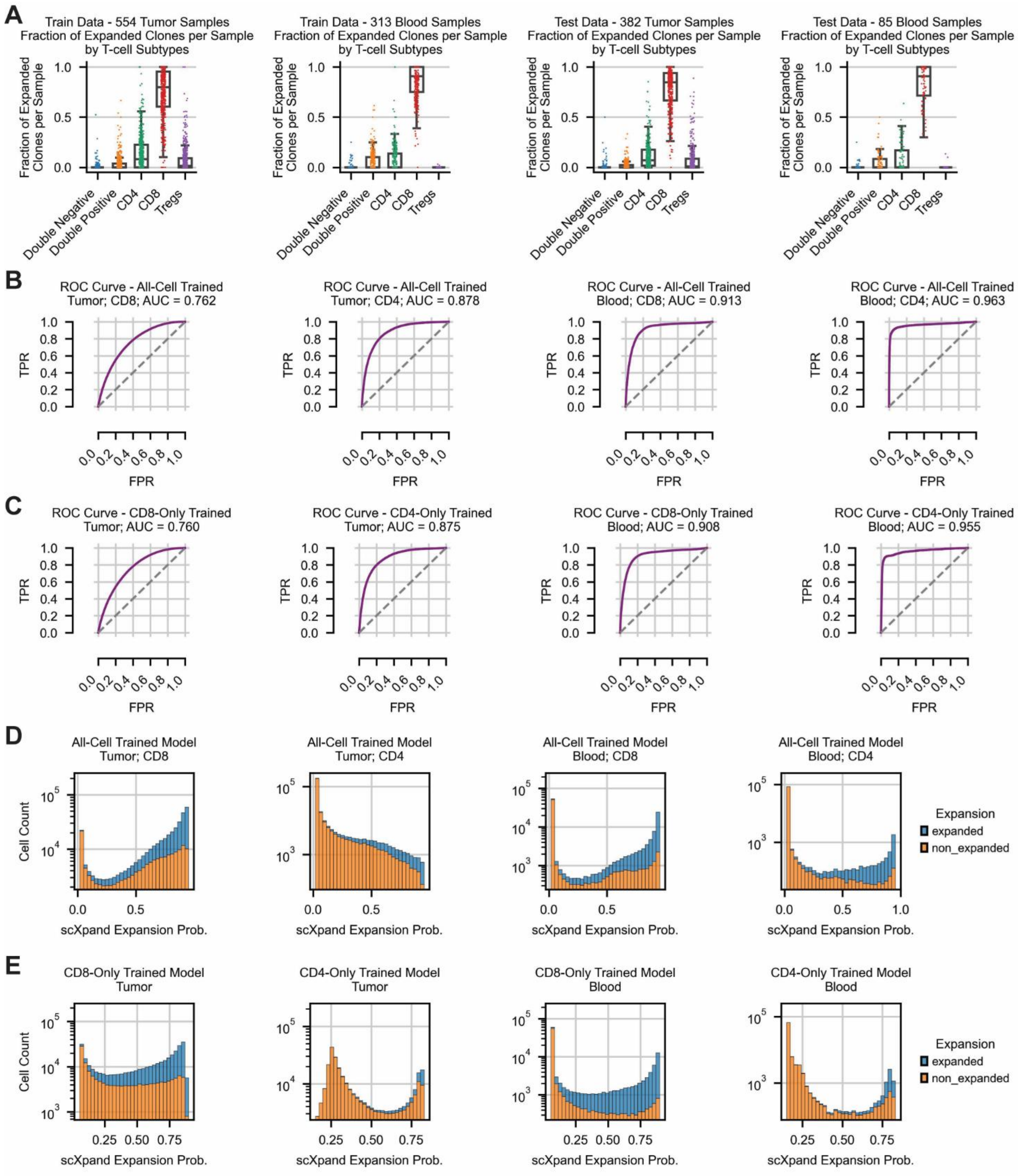

Figure S13. T-cell subtype-specific evaluation using the entire test dataset, Related to Figure 3. A. Boxplots demonstrating the fraction of expanded clones per sample by different T-cell subtypes, out of all the expanded clones per sample, separated for tumor and blood samples across the training dataset (left), and the test dataset (right). Please note that only samples having expanded T-cell clones are presented. B. Receiver operating characteristic (ROC) curves, with calculation of the corresponding area under the curves (AUCs), stratified by $CD8^+$ and $CD4^+$ T cells across tumor (left) and blood samples (right) from the test dataset, obtained using a non-specific autoencoder model trained across all T-cell subtypes. C.

Receiver operating characteristic (ROC) curves, with calculation of the corresponding area under the curves (AUCs), stratified by $CD8^+$ and $CD4^+$ T cells across tumor (left) and blood samples (right) from the test dataset, obtained using two specific autoencoder models trained across either $CD8^+$ or $CD4^+$ T cells alone. D-E. Histograms depicting the number of T cells from the test dataset, across the predicted expansion probabilities obtained for each T cell by each model, including non-specific (D) and subtype-specific models (E). Bars are colored and stacked based on the true expansion labels of each T cell.

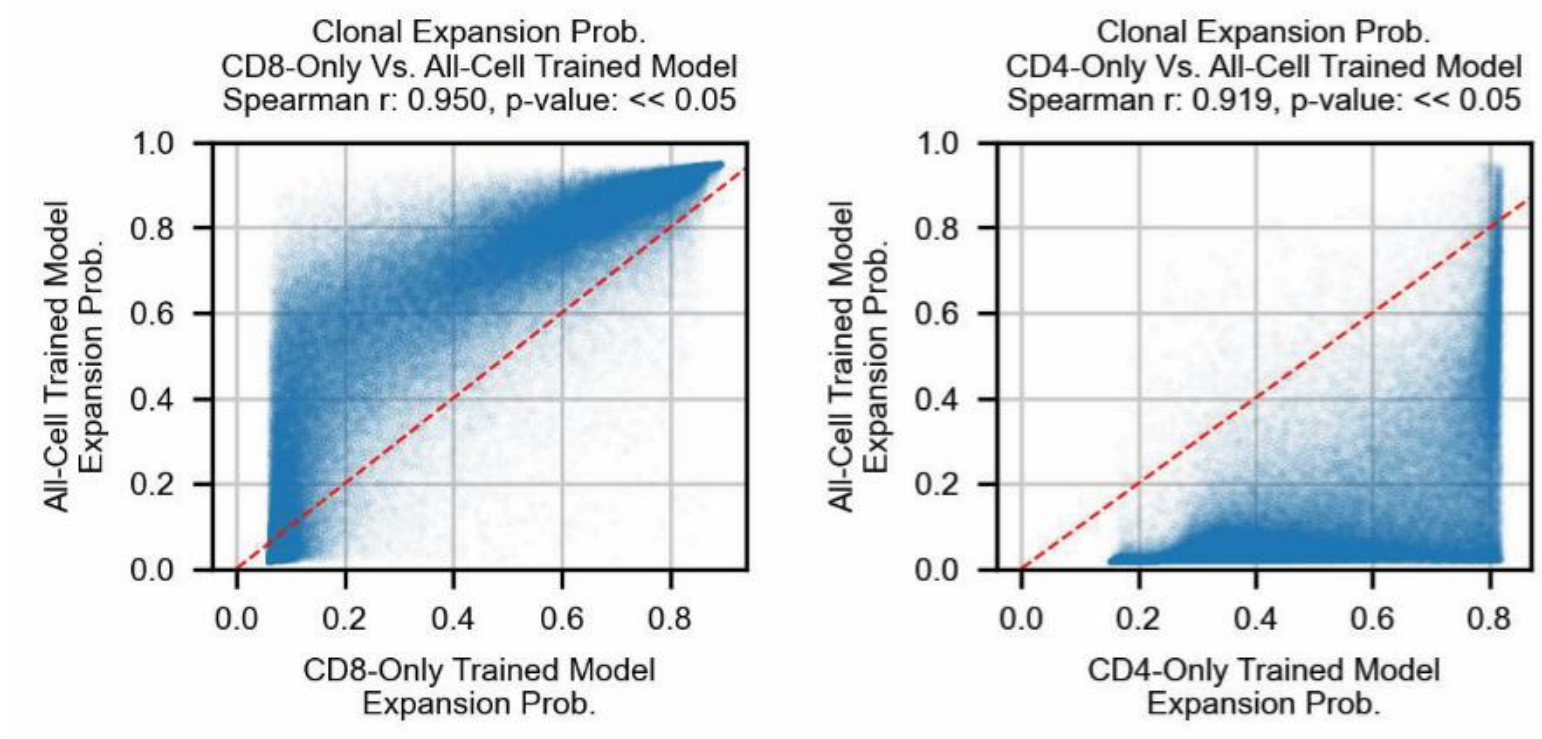

Figure S14. Correlation of the prediction probabilities between the non-specific and the subtype-specific models, Related to Figure 3. Scatter plots demonstrating the Spearman correlation between the mean expansion probabilities predicted for each T-cell clone, obtained using either non-specific or subtype-specific models. Results are shown separately for $CD8^+$ T-cell clones (left) and $CD4^+$ T-cell clones (right) across the entire test dataset. P-values were calculated using Spearman's rank correlation test.

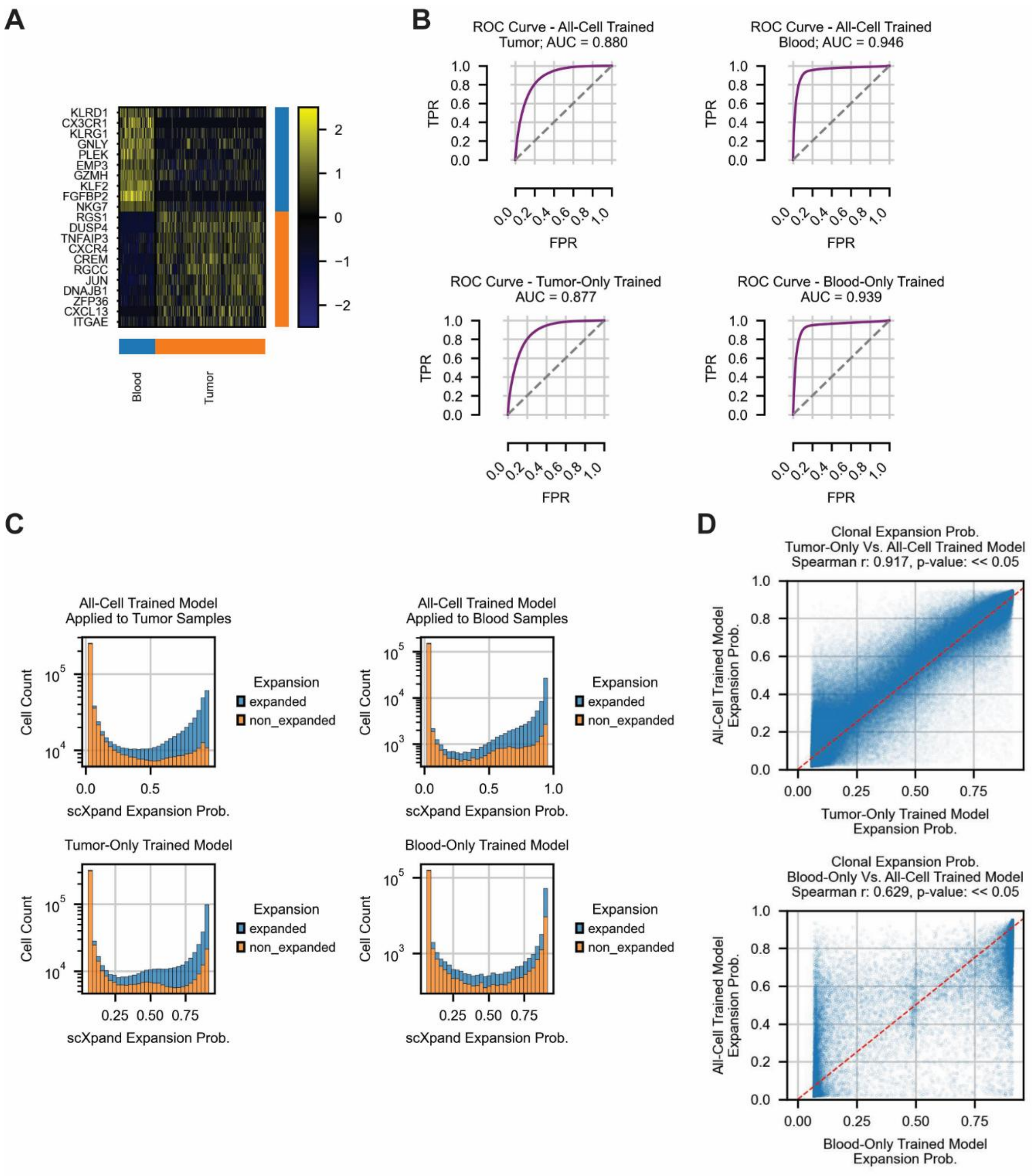

Figure S15. Tissue-type-specific model evaluation, Related to Figure 3. A. Heatmap showing selected list of differentially expressed genes between expanded T cells in tumor and blood samples across the entire training dataset. B. Receiver operating characteristic (ROC) curves, with calculation of the corresponding area under the curves (AUCs), stratified by tumor (left) and blood samples (right) from the test dataset, obtained using a non-specific autoencoder model trained across all tissue types (top), and two specific autoencoder models trained using either tumor or blood samples alone (bottom). C. Histograms depicting the number of T cells from the test dataset, across the predicted expansion probabilities obtained for each T cell by each model, including non-specific (top) and specific models (bottom). Bars are colored and stacked based on the true expansion labels of each T cell. D. Scatter plots demonstrating the Spearman correlation between the mean expansion probabilities predicted for each T-cell clone, obtained using either non-specific or tissue-type-specific models. Results are shown separately for tumor samples (top) and

blood samples (bottom) across the entire test dataset. P-values were calculated using Spearman's rank correlation test.

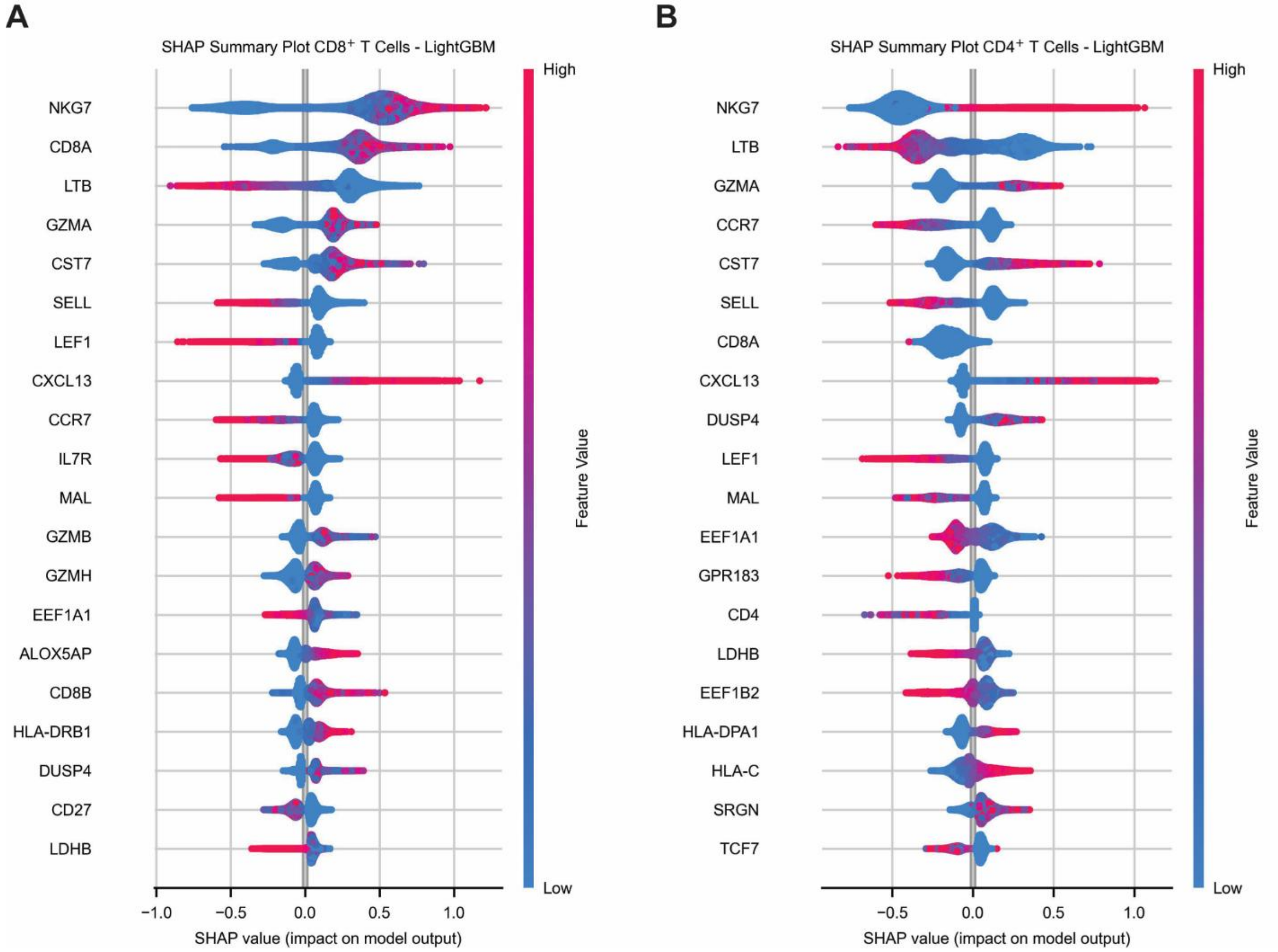

Figure S16. Model explainability and biological interpretation for CD8⁺ and CD4⁺ T cells in tumor samples, Related to Figure 4. A-B. SHAP summary plots depicting the top 20 important genes driving the LightGBM model predictions for CD8⁺ T cells in tumor samples (A) and CD4⁺ T cells in tumor samples (B), using the validation set.

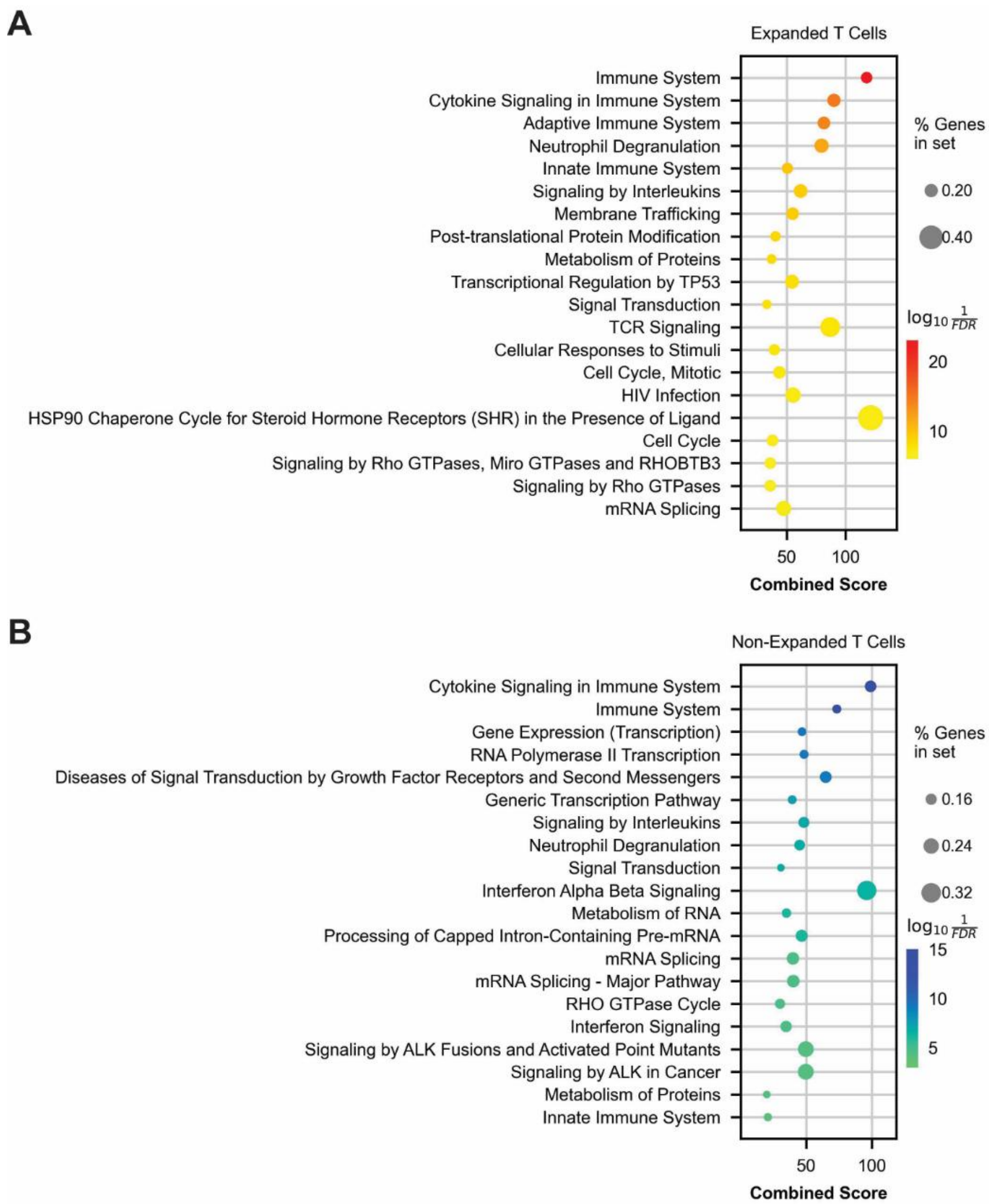

Figure S17. Pathway enrichment analysis for expanded and non-expanded T cells, Related to Figure 4. A-B. Pathway enrichment analysis applied to the top explainable genes obtained using the SHAP analysis for expanded (A) and non-expanded T cells (B) from the validation set, showing the top 20 enriched pathways per group.

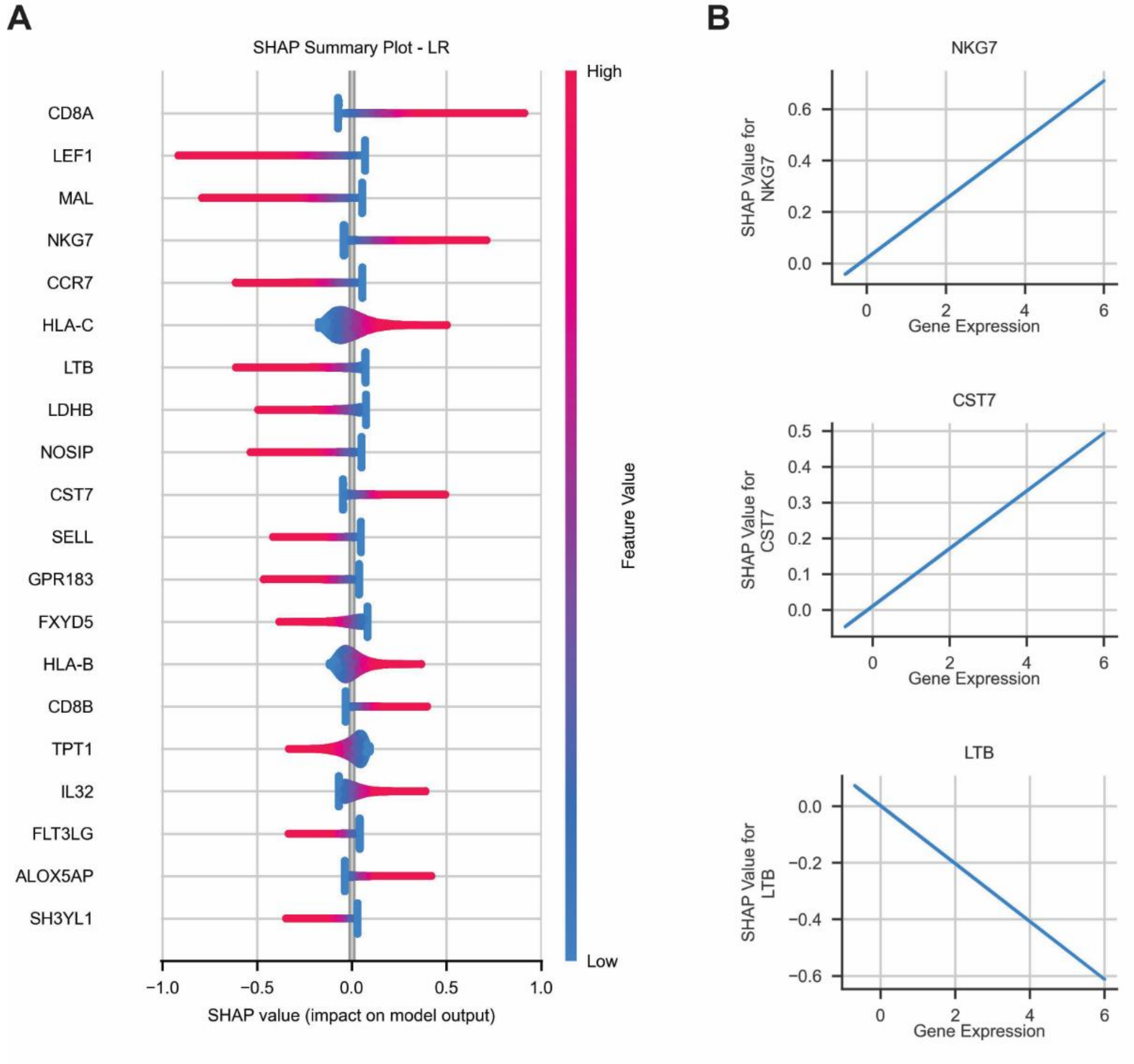

Figure S18. Model explainability and biological interpretation using the logistic regression model, Related to Figure 4. A. SHAP summary plot depicting the top 20 important genes driving the logistic regression model predictions, using all the cells from the validation set. B. SHAP dependency plots showing the effect of gene expression on SHAP values for selected genes. Expression values represent z-scored normalized unique molecular identifier (UMI) counts.

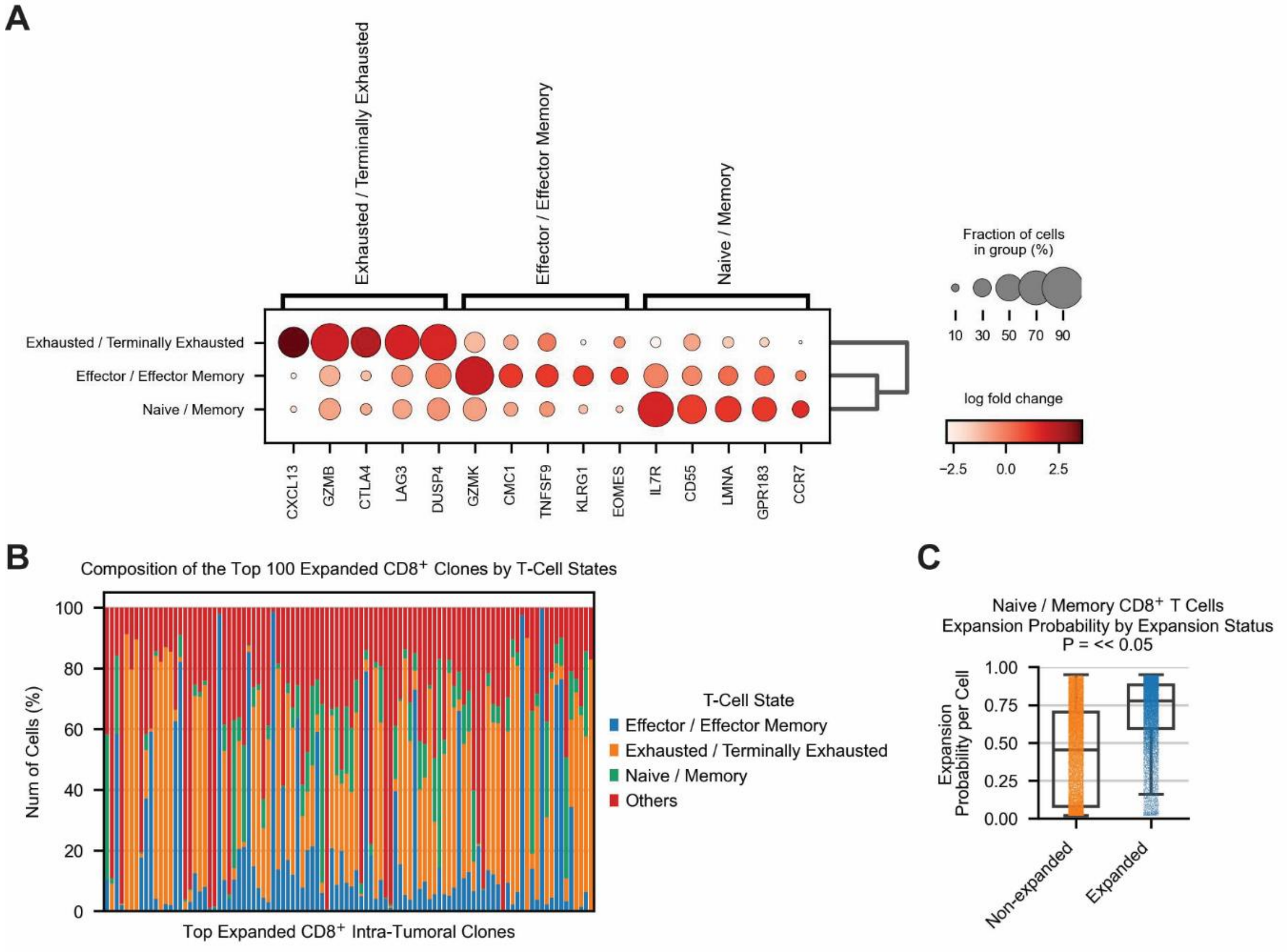

Figure S19. T-cell differentiation states across clones, Related to Figure 5. A. Dotplot showing the top 5 differentially expressed genes per T-cell differentiation state. B. Stacked barplot showing T-cell differentiation state composition across the top 100 expanded clones. C. Box plots demonstrating the difference of the predicted expansion probability, obtained using the autoencoder model, between naive/memory T cells labeled as 'expanded' and 'non-expanded'. P-value was calculated using a two-sided Wilcoxon rank-sum test.

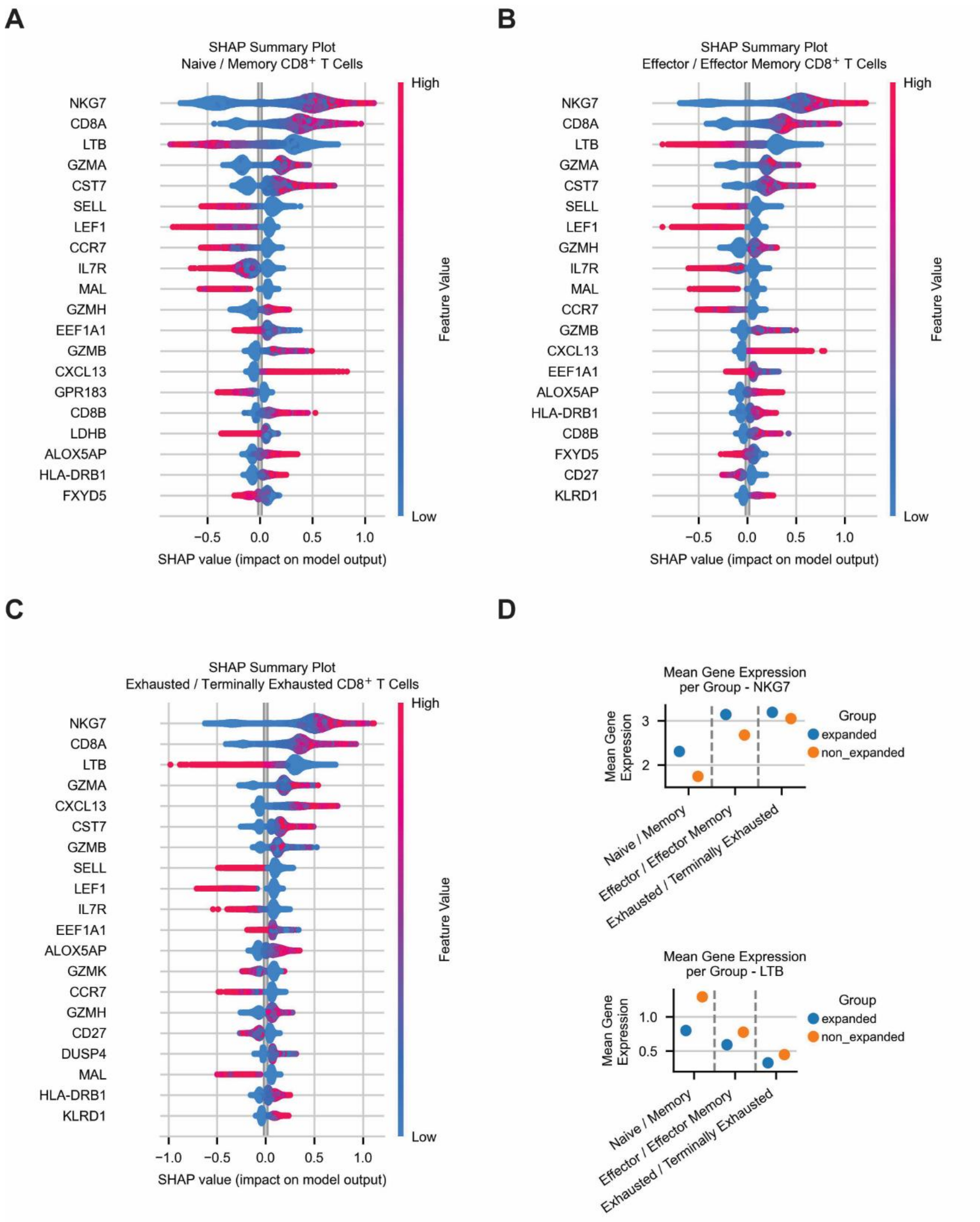

Figure S20. Model explainability across T-cell differentiation states, Related to Figure 5. A-C. SHAP summary plots depicting the top 20 important genes driving the LightGBM model predictions for different T-cell differentiation states, including naive / memory (A), effector / effector-memory (B), and exhausted / terminally exhausted CD8⁺ T cells (C). D. Mean gene expression per T-cell differentiation state, separated by true labels of expanded and non-expanded T-cells, including expression of *NKG7* (top), and *LTB* (bottom).

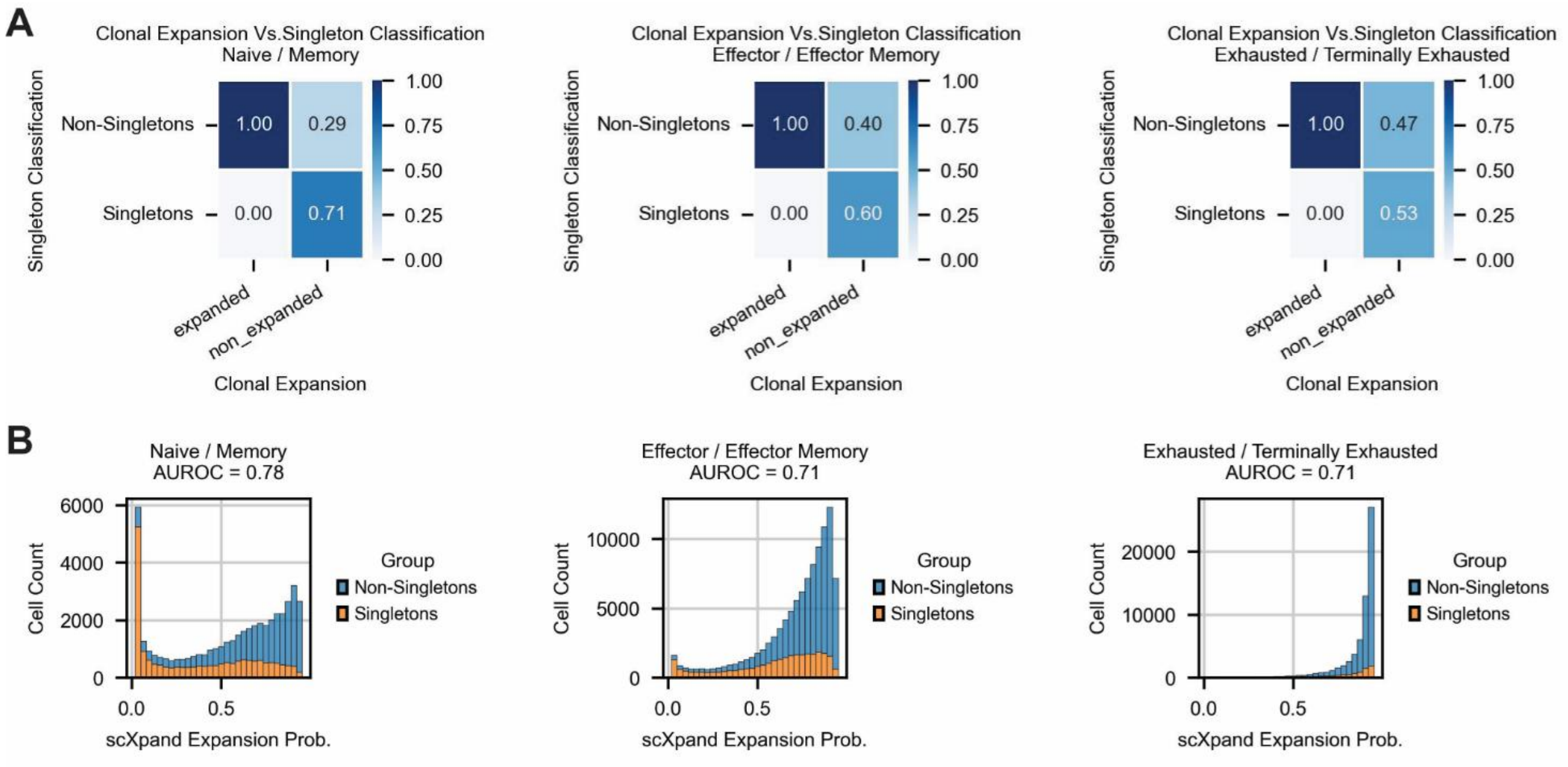

Figure S21. Clonal composition by singletons and non-singletons, across multiple T-cell differentiation states, Related to Figure 5. A. Confusion matrices showing the fraction of singletons and non-singletons by the true clonal expansion status across different T-cell differentiation states. B. Histograms depicting the number of T cells in each differentiation state, across the predicted expansion probabilities obtained for each T cell by the autoencoder. Bars are colored and stacked by T cells labeled as either singletons, or T cells originated in clones with size > 1 (non-singletons).

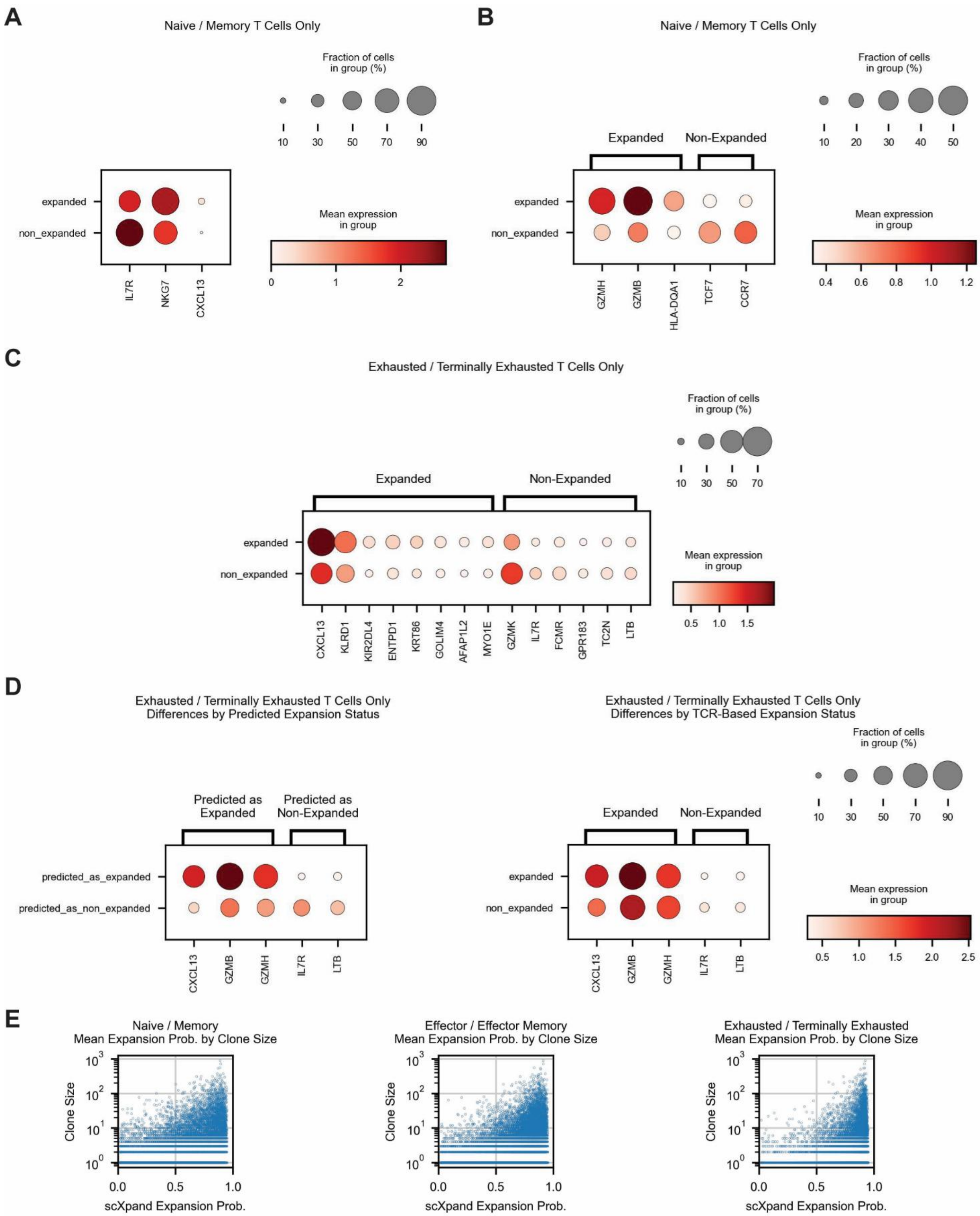

Figure S22. Transcriptional signatures of expanded and non-expanded T cells across multiple T-cell differentiation states, and association of expansion probability with clone size, Related to Figure 5. A. Dotplot demonstrating expression of selected gene markers for both expanded and non-expanded T

cells, within the naive / memory T-cell state, including *IL7R*, *NKG7* and *CXCL13*. B. Dotplot showing the top differentially expressed genes between expanded and non-expanded T cells within the naive / memory T-cell state, depicting transcriptional distinction between both groups within the same cellular state. C. Dotplot showing the top differentially expressed genes between expanded and non-expanded T cells within the exhausted / terminally exhausted T-cell state, depicting transcriptional similarity between both groups within this cellular state, with log-fold change < 1 for all of the top markers. D. Dotplots showing selected differentially expressed genes between T cells predicted as expanded or non-expanded by the autoencoder model, within the exhausted / terminally exhausted T-cell state (left), as well as between T cells labeled according to TCR-based expansion status (right). E. Scatter plots demonstrating the association of the mean predicted expansion probability across T cells from the same cellular state per clone, with clone size. Expansion probabilities were obtained using the autoencoder model.

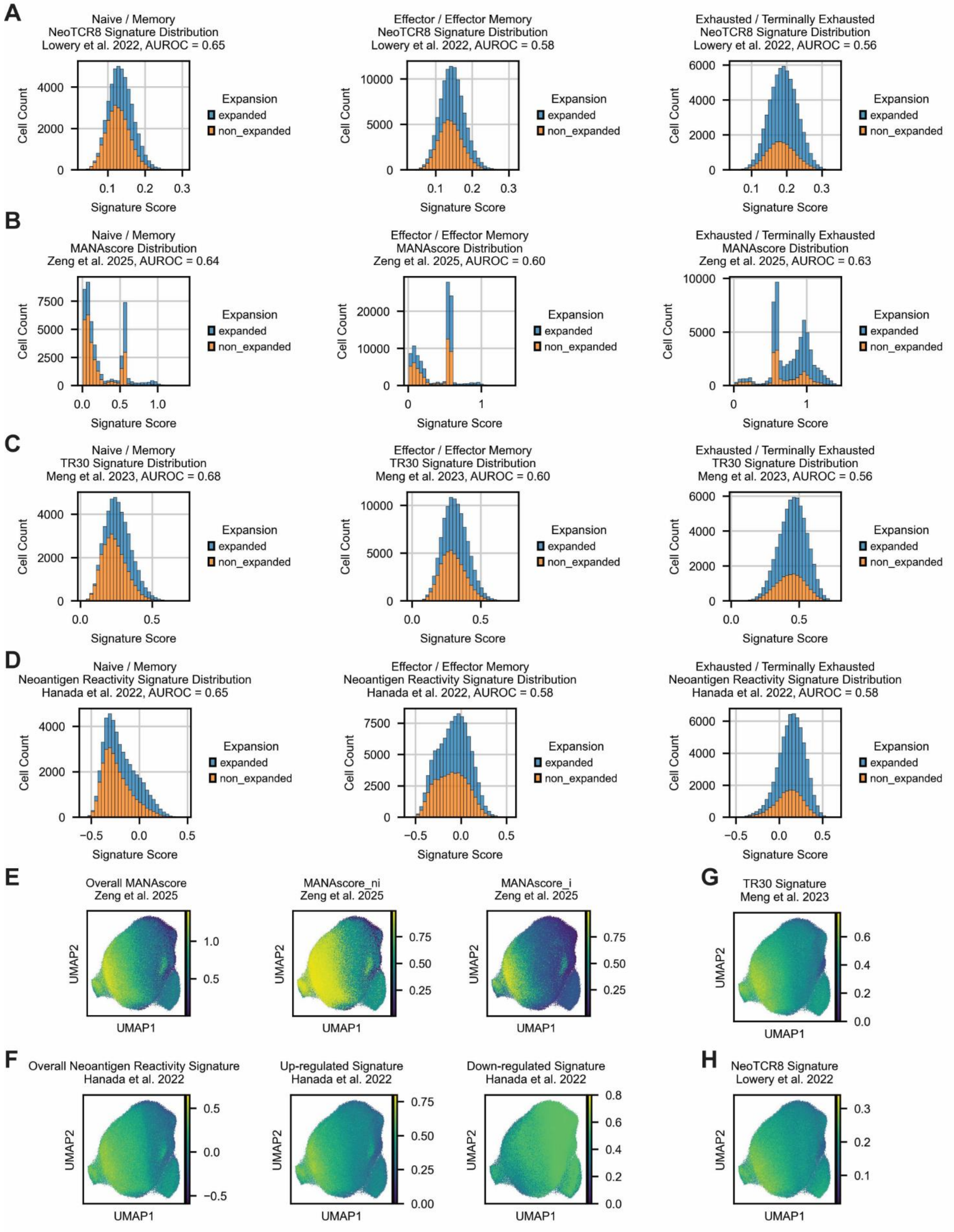
A
Naive / Memory
NeoTCR8 Signature Distribution
Lowery et al. 2022, AUROC = 0.65
Effector / Effector Memory
NeoTCR8 Signature Distribution
Lowery et al. 2022, AUROC = 0.58
Exhausted / Terminally Exhausted
NeoTCR8 Signature Distribution
Lowery et al. 2022, AUROC = 0.56
B
Naive / Memory
MANAscore Distribution
Zeng et al. 2025, AUROC = 0.64
Effector / Effector Memory
MANAscore Distribution
Zeng et al. 2025, AUROC = 0.60
Exhausted / Terminally Exhausted
MANAscore Distribution
Zeng et al. 2025, AUROC = 0.63
C
Naive / Memory
TR30 Signature Distribution
Meng et al. 2023, AUROC = 0.68
Effector / Effector Memory
TR30 Signature Distribution
Meng et al. 2023, AUROC = 0.60
Exhausted / Terminally Exhausted
TR30 Signature Distribution
Meng et al. 2023, AUROC = 0.56
D
Naive / Memory
Neoantigen Reactivity Signature Distribution
Hanada et al. 2022, AUROC = 0.65
Effector / Effector Memory
Neoantigen Reactivity Signature Distribution
Hanada et al. 2022, AUROC = 0.58
Exhausted / Terminally Exhausted
Neoantigen Reactivity Signature Distribution
Hanada et al. 2022, AUROC = 0.58
Cell Count
Signature Score
Expansion
expanded
non_expanded
E
Overall MANAscore
Zeng et al. 2025
MANAscore_ni
Zeng et al. 2025
MANAscore_i
Zeng et al. 2025
G
TR30 Signature
Meng et al. 2023
F
Overall Neoantigen Reactivity Signature
Hanada et al. 2022
Up-regulated Signature
Hanada et al. 2022
Down-regulated Signature
Hanada et al. 2022
H
NeoTCR8 Signature
Lowery et al. 2022
UMAP1
UMAP2

Figure S23. Performance of publicly available signatures of tumor-reactivity[15–18] in predicting clonal expansion across T-cell states, Related to Figure 5. A-D. Histograms depicting the number of T cells in each differentiation state, across the scores obtained using the 'NeoTCR8' signature[15] (A), the 'MANAscore' signature[16] (B), the 'TR30' signature[18] (C), and the neoantigen reactivity signature of Hanada et al.[17] (D). Bars are colored and stacked based on the true expansion labels of each T cell. E-H. UMAP plots demonstrating score distributions across all $CD8^+$ T cells, including imputed MANAscore (MANAscore_i), non-imputed MANAscore (MANAscore_ni), and the overall MANAscore[16] (E), as well as up-regulated, down-regulated, and the overall neoantigen reactivity score by Hanada et al.[17] (F), including the 'TR30' signature[18] (G) and the 'NeoTCR8' signature[15] distribution (H).

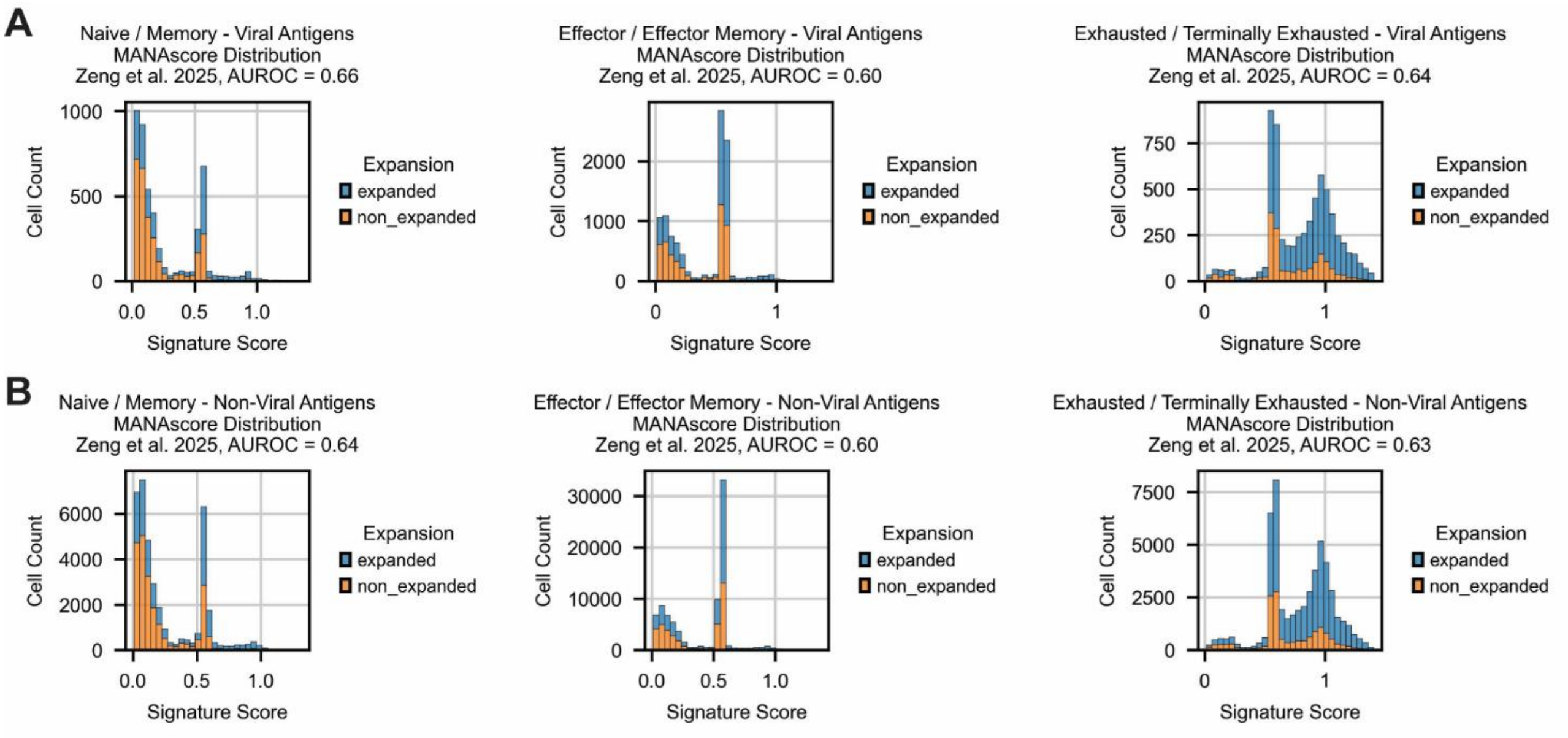

Figure S24. The performance of MANAscore[16] in predicting T-cell clonal expansion across T-cell states for both viral- and non-viral specific T cells, Related to Figure 5. A-B. Histograms depicting the number of T cells in each differentiation state, across the score obtained using the 'MANAscore' signature[16], for both viral-specific (A) and non-viral specific T cells (B). Bars are colored and stacked based on the true expansion labels of each T cell.

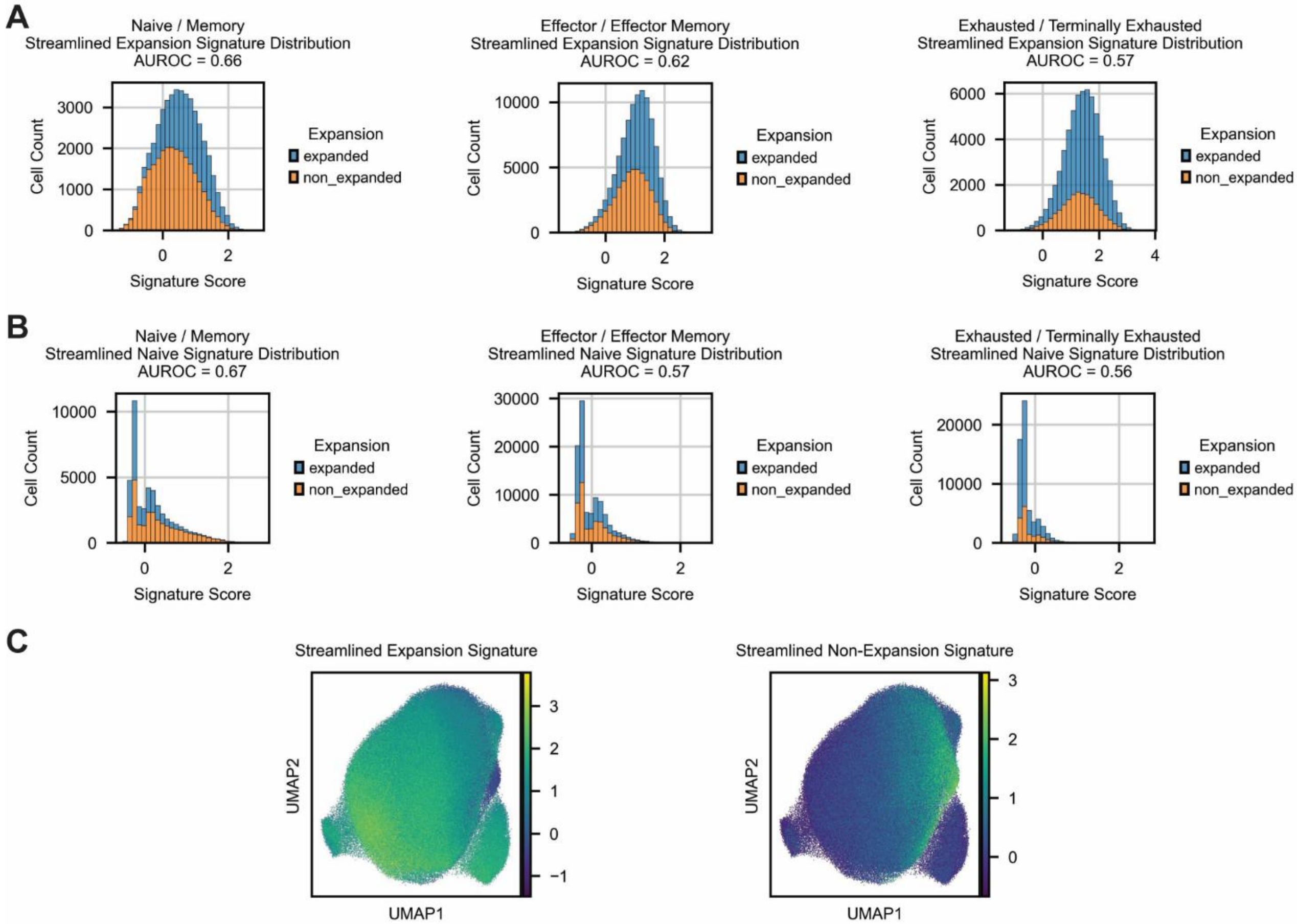

Figure S25. The performance of two streamlined signatures in predicting T-cell clonal expansion across T-cell states, Related to Figure 5. A-B. Histograms depicting the number of T cells in each differentiation state, across the scores obtained using two streamlined signatures, including expansion signature composed of *NKG7*, *CD8A*, *GZMA*, *CST7* and *CXCL13* (A), as well as non-expansion signature composed of *LTB*, *SELL*, *CCR7*, *LEF1* and *MAL* (B). Bars are colored and stacked based on the true expansion labels of each T cell. C. UMAP plots demonstrating both score distributions across all $CD8^+$ T cells.

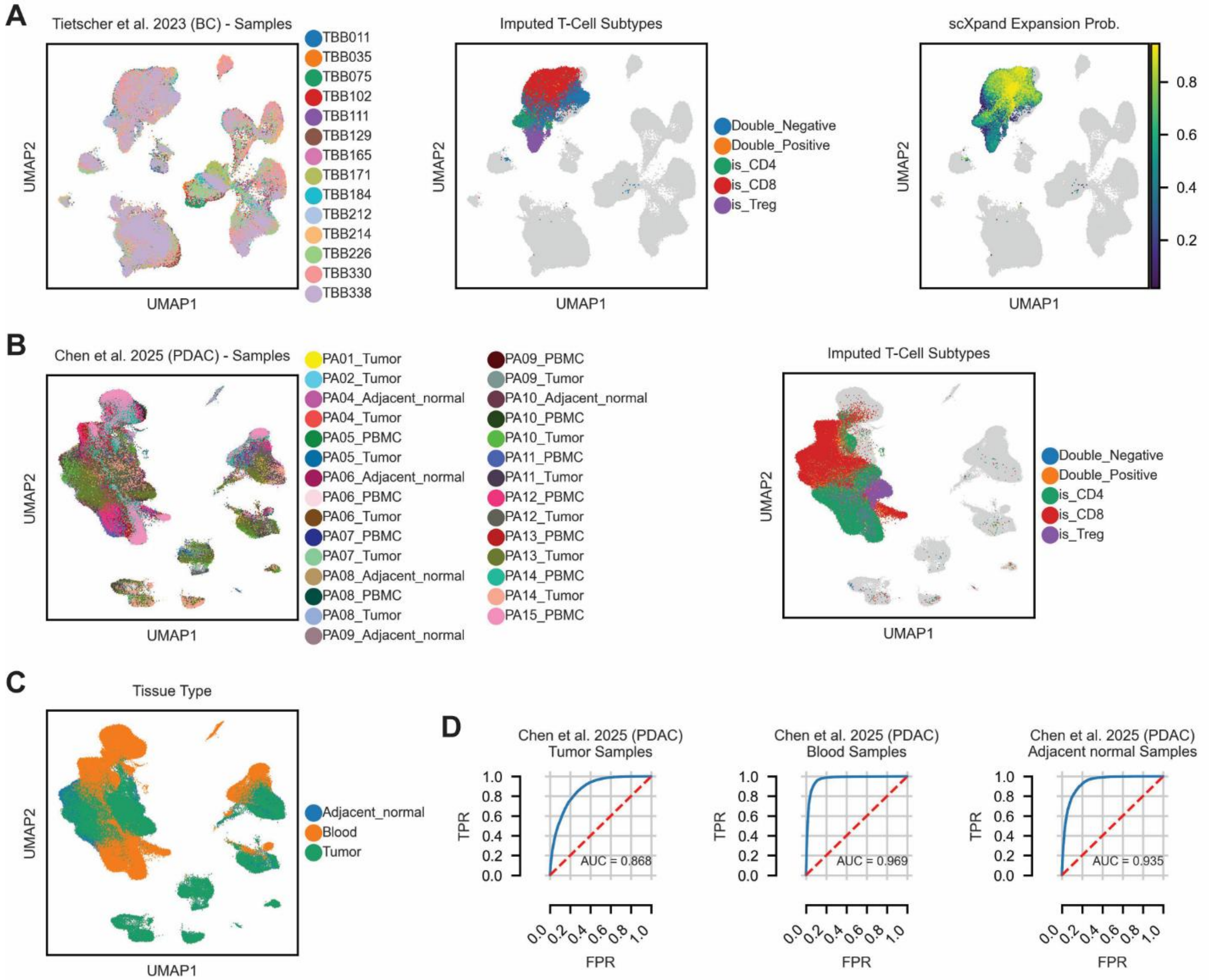

Figure S26. Applicable examples of using the scXpand framework with two datasets, Related to Figure 6. A. UMAP plots of single cells from a publicly available dataset of breast cancer (BC) patients[19], colored by attribution of single cells to samples and T-cell subtypes, as well as the expansion probability per cell predicted by the autoencoder model. B. UMAP plots of single cells from a publicly available dataset of pancreatic ductal adenocarcinoma (PDAC) patients[20], colored by attribution of single cells to samples and T-cell subtypes. C. UMAP plot showing the attribution of single cells to different tissue types. D. ROC curves demonstrating the predictive performance of the autoencoder model across the different tissue types. Abbreviations: FPR, false positive rate; TPR, true positive rate.

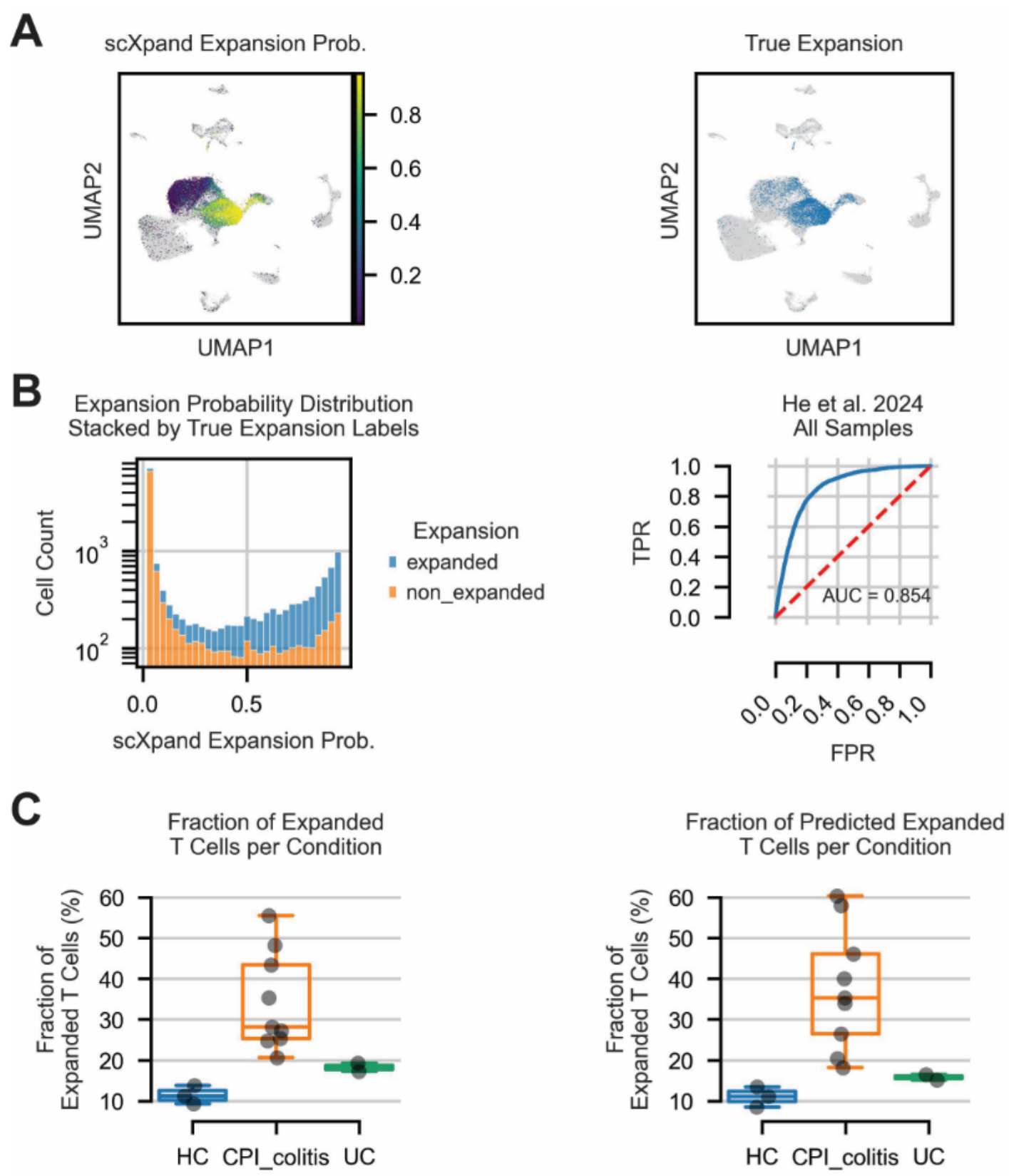

Figure S27. The performance of scXpand in predicting T-cell clonal expansion out of context, using an additional patient cohort by He et al.[21], Related to Figure 6. A. UMAP plots demonstrating the predicted expansion probability for each T cell (left), as well as the true expansion labels (right). B. Histogram depicting the probability distribution stacked by the true expansion labels (left), with the corresponding ROC curve (right). C. Boxplots showing the true fraction of expanded T cells per sample across conditions, out of all the T cells per sample (left), compared to the fractions using clonal expansion predictions (right). Abbreviations: FPR, false positive rate; TPR, true positive rate; HC, healthy control; CPI, checkpoint inhibitors; UC, ulcerative colitis.

Supplemental references:

1. van Dijk, D., Sharma, R., Nainys, J., Yim, K., Kathail, P., Carr, A.J., Burdziak, C., Moon, K.R., Chaffer, C.L., Pattabiraman, D., et al. (2018). Recovering Gene Interactions from Single-Cell Data Using Data Diffusion. Cell *174*, 716-729.e27. https://doi.org/10.1016/j.cell.2018.05.061.

2. McInnes, L., Healy, J., Saul, N., and Großberger, L. (2018). UMAP: Uniform Manifold Approximation and Projection. Journal of Open Source Software *3*, 861. https://doi.org/10.21105/joss.00861.

3. Akiba, T., Sano, S., Yanase, T., Ohta, T., and Koyama, M. (2019). Optuna: A Next-generation Hyperparameter Optimization Framework. In Proceedings of the 25th ACM SIGKDD International Conference on Knowledge Discovery & Data Mining KDD '19. (Association for Computing Machinery), pp. 2623–2631. https://doi.org/10.1145/3292500.3330701.

4. Chicco, D., and Jurman, G. (2020). The advantages of the Matthews correlation coefficient (MCC) over F1 score and accuracy in binary classification evaluation. BMC Genomics *21*, 6. https://doi.org/10.1186/s12864-019-6413-7.

5. Chicco, D., and Jurman, G. (2023). The Matthews correlation coefficient (MCC) should replace the ROC AUC as the standard metric for assessing binary classification. BioData Mining *16*, 4. https://doi.org/10.1186/s13040-023-00322-4.

6. Bagaev, D.V., Vroomans, R.M.A., Samir, J., Stervbo, U., Rius, C., Dolton, G., Greenshields-Watson, A., Attaf, M., Egorov, E.S., Zvyagin, I.V., et al. (2020). VDJdb in 2019: database extension, new analysis infrastructure and a T-cell receptor motif compendium. Nucleic Acids Research *48*, D1057–D1062. https://doi.org/10.1093/nar/gkz874.

7. Guo, X., Nie, H., Zhang, W., Li, J., Ge, J., Xie, B., Hu, W., Zhu, Y., Zhong, N., Zhang, X., et al. (2025). Contrasting cytotoxic and regulatory T cell responses underlying distinct clinical outcomes to anti-PD-1 plus lenvatinib therapy in cancer. Cancer Cell *43*, 248-268.e9. https://doi.org/10.1016/j.ccell.2025.01.001.

8. Shu, D.H., Ho, W.J., Kagohara, L.T., Girgis, A., Shin, S.M., Danilova, L., Lee, J.W., Sidiropoulos, D.N., Mitchell, S., Munjal, K., et al. (2024). Immunotherapy response induces divergent tertiary lymphoid structure morphologies in hepatocellular carcinoma. Nat Immunol *25*, 2110–2123. https://doi.org/10.1038/s41590-024-01992-w.

9. Liu, T., Liu, C., Yan, M., Zhang, L., Zhang, J., Xiao, M., Li, Z., Wei, X., and Zhang, H. (2022). Single cell profiling of primary and paired metastatic lymph node tumors in breast cancer patients. Nat Commun *13*, 6823. https://doi.org/10.1038/s41467-022-34581-2.

10. Wang, A.Z., Mashimo, B.L., Schaettler, M.O., Sherpa, N.D., Leavitt, L.A., Livingstone, A.J., Khan, S.M., Li, M., Anzaldua-Campos, M.I., Bradley, J.D., et al. (2024). Glioblastoma-Infiltrating CD8+ T Cells Are Predominantly a Clonally Expanded GZMK+ Effector Population. Cancer Discov *14*, 1106–1131. https://doi.org/10.1158/2159-8290.CD-23-0913.

11. Liu, Y., Zhang, Q., Xing, B., Luo, N., Gao, R., Yu, K., Hu, X., Bu, Z., Peng, J., Ren, X., et al. (2022). Immune phenotypic linkage between colorectal cancer and liver metastasis. Cancer Cell *40*, 424-437.e5. https://doi.org/10.1016/j.ccell.2022.02.013.

12. Baek, S., Sung, E., Kim, G., Hong, M.H., Lee, C.Y., Shim, H.S., Park, S.Y., Kim, H.R., and Lee, I. (2025). Single-cell multi-omics reveals tumor microenvironment factors underlying poor immunotherapy responses in ALK-positive lung cancer. Cancer Communications *45*, 422–427. https://doi.org/10.1002/cac2.12658.

13. Liu, C., Chen, Y., Li, X., Bai, Z., Jiang, M., Sheng, D., Zou, W., Huang, R., Huang, Q., Wang, F., et al. (2025). Pre-immunotherapy alters stereotactic ablative radiotherapy-induced systemic T cell responses in early-stage NSCLC. Cancer Immunol Immunother *74*, 80. https://doi.org/10.1007/s00262-024-03935-8.

14. Zheng, Y., Chen, Z., Han, Y., Han, L., Zou, X., Zhou, B., Hu, R., Hao, J., Bai, S., Xiao, H., et al. (2020). Immune suppressive landscape in the human esophageal squamous cell carcinoma microenvironment. Nat Commun *11*, 6268. https://doi.org/10.1038/s41467-020-20019-0.

15. Lowery, F.J., Krishna, S., Yossef, R., Parikh, N.B., Chatani, P.D., Zacharakis, N., Parkhurst, M.R., Levin, N., Sindiri, S., Sachs, A., et al. (2022). Molecular signatures of antitumor neoantigen-reactive T cells from metastatic human cancers. Science *375*, 877–884. https://doi.org/10.1126/science.abl5447.

16. Zeng, Z., Zhang, T., Zhang, J., Li, S., Connor, S., Zhang, B., Zhao, Y., Wilson, J., Singh, D., Kulikauskas, R., et al. (2025). A minimal gene set characterizes TIL specific for diverse tumor antigens across different cancer types. Nat Commun *16*, 1070. https://doi.org/10.1038/s41467-024-55059-3.

17. Hanada, K., Zhao, C., Gil-Hoyos, R., Gartner, J.J., Chow-Parmer, C., Lowery, F.J., Krishna, S., Prickett, T.D., Kivitz, S., Parkhurst, M.R., et al. (2022). A phenotypic signature that identifies neoantigen-reactive T cells in fresh human lung cancers. Cancer Cell *40*, 479-493.e6. https://doi.org/10.1016/j.ccell.2022.03.012.

18. Meng, Z., Rodriguez Ehrenfried, A., Tan, C.L., Steffens, L.K., Kehm, H., Zens, S., Lauenstein, C., Paul, A., Schwab, M., Förster, J.D., et al. (2023). Transcriptome-based identification of tumor-reactive and bystander CD8+ T cell receptor clonotypes in human pancreatic cancer. Science Translational Medicine *15*, eadh9562. https://doi.org/10.1126/scitranslmed.adh9562.

19. Tietscher, S., Wagner, J., Anzeneder, T., Langwieder, C., Rees, M., Sobottka, B., de Souza, N., and Bodenmiller, B. (2023). A comprehensive single-cell map of T cell exhaustion-associated immune environments in human breast cancer. Nature Communications *14*. https://doi.org/10.1038/s41467-022-35238-w.

20. Chen, M.-M., Gao, Q., Ning, H., Chen, K., Gao, Y., Yu, M., Liu, C.-Q., Zhou, W., Pan, J., Wei, L., et al. (2025). Integrated single-cell and spatial transcriptomics uncover distinct cellular subtypes involved in neural invasion in pancreatic cancer. Cancer Cell *43*, 1656-1676.e10. https://doi.org/10.1016/j.ccell.2025.06.020.

21. He, J.Y., Kim, Y.-J., Mennillo, E., Rusu, I., Bain, J., Rao, A.A., Andersen, C., Law, K., Yang, H., Tsui, J., et al. (2024). Dysregulation of CD4+ and CD8+ resident memory T, myeloid, and stromal cells in steroid-experienced, checkpoint inhibitor colitis. J Immunother Cancer *12*. https://doi.org/10.1136/jitc-2023-008628.
